# Psychological Distress Before and During the COVID-19 Pandemic Among Adults in the United Kingdom Based on Coordinated Analyses of 11 Longitudinal Studies

**DOI:** 10.1001/jamanetworkopen.2022.7629

**Published:** 2022-04-22

**Authors:** Kishan Patel, Elaine Robertson, Alex S. F. Kwong, Gareth J. Griffith, Kathryn Willan, Michael J. Green, Giorgio Di Gessa, Charlotte F. Huggins, Eoin McElroy, Ellen J. Thompson, Jane Maddock, Claire L. Niedzwiedz, Morag Henderson, Marcus Richards, Andrew Steptoe, George B. Ploubidis, Bettina Moltrecht, Charlotte Booth, Emla Fitzsimons, Richard Silverwood, Praveetha Patalay, David Porteous, Srinivasa Vittal Katikireddi

**Affiliations:** 1 MRC Unit for Lifelong Health and Ageing, University College London, London, England, United Kingdom; 2 MRC/CSO Social & Public Health Sciences Unit, University of Glasgow, Glasgow, Scotland; 3 Division of Psychiatry, University of Edinburgh, Edinburgh, Scotland; 4 MRC Integrative Epidemiology Unit, University of Bristol, Bristol, England; 5 Population Health Sciences, Bristol Medical School, University of Bristol, Bristol, England; 6 Bradford Institute for Health Research, United Kingdom; 7 Department of Epidemiology and Public Health, University College London, London, England, United Kingdom; 8 Institute of Genetics and Cancer, University of Edinburgh, Edinburgh, Scotland; 9 Department of Neuroscience, Psychology and Behaviour, University of Leicester, Leicester, England; 10 Department of Twin Research and Genetic Epidemiology, School of Life Course & Population Sciences, King’s College London, London, England; 11 Institute of Health & Wellbeing, University of Glasgow, Glasgow, Scotland; 12 Centre for Longitudinal Studies, University College London, London, England, United Kingdom; 13 Centre for Medical Information, University of Edinburgh, Edinburgh, Scotland

## Abstract

**Importance:**

How population mental health has evolved across the COVID-19 pandemic under varied lockdown measures is poorly understood, and the consequences for health inequalities are unclear.

**Objective:**

To investigate changes in mental health and sociodemographic inequalities from before and across the first year of the COVID-19 pandemic in 11 longitudinal studies.

**Design, Setting, and Participants:**

This cohort study included adult participants from 11 UK longitudinal population-based studies with prepandemic measures of psychological distress. Analyses were coordinated across these studies, and estimates were pooled. Data were collected from 2006 to 2021.

**Exposures:**

Trends in the prevalence of poor mental health were assessed in the prepandemic period (time period 0 [TP 0]) and at 3 pandemic TPs: 1, initial lockdown (March to June 2020); 2, easing of restrictions (July to October 2020); and 3, a subsequent lockdown (November 2020 to March 2021). Analyses were stratified by sex, race and ethnicity, education, age, and UK country.

**Main Outcomes and Measures:**

Multilevel regression was used to examine changes in psychological distress from the prepandemic period across the first year of the COVID-19 pandemic. Psychological distress was assessed using the 12-item General Health Questionnaire, the Kessler 6, the 9-item Malaise Inventory, the Short Mood and Feelings Questionnaire, the 8-item or 9-item Patient Health Questionnaire, the Hospital Anxiety and Depression Scale, and the Centre for Epidemiological Studies–Depression across different studies.

**Results:**

In total, 49 993 adult participants (12 323 [24.6%] aged 55-64 years; 32 741 [61.2%] women; 4960 [8.7%] racial and ethnic minority) were analyzed. Across the 11 studies, mental health deteriorated from prepandemic scores across all 3 pandemic periods, but there was considerable heterogeneity across the study-specific estimated effect sizes (pooled estimate for TP 1: standardized mean difference [SMD], 0.15; 95% CI, 0.06-0.25; TP 2: SMD, 0.18; 95% CI, 0.09-0.27; TP 3: SMD, 0.21; 95% CI, 0.10-0.32). Changes in psychological distress across the pandemic were higher in women (TP 3: SMD, 0.23; 95% CI, 0.11, 0.35) than men (TP 3: SMD, 0.16; 95% CI, 0.06-0.26) and lower in individuals with below–degree level education at TP 3 (SMD, 0.18; 95% CI, 0.06-0.30) compared with those who held degrees (SMD, 0.26; 95% CI, 0.14-0.38). Increased psychological distress was most prominent among adults aged 25 to 34 years (SMD, 0.49; 95% CI, 0.14-0.84) and 35 to 44 years (SMD, 0.35; 95% CI, 0.10-0.60) compared with other age groups. No evidence of changes in distress differing by race and ethnicity or UK country were observed.

**Conclusions and Relevance:**

In this study, the substantial deterioration in mental health seen in the UK during the first lockdown did not reverse when lockdown lifted, and a sustained worsening was observed across the pandemic period. Mental health declines have been unequal across the population, with women, those with higher degrees, and those aged 25 to 44 years more affected than other groups.

## Key Points

**Question:**

How has the mental health of the UK population changed from before to during the COVID-19 pandemic?

**Findings:**

This cohort study of 49 993 participants in 11 longitudinal studies found that mental health has deteriorated from before the start of the COVID-19 pandemic, and this deterioration was sustained across the first year of the pandemic. Deterioration in mental health varied by sociodemographic factors, namely age, sex, and education, and did not recover when social restrictions were eased.

**Meaning:**

The substantial deterioration in mental health during the ongoing COVID-19 pandemic observed in this study highlights the need for improved mental health care provision and broader support to minimize the risk of longer-term mental health consequences and widening health inequalities.

## Teaser

This cohort study investigates changes in mental health and sociodemographic inequalities from before and across the first year of the COVID-19 pandemic in 11 longitudinal studies in the United Kingdom.

## Introduction

There have been widespread concerns about the impact of the COVID-19 pandemic and related mitigation measures on population mental health.^1,2^ Globally, there is evidence that the pandemic has resulted in poorer mental health,^3^ but much of this might depend on COVID-19 rates and the varying mitigation policies implemented. Concerns exist that specific policy responses, notably so-called lockdown measures, may themselves adversely affect mental health. Examining changes from before the pandemic, but also across different pandemic periods with different restrictions in place, may help understand the factors associated with adverse mental health effects.

Reports on population mental health changes at the start of the pandemic within the United Kingdom are conflicting, with some studies indicating a widespread decline in psychological well-being early on,^4^ while other studies suggest improvements or no changes in mental ill health.^5,6^ Findings have remained inconsistent as the pandemic has progressed, with both increasing and decreasing levels of poorer mental health reported.^7,8,9^

The COVID-19 pandemic has had disproportionate impacts on different age and sociodemographic groups via different mechanisms.^10,11^ For instance, older adults were at greater risk of severe disease and were asked to stay at home and minimize face-to-face contact (shielding), while younger people, women, and racial and ethnic minority groups have been disproportionately affected by employment losses and precarity.^12^ The focus of many existing studies is on population averages, which may have concealed inequalities in mental health outcomes.^3^

Uncertainty remains about how mental health has changed over the pandemic, including who has been most affected and whether any observed deterioration reflects lockdown measures or other aspects of the pandemic. To examine this, we conducted coordinated analyses of 11 UK longitudinal population studies with data from before and across the pandemic. We aimed to (1) estimate the consequences of the pandemic on population mental health and how these evolved during the first year of the pandemic as lockdown restrictions changed and (2) examine inequalities in these impacts by age, sex, race and ethnicity, education level, and UK country.

## Methods

### Design

The UK National Core Studies–Longitudinal Health and Well-being initiative aims to coordinate primary analyses across multiple UK longitudinal population-based studies.^13,14^ Coordinating analyses across different data sets minimizes methodological heterogeneity and maximizes comparability, while appropriately accounting for the study design and characteristics of individual data sets. Reporting followed the Strengthening the Reporting of Observational Studies in Epidemiology (STROBE) reporting guideline.

### Participants

Data were pooled from 11 UK longitudinal population studies that conducted surveys both before and during the COVID-19 pandemic. Details of the design, sampling frames, current age range, timing of the prepandemic and COVID-19 surveys, response rates, and analytical sample size are in the Table,^15,16,17,18,19,20,21,22,23,24,25,26,27,28,29,30,31,32,33,34^ with further details of each analytical sample in eTable 3 in the Supplement. Ethical approvals were received for all included studies, with ethics statements described in eAppendix 1 of the Supplement. All studies collected informed consent from their participants. This study did not seek any additional institutional review board approval.

**Table.** Details of Each Included Study

Study	Design and sample frame	Age range in 2020, y	Most recent prepandemic survey	Details of COVID-19 surveys (response rate)	Mental distress measure used	Analytic sample size, No.
**Age-homogenous cohorts**						
Millennium Cohort Study (MCS)^15^	Cohort of UK children born between September 2000 and January 2002 with regular follow-up surveys from birth	18-20	2018	3 surveys: May 2020 (26.6%); September to October 2020 (24.2%); February to March 2021 (22%)	6-Item Kessler^16^	4988
Avon Longitudinal Study of Parents and Children—Generation 1 (ALSPAC)^17^	Cohort of children born in the Southwest of England between April 1991 and December 1992, with regular follow-up questionnaires from birth	27-29	2017-2018	3 surveys: April 2020 (19%); June 2020 (17.4%); December 2020 (26.4%)	Short Mood and Feelings Questionnaire^18^	3208
Next Steps (NS), formerly known as Longitudinal Study of Young People in England^19^	Sample recruited via secondary schools in England at approximately age 13 y with regular follow-up surveys thereafter	29-31	2015	3 surveys: May 2020 (20.3%); September to October 2020 (31.8%); February to March 2021 (29%)	12-Item General Health Questionnaire^20^	4139
British Cohort Study 1970 (BCS70)^21^	Cohort of all children born in Great Britain (ie, England, Wales, and Scotland) in 1 week in 1970, with regular follow-up surveys from birth	50	2016	3 surveys: May 2020 (40.4%); Sep to Oct 2020 (43.9%); Feb to Mar 2021 (40%)	9-item Malaise inventory^22^	5532
National Child Development Study (NCDS)^23^	Cohort of all children born in Great Britain (ie, England, Wales, and Scotland) in 1 week in 1958, with regular follow-up surveys from birth	62	2013	3 surveys: May 2020 (57.9%); Sep to Oct 2020 (53.9%); Feb to Mar 2021 (52%)	9-item Malaise inventory^22^	6667
National Survey of Health and Development (NSHD)^24^	Cohort of all children born in Great Britain (ie, England, Wales, and Scotland) in 1 week in 1946, with regular follow-up surveys from birth	74	2015	3 surveys: May 2020 (68.2%); September to October 2020 (61.5%); February to March 2021 (90%)	12-Item General Health Questionnaire^20^	2007
**Age-heterogeneous studies**						
Understanding Society: the UK Household Longitudinal Survey (USOC)^25^	A nationally representative longitudinal household panel study, based on a clustered-stratified probability sample of UK households, with all adults aged ≥16 y in chosen households surveyed annually	16-96	2018-2019	7 surveys: April 2020 (40.3%); May 2020 (33.6%); June 2020 (32.0%); July 2020 (31.2%); September 2020 (29.2%); November 2020 (27.3%); January 2021 (27.2%)	12-Item General Health Questionnaire^20^	12 437
English Longitudinal Study of Aging (ELSA)^26^	A nationally representative population study of individuals aged ≥50 living in England, with biennial surveys and periodic refreshing of the sample to maintain representativeness	52 to ≥90	2018-2019	2 surveys: June to July 2020 (75%); November to December 2020 (73%)	Centre for Epidemiological Studies–Depression^27^	5699
Generation Scotland: The Scottish Family Health Study (GS)^28^	A family-structured, population-based Scottish cohort, with participants aged 18-99 y recruited between 2006-2011	27-100	2006-2011	3 surveys: April to June 2020 (21.3%); July to August 2020 (15.4%); February 2021 (14.3%)	9-Item Patient Health Questionnaire^29^ or 8-item Patient Health Questionnaire^30^ and 7-item Generalized Anxiety Disorder Assessment^31^	4151
UK Adult Twin Registry (TwinsUK)^32^	A cohort of volunteer adult TwinsUK (55% monozygotic and 43% dizygotic) from around the United Kingdom who were sampled between ages 18 and 101 y	22-96	2017-2018	3 surveys: April 2020 (64.3%); July 2020 (77.6%); November 2020 (76.1%)	Hospital and Anxiety Depression Scale^33^	4040
Born in Bradford (BiB)^34^	2 birth cohorts recruiting pregnant women and their children between 2007 and 2010 (BiB Growing Up) and from 2016 (Born in Bradford’s Better Start [BiBBS])	16-57	2016-2020	2 surveys: April to June 2020 (28%); October to November 2020 (35.8%)	9-Item Patient Health Questionnaire^29^ or 8-item Patient Health Questionnaire^30^ and 7-item Generalized Anxiety Disorder Assessment^31^	1967

Six studies were age-homogenous cohorts (ie, similarly aged individuals): the Millennium Cohort Study (MCS)^15^; children in the Avon Longitudinal Study of Parents and Children (ALSPAC)^17^; Next Steps (NS; formerly known as the Longitudinal Study of Young People in England)^19^; 1970 British Cohort Study (BCS70)^21^; 1958 National Child Development Study (NCDS)^23^; and 1946 National Survey of Health and Development (NSHD).^24^ Five other studies had age-heterogeneous samples (ie, cohorts with multiple age groups): Understanding Society (USOC)^25^; Generation Scotland (GS)^28^; Twins UK (TwinsUK)^32^; Born in Bradford (BiB)^34^; and the English Longitudinal Study of Aging (ELSA).^26^

Analytical samples included those who had valid observations of psychological distress in a prepandemic survey, at least 1 survey during the pandemic, and valid data on sex and age (participant flow diagrams for each study appear in eAppendix 3 in the Supplement). Participants who had died or emigrated by the start of the pandemic were also excluded. Most studies were weighted to be representative of their target population, accounting for sampling design and differential nonresponse to the COVID-19 surveys.^35,36,37^ Weights were not used for ALSPAC, TwinsUK, GS, and BiB.

### Measures

In the following sections, we describe the variables used for analysis. Details of the specific scales and coding used within each cohort appear in eAppendix 2 in the Supplement.

#### Mental Health

Psychological distress was measured both before the pandemic and at multiple points across the pandemic using validated, continuous scales measuring symptoms of common mental health disorders, such as depression and anxiety (specific measures used appear in the Table). Continuous scales were standardized across time points and within studies on a common SD-based scale. This enhances comparability of estimates between studies while allowing examination of changes over time within studies. We also conducted analyses with dichotomous indicators of high psychological distress using established thresholds for each scale (eTable 1 and eTable 2 in the Supplement).

While most studies used the same measure for both prepandemic and COVID-19 surveys, GS and NSHD used different measures. For these studies, we identified comparable items to create a smaller scale consistent over time, and the threshold for the binary outcome was reweighted based on the number of items retained (eTable 1 in the Supplement).

#### Pandemic Time Period

We identified 3 time periods (TPs 1-3) representing different stages during the course of the pandemic in the United Kingdom for comparison against prepandemic mental health (measured at TP 0). Surveys from April to June 2020 represented the first wave of high infection levels accompanied by the first lockdown measures (TP 1). Surveys taken from July to October 2020 coincided with easing of restrictions and lower rates of infection (TP 2). Following this, infection levels again increased, and lockdown measures were reintroduced; surveys taken from November 2020 to March 2021 represent this second wave of infections (TP 3). Some studies contributed multiple survey waves to some TPs, and not all studies were represented in all 3 COVID-19 TPs (Table).

#### Covariates

The following covariates were adjusted for and/or used to stratify estimates: sex (male or female); age in the age-heterogeneous cohorts (coded in 10-year bands to examine nonlinearity: 16-24, 25-34, 35-44, 45-54, 55-64, 65-74, and ≥75 years); race and ethnicity (self-reported and coded for main analyses; as White [including White ethnic minorities] vs racial and ethnic minority groups ); UK country of residence (England, Scotland, Wales, or Northern Ireland); and highest educational qualification (degree vs less than degree; parental education was used for the MCS cohort, who had not all completed their full-time education). Due to small sample sizes or lack of available ethnicity breakdown, we are we are unable to report race and ethnicity findings in more detail.

### Statistical Analysis

Changes in continuous measures of mental health over the 3 TPs were modeled using multilevel mixed-effects models within each study to account for associations between repeated measures from the same individuals, adjusting for sex and age (in age-heterogeneous cohorts). TP was a categorical exposure, with TP 0 as the reference. In some studies, multiple survey waves were included within the same TP. Coefficients are presented as standardized mean differences (SMDs). Multilevel mixed-effects Poisson regression models with robust standard errors were used to calculate relative risks for the binary outcome.^38^

Results from each study were pooled using a random-effects meta-analysis with restricted maximum likelihood. Meta-analyses were conducted separately for continuous psychological distress scores and binary high psychological distress thresholds. Heterogeneity is reported using the *I*^2^ statistic.^39^

Interactions between TP and sex, education, and race and ethnicity were estimated within each study and then meta-analyzed to formally test for effect modification (ie, to determine whether changes across time periods varied between population subgroups). Formal interactions could not be tested by age and UK country given the age-homogeneous nature of several cohorts and few studies including all UK nations. We present meta-analysis of estimates stratified by sex, education, ethnicity, age, and UK country.

Further sensitivity meta-analyses restricted analyses to include studies that only assessed anxiety specifically, that assessed depression specifically, and that included survey responses for all 3 TPs. To explore the heterogeneity in estimates, metaregression analyses were conducted, quantifying the association of time with prepandemic and postpandemic measures, measurement type, and whether study samples were representative of their target age range in the UK population (eTable 26 in the Supplement). All meta-analyses and metaregressions were conducted using Stata version 17 (StataCorp). No prespecified level of significance was set.

## Results

Across 11 individual longitudinal studies, 49 993 participants (12 323 [24.6%] aged 55-64 years; 32 741 [61.2%] women; 4960 [8.7%] racial and ethnic minority) were analyzed, ranging from 1816 participants in NSHD to 12 437 in USOC. The proportion of women ranged between 7208 (52.1%) in USOC to 1967 (100.0%) for BiB, and racial and ethnic minority participants ranged from 26 of 4103 (0.6%) in GS to 1223 (62.2%) in BiB. Descriptive statistics for all the studies, weighted and taking account of complex survey design where relevant, are in eTable 3 in the Supplement.

### Descriptive Analysis

**Figure 1.**
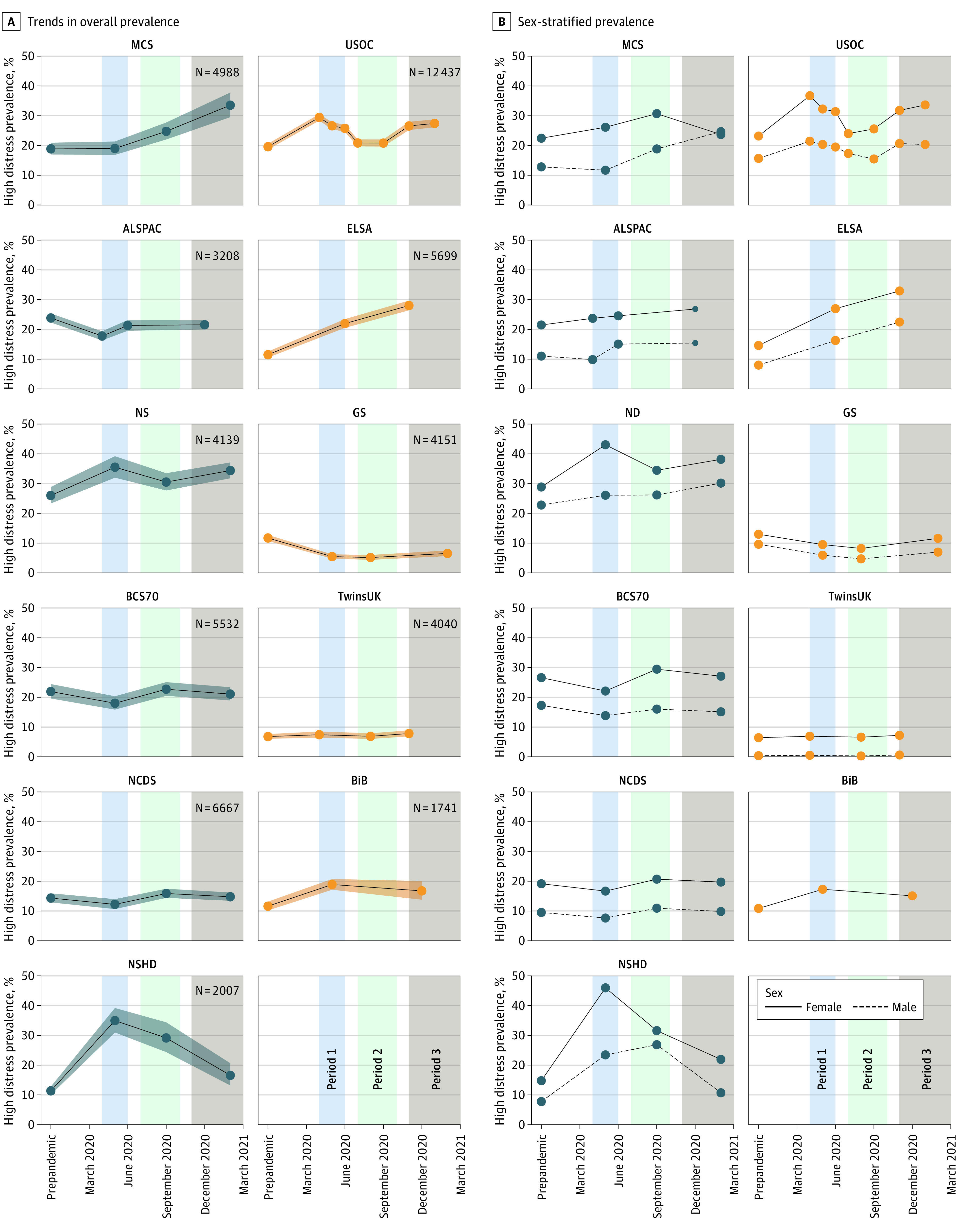
Trends in Overall and Sex-Stratified Prevalence of High Psychological Distress Colored boxes indicate the time period groupings, with blue indicating time period 1 (March to June 2020); green, time period 2 (July to October 2020); and gray, time period 3 (November 2020 to March 2021). A, Shaded areas indicate 95% CIs. ALSPAC indicates children in the Avon Longitudinal Study of Parents and Children; BCS70, 1970 British Cohort Study; BiB, Born in Bradford; ELSA, the English Longitudinal Study of Aging; GS, Generation Scotland; MCS, the Millennium Cohort Study; NCDS, 1958 National Child Development Study; NS, Next Steps; NSHD, 1946 National Survey of Health and Development; and USOC, Understanding Society.

Descriptive statistics appear in eTables 4, 5, and 6 in the Supplement. Figure 1A shows that for most studies, prevalence of high psychological distress either worsened or was fairly stable over the course of the pandemic. The largest increase in prevalence of high psychological distress was observed within the ELSA study, rising from 11.5% to 28.0% over the course of the 3 TPs. The largest increase between 2 consecutive TPs was observed within the NSHD study, between the prepandemic (2015) and first pandemic TP, increasing from 11.4% to 35.0%. In 2 studies (ALSPAC and BCS70), the prevalence of distress in the final pandemic TP (TP 3) was marginally lower than in the prepandemic time period (prevalence decreased by 2.3% and 0.8% respectively).

Figure 1B shows the sex difference in mental health over the course of the pandemic, with higher prevalence of distress among women than men in all sex-heterogeneous studies. In April and May 2020 (TP 1), sex inequalities appeared especially high, with female respondents exhibiting higher prevalence of mental distress in most studies. For example, in NSHD at TP 1, 46.0% of female respondents reported mental distress vs 23.5% of male respondents. In NS at TP 1, 43.0% of female respondents reported mental distress vs 26.1% of male respondents.

### Changes in Distress From Before and During the Pandemic: Pooled Analysis

**Figure 2.**
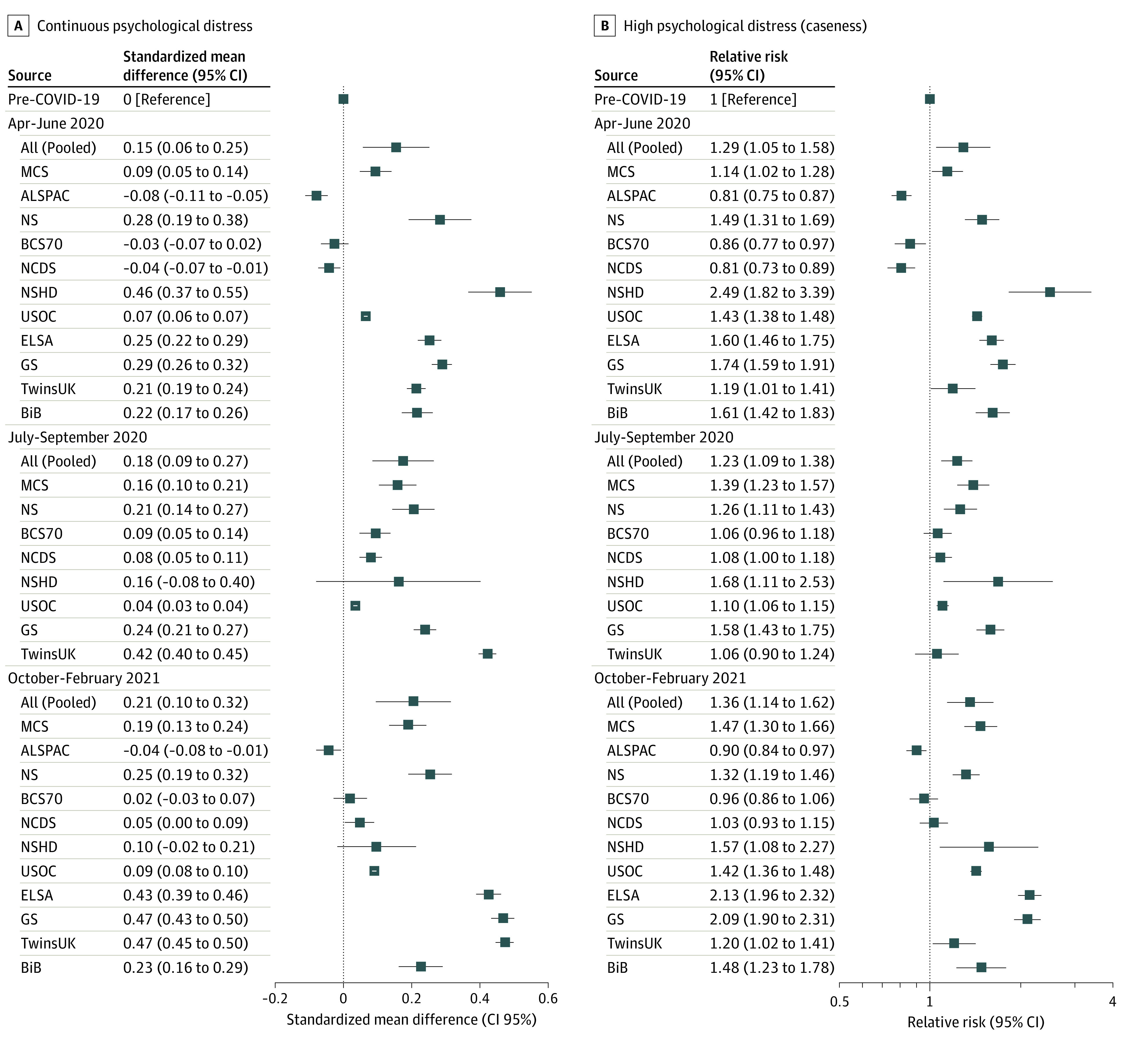
Changes in Psychological Distress Before and During the Pandemic in Each of 11 Longitudinal UK Studies Standardized mean differences measure changes across time periods (compared with prepandemic distress) for the continuous psychological distress scores (A), and relative risk measures risk of high distress scores at each time period (B). ALSPAC indicates children in the Avon Longitudinal Study of Parents and Children; BCS70, 1970 British Cohort Study; BiB, Born in Bradford; ELSA, the English Longitudinal Study of Aging; GS, Generation Scotland; MCS, the Millennium Cohort Study; NCDS, 1958 National Child Development Study; NS, Next Steps, formerly the Longitudinal Study of Young People in England; NSHD, 1946 National Survey of Health and Development; TwinsUK, Twins UK; and USOC, Understanding Society.

Psychological distress increased from prepandemic scores across all 3 pandemic TPs examined (observed in 8 of the 11 included cohorts when focusing on general distress or depressive symptom measures), with no clear differences in changes across the 3 pandemic TPs (TP 1: SMD, 0.15; 95% CI, 0.06-0.25; TP 2: SMD, 0.18; 95% CI, 0.09-0.27; TP 3: SMD, 0.21; 95% CI, 0.10-0.32). However, there was considerable heterogeneity between estimates from different studies (*I*^2 ^of 99.2%, 98.6%, and 99.2% at TP 1, TP 2, and TP 3, respectively), with estimates for TP 1 ranging from an SMD of −0.08 (95% CI, −0.11 to −0.05) for ALSPAC to an SMD of 0.46 (95% CI, 0.37 to 0.55) for NSHD (individual cohort results in eTable 7 in the Supplement). Leave one out meta-analysis found that no single cohort significantly skewed the pooled estimates (eTable 16 and eAppendix 5 in the Supplement). Similar patterns and high levels of heterogeneity were observed when considering prevalence of psychological distress as a binary outcome (eTable 17 in the Supplement). Estimates for both continuous and binary measures of mental distress are displayed in Figure 2. The pooled relative risk of high mental distress was elevated at TP 1 (relative risk, 1.29; 95% CI, 1.05-1.58) and TP 2 (relative risk, 1.23; 95% CI, 1.09-1.38), with the highest risk at TP 3 (relative risk, 1.36; 95% CI, 1.14-1.62).

### Inequalities in Changes Over Time Periods: Pooled Analysis

**Figure 3.**
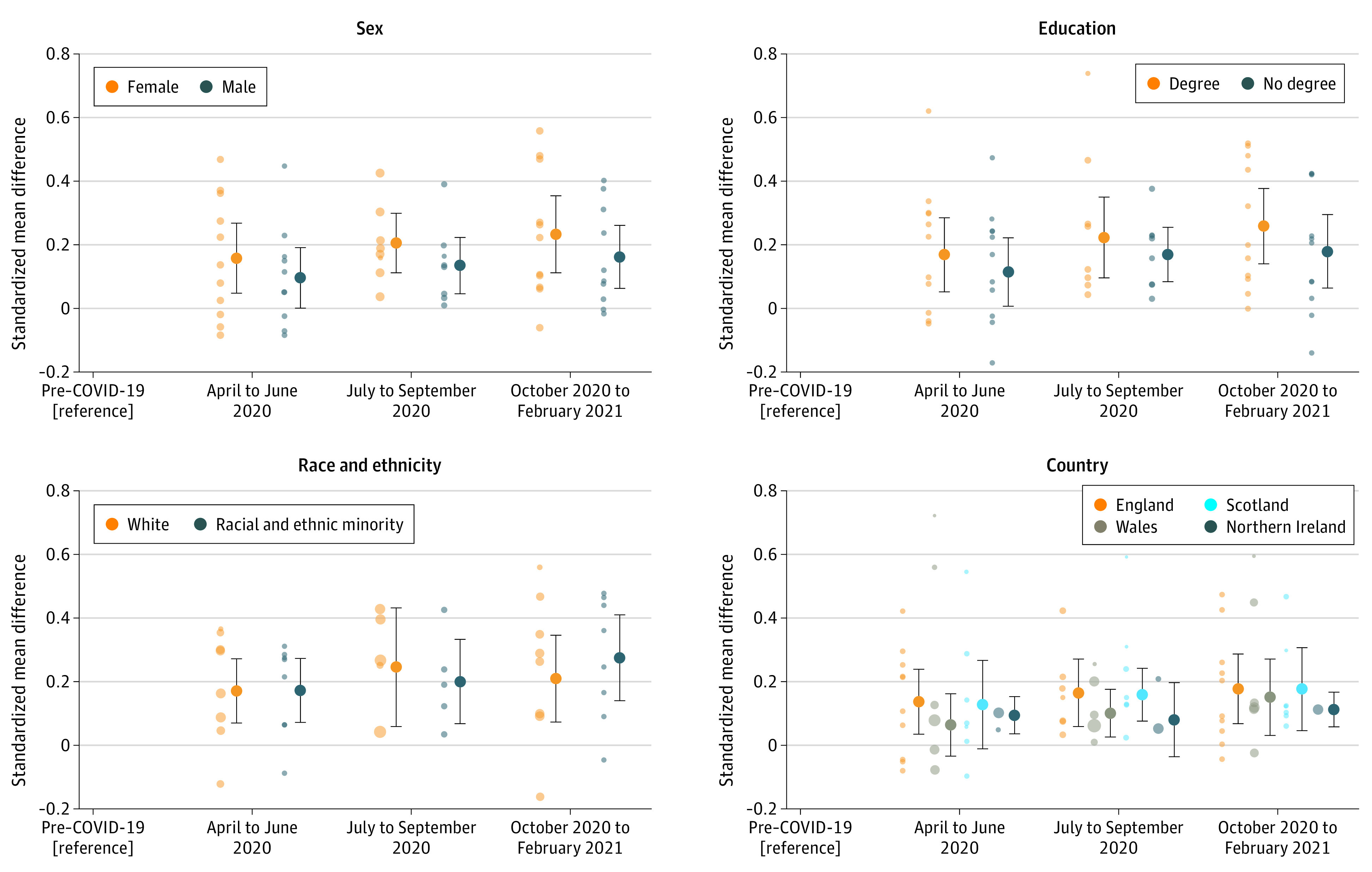
Changes in Psychological Distress Over Time by Sex, Education, Ethnicity, and UK Country Stratified changes across time periods (compared with prepandemic distress). Each light-colored point represents estimates from a different included study (study-specific estimates appear in eTables 8 and 10-12 in the Supplement).

Meta-analysis of the study-specific interaction terms between each marker of inequity and time period (eTable 17 in the Supplement) indicated that changes in distress were greater in women (TP 3: SMD, 0.23; 95% CI, 0.11-0.35) compared with men (TP3: SMD, 0.16; 95% CI, 0.06-0.26) (eTable 8 in the Supplement), suggesting a further widening of sex inequalities. Changes were marginally lower at TP 1 and TP 3 for persons with a below-degree level education (TP 3: SMD, 0.18; 95% CI, 0.06-0.30) compared with those with a degree (TP 3: SMD, 0.26; 95% CI, 0.14-0.38), albeit often from a greater prepandemic inequality, indicating a slight narrowing of educational inequalities during the pandemic. We did not find evidence for trends differing by ethnicity or UK country. Heterogeneity varied across these analyses, with *I*^2^ values ranging from 44.2% for the interaction between education and TP 1 to 88.8% for ethnicity and TP 1. Estimates stratified by sex, ethnicity, education, and UK country are shown in Figure 3. Again, in all analyses there was large heterogeneity between study estimates (eTables 8 and 10-12 in the Supplement).

**Figure 4.**
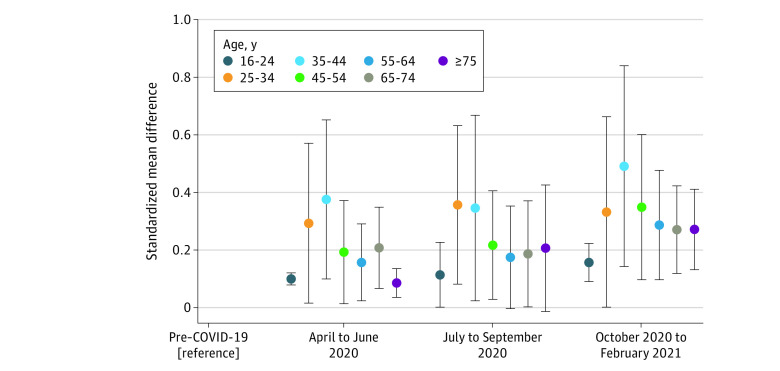
Trends in Prevalence of Psychological Distress by Age Group Stratified changes across time periods (compared with prepandemic distress).

Age-stratified results showed no monotonic pattern by age (Figure 4), despite some suggestion that the consequences of the pandemic on mental health might have been greater in those aged 25 to 44 years. The pooled SMD at TP 3 for those aged 25 to 34 years was 0.49 (95% CI, 0.14-0.84) and for those aged 35 to 44 years, 0.35 (95% CI, 0.10-0.60) (eTable 9 in the Supplement).

### Sensitivity and Metaregression Analyses

Sensitivity analyses were conducted to consider specific measures of mental health (depression or anxiety) and to limit data to participants with survey responses during all 3 TPs. Findings were consistent with the main analyses (eTables 13-15 in the Supplement). We also presented pooled analyses of the binary high distress outcomes, overall and stratified in eTables 17 to 25 in the Supplement.

Given the high levels of heterogeneity across studies, we conducted metaregressions to examine whether time between prepandemic and postpandemic measures, measurement type, and representativeness of the studies for their target population helped account for some of the observed heterogeneity (eTable 28 in the Supplement). Heterogeneity was largely unexplained by these factors; the largest explanatory factor was the representativeness of the studies, which explained 3.25% of the heterogeneity at TP 2 and suggested the deterioration in distress was less marked in representative studies. A subsequent meta-analysis including only studies with national coverage showed a worsening of mental health over the pandemic similar to the main meta-analysis (TP 1: SMD, 0.15; 95% CI, 0.02-0.29; TP 2: SMD, 0.11; 95% CI, 0.05-0.17; TP 3: SMD, 0.16; 95% CI, 0.05-0.27).

## Discussion

Our analyses of 11 well-established longitudinal studies provide a comprehensive picture of the evolution of mental health over the course of differing lockdown periods during the COVID-19 pandemic. Overall, our results indicate mental health has deteriorated since the onset of the pandemic and this has been sustained with no evidence of recovery, even when lockdown measures temporarily eased in the United Kingdom during the summer of 2020. Although evidence for deterioration from prepandemic levels is seen in most included studies, there was considerable heterogeneity in effect sizes estimated. Furthermore, our findings demonstrate that while aggregate population mental health deteriorated over time, not all groups were equally affected. Women, those with a degree-level education, and young adults (aged 25-34 and 35-44 years) were affected most, reporting greater increases in psychological distress during the pandemic and thereby exacerbating some prepandemic mental health inequalities.

Our findings suggest that initial declines in mental health were not a transient reaction to an unprecedented event, but an early indication of a sustained deterioration from prepandemic levels. These findings extend research conducted earlier in the pandemic,^4,40^ replicate some research suggesting sustained effects,^9,41^ and contradict findings from some convenience samples suggesting improvements in mental health when the initial lockdown was lifted.^7^ From a policy perspective, having a wealth of longitudinal data both before and during the COVID-19 pandemic gives further information on how the pandemic has affected mental health, beyond simple convenience sampling data. While the direct mechanisms generating poorer mental health are complex, the COVID-19 pandemic resulted in considerable economic, social, and behavioral changes and an increase in physical comorbidities and bereavement; therefore, increased mental distress is perhaps unsurprising. Financial stressors, changes in social interactions, and disruptions to daily life may all help to explain our findings.^42,43,44,45^ These results suggest that deteriorations in population mental health may be driven more by time-stable disruption and concern arising from the COVID-19 pandemic, rather than the consequences of time-specific mitigation measures such as lockdowns.

Furthermore, this deterioration suggests that avoiding lockdown measures alone may not maintain population mental health, and other factors should be considered. For example, health services in the UK were not able to meet their population’s mental health needs before the pandemic, with this situation made substantially worse during the pandemic.^46^ To minimize the detrimental longer-term consequences of the pandemic, mental health care needs to encompass multiple levels of support, including investment in primary care, community mental health, and public mental health. Initiatives should target groups at greater risk of experiencing mental ill health, including ensuring rapid access to services, but also addressing the underlying drivers of poor mental health, such as mitigating risks of unemployment, sexual violence, and poverty.

Our results highlight widening gender inequalities in mental health. Women had much higher distress levels and showed greater deterioration during the pandemic than men. Possible reasons include increased childcare responsibilities that disproportionately fell to women, greater economic impacts, and reports of large increases in gender-based violence.^47^ We also observed that deterioration in lockdown periods was greater in those with degree-level education, albeit from a lower prepandemic level, indicating that educational inequalities narrowed.^40^ Our investigation of age differences show that all age groups have been adversely affected to some extent, but high psychological distress was greater in those aged 25 to 44 years. The mechanisms underpinning subpopulation differences remain unclear but likely include disruptions to social interactions, changes in employment or education, and shifts in parental responsibilities and/or work-life balance.^48^ For example, individuals between the ages of 25 and 44 years are more likely to have school-aged children and may therefore have faced additional challenges of working from home and caring for children. Moreover, younger adults have been at an increased risk of employment disruptions^49^ as well as changes in healthy behaviors,^50,51^ which may have contributed to further deteriorations in their mental health. However, the well-documented midlife peak in psychological distress is noteworthy,^52^ and may partly explain some of the deterioration we found in these age groups.

The multiple longitudinal studies included in this article highlight the wide range in the size of the estimated deterioration in distress from prepandemic levels across varied data sources that represent different populations. While we explored multiple factors (such as age, outcome measure, timing, and representativeness), we could not explain much of this heterogeneity. Other factors not considered, such as rates of COVID-19 within the samples, might also play a role.

### Strengths and Limitations

Our study has several strengths. By harnessing high-quality existing longitudinal studies, we have robust prepandemic baseline data and multiple waves of data collection capturing different TPs during the pandemic. We investigated the potential consequences of COVID-19 policy responses, specifically the introduction and removal of lockdown measures. Our approach to data harmonization allowed us to develop comparable exposure, outcome, and covariate measures and pool estimates for similar TPs. Furthermore, we maximized the value of existing data by using multilevel models to include all available data. The baseline samples of many of these studies were representative of their target populations, and analyses were weighted to account for nonresponse. Lastly, this study combined 11 longitudinal data sources, and heterogeneity between the study-specific estimates was large, highlighting that documenting the results from multiple sources is more reliable for informing policy and health planning than relying on a single data source.

Despite these advantages, limitations should be noted. We cannot definitively attribute changes in population mental health to the COVID-19 pandemic or related policy responses, as COVID-19 was a universal exposure to everyone. However, we note that we are unaware of alternative events that would have been likely to substantially confound our analyses or their interpretation. There were differences between studies in the timing of data collection (including when prepandemic measures were collected) and the mental health survey instruments used, although this did not account for the high levels of statistical heterogeneity observed. Similarly, although weighting was used when possible to control for nonrandom response, conditioning on voluntary response may induce selection bias, as it is very plausible that the mental health of the observed differs systematically from the target population. However, the broad consistency in the direction of findings across data sets provides reassurance that the key conclusions are likely to be robust to these differences, even if the magnitude of the effect size is harder to confirm.

## Conclusions

The findings of this study suggest that mental health has been persistently worse during the COVID-19 pandemic than before, particularly among women, those with higher degrees, and those aged 25 to 44 years. The sustained deterioration, even when lockdown measures were eased, somewhat refutes the notion that easing lockdown measures necessarily improved mental health and implies that there are myriad pathways leading to adverse mental health outcomes. Our findings highlight the need for investment in mental health support to turn the tide and improve population mental health going forward.

## Notes

Supplement. **eAppendix 1.** Ethics and Data Access Statements For Each Study **eAppendix 2.** Details of Mental Health Measures in Each Longitudinal Study **eAppendix 3.** Participant Flow Diagrams **eTable 1.** Measures of General Psychological Distress in Each Study **eTable 2.** Additional Measures of Anxiety by Study **eTable 3.** Distribution of Participant Characteristics by Study **eTable 4.** Mean Psychological Distress Scores and Percentage With High Psychological Distress, by Study and Over Time **eTable 5.** Mean Additional Anxiety Scores and Percentage With Psychological Distress, by Study and Over Time **eTable 6.** Mean Prepandemic Psychological Distress Scores by Sociodemographic Characteristics and Study **eTable 7.** Meta-analyzed Regression Coefficients: Continuous; Unstratified **eTable 8.** Meta-analyzed Regression Coefficients: Continuous; Stratified by Sex **eTable 9.** Meta-analyzed Regression Coefficients: Continuous; Stratified by Age **eTable 10.** Meta-analyzed Regression Coefficients: Continuous; Stratified by Education **eTable 11.** Meta-analyzed Regression Coefficients: Continuous; Stratified by Education **eTable 12.** Meta-analyzed Regression Coefficients: Continuous; Stratified by Country **eTable 13.** Meta-analyzed Regression Coefficients: Continuous; Sensitivity; Cohorts With All Time Points **eTable 14.** Meta-analyzed Regression Coefficients: Continuous; Sensitivity; Anxiety **eTable 15.** Meta-analyzed Regression Coefficients: Continuous; Sensitivity; Depression **eTable 16.** Meta-analyzed High Psychological Distress When Named Cohort Is Removed **eTable 17.** Meta-analyzed Regression Coefficients: Binary; Unstratified **eTable 18.** Meta-analyzed Regression Coefficients: Binary; Stratified by Sex **eTable 19.** Meta-analyzed Regression Coefficients: Binary; Stratified by Age **eTable 20.** Meta-analyzed Regression Coefficients: Binary; Stratified by Education **eTable 21.** Meta-analyzed Regression Coefficients: Binary; Stratified by Ethnicity **eTable 22.** Meta-analyzed Regression Coefficients: Binary; Stratified by Country **eTable 23.** Meta-analyzed Regression Coefficients: Interactions; Sex **eTable 24.** Meta-analyzed Regression Coefficients: Interactions; Ethnicity **eTable 25.** Meta-analyzed Regression Coefficients: Interactions; Education **eTable 26. **Inclusion and Exclusion Criteria by Cohort for Metaregressions and Sensitivity Analyses **eTable 27.** Meta-analyzed Interaction Coefficients **eTable 28.** Heterogeneity Explained by Measures Explored by Metaregression Analyses **eAppendix 4. **Results of Binary High Psychological Distress Outcomes by Sociodemographic Factor **eAppendix 5.** Meta-analyzed High Psychological Distress When One Cohort Is Removed **eAppendix 6.** Funding Statements for Each Study **eAppendix 7. **ALSPAC Further Study Information
eReferences.
