# Supplementary material for: Psychological Distress Before and During the COVID-19 Pandemic Among Adults in the United Kingdom Based on Coordinated Analyses of 11 Longitudinal Studies
Source: JAMA Netw Open. 2022 Apr 22;5(4):e227629. doi: 10.1001/jamanetworkopen.2022.7629 (PMC9034408; doi:10.1001/jamanetworkopen.2022.7629)
Supplement: Supplement. — eAppendix 1. Ethics and Data Access Statements For Each Study eAppendix 2. Details of Mental Health Measures in Each Longitudinal Study eAppendix 3. Participant Flow Diagrams eTable 1. Measures of General Psychological Distress in Each Study eTable 2. Additional Measures of Anxiety by Study eTable 3. Distribution of Participant Characteristics by Study eTable 4. Mean Psychological Distress Scores and Percentage With High Psychological Distress, by Study and Over Time eTable 5. Mean Additional Anxiety Scores and Percentage With Psychological Distress, by Study and Over Time eTable 6. Mean Prepandemic Psychological Distress Scores by Sociodemographic Characteristics and Study eTable 7. Meta-analyzed Regression Coefficients: Continuous; Unstratified eTable 8. Meta-analyzed Regression Coefficients: Continuous; Stratified by Sex eTable 9. Meta-analyzed Regression Coefficients: Continuous; Stratified by Age eTable 10. Meta-analyzed Regression Coefficients: Continuous; Stratified by Education eTable 11. Meta-analyzed Regression Coefficients: Continuous; Stratified by Education eTable 12. Meta-analyzed Regression Coefficients: Continuous; Stratified by Country eTable 13. Meta-analyzed Regression Coefficients: Continuous; Sensitivity; Cohorts With All Time Points eTable 14. Meta-analyzed Regression Coefficients: Continuous; Sensitivity; Anxiety eTable 15. Meta-analyzed Regression Coefficients: Continuous; Sensitivity; Depression eTable 16. Meta-analyzed High Psychological Distress When Named Cohort Is Removed eTable 17. Meta-analyzed Regression Coefficients: Binary; Unstratified eTable 18. Meta-analyzed Regression Coefficients: Binary; Stratified by Sex eTable 19. Meta-analyzed Regression Coefficients: Binary; Stratified by Age eTable 20. Meta-analyzed Regression Coefficients: Binary; Stratified by Education eTable 21. Meta-analyzed Regression Coefficients: Binary; Stratified by Ethnicity eTable 22. Meta-analyzed Regression Coefficients: Binary; Stratified by Country eTable [file jamanetwopen-e227629-s001.pdf]

Supplemental Online Content

Patel K, Robertson E, Kwong ASF, et al. Psychological distress before and during the COVID-19 pandemic among adults in the United Kingdom based on coordinated analyses of 11 longitudinal studies. *JAMA Netw Open.* 2022;5(4):e227629. doi:10.1001/jamanetworkopen.2022.7629

eAppendix 1. Ethics and Data Access Statements For Each Study

eAppendix 2. Details of Mental Health Measures in Each Longitudinal Study

eAppendix 3. Participant Flow Diagrams

eTable 1 . Measures of General Psychological Distress in Each Study

eTable 2. Additional Measures of Anxiety by Study

eTable 3. Distribution of Participant Characteristics by Study

eTable 4. Mean Psychological Distress Scores and Percentage With High Psychological Distress, by Study and Over Time

eTable 5. Mean Additional Anxiety Scores and Percentage With Psychological Distress, by Study and Over Time

eTable 6. Mean Prepandemic Psychological Distress Scores by Sociodemographic Characteristics and Study

eTable 7. Meta-analyzed Regression Coefficients: Continuous; Unstratified

eTable 8. Meta-analyzed Regression Coefficients: Continuous; Stratified by Sex

eTable 9. Meta-analyzed Regression Coefficients: – Continuous; Stratified by Age

eTable 10. Meta-analyzed Regression Coefficients: Continuous; Stratified by Education

eTable 11. Meta-analyzed Regression Coefficients: Continuous; Stratified by Education

eTable 12. Meta-analyzed Regression Coefficients: Continuous; Stratified by Country

eTable 13. Meta-analyzed Regression Coefficients: Continuous; Sensitivity; Cohorts With All Time Points

eTable 14. Meta-analyzed Regression Coefficients: Continuous; Sensitivity; Anxiety

eTable 15. Meta-analyzed Regression Coefficients: Continuous; Sensitivity; Depression

eTable 16. Meta-analyzed High Psychological Distress When Named Cohort Is Removed

eTable 17. Meta-analyzed Regression Coefficients: Binary; Unstratified

eTable 18. Meta-analyzed Regression Coefficients: Binary; Stratified by Sex

eTable 19. Meta-analyzed Regression Coefficients: Binary; Stratified by Age

eTable 20. Meta-analyzed Regression Coefficients: Binary; Stratified by Education

eTable 21. Meta-analyzed Regression Coefficients: Binary; Stratified by Ethnicity

eTable 22. Meta-analyzed Regression Coefficients: Binary; Stratified by Country

eTable 23. Meta-analyzed Regression Coefficients: Interactions; Sex

eTable 24. Meta-analyzed Regression Coefficients: Interactions; Ethnicity

eTable 25. Meta-analyzed Regression Coefficients: Interactions; Education

© 2022 Patel K et al. *JAMA Network Open.*

eTable 26. Inclusion and Exclusion Criteria by Cohort for Metaregressions and Sensitivity Analyses

eTable 27. Meta-analyzed Interaction Coefficients

eTable 28. Heterogeneity Explained by Measures Explored by Metaregression Analyses

eAppendix 4. Results of Binary High Psychological Distress Outcomes by Sociodemographic Factor

eAppendix 5. Meta-analyzed High Psychological Distress When One Cohort Is Removed

eAppendix 6. Funding Statements for Each Study

eAppendix 7. Further Study Information

eReferences.

This supplemental material has been provided by the authors to give readers additional information about their work.

eAppendix 1. Ethics and Data Access Statements For Each Study

The most recent sweeps of the Millennium Cohort Study (**MCS**), Next Steps (**NS**), the British Cohort Study 1970 (**BCS70**), the National Child Development Study (**NCDS**) and the National Survey of Health and Development (**NSHD**) and have all been granted ethical approval by the National Health Service (NHS) Research Ethics Committee and all participants have given informed consent. Data for MCS (SN 8682), NS (SN 5545), BCS70 (SN 8547), NCDS (SN 6137) and all four COVID-19 surveys (SN 8658) are available through the UK Data Service. NSHD data are available on request to the NSHD Data Sharing Committee. Interested researchers can apply to access the NSHD data via a standard application procedure. Data requests should be submitted to mrclha.swiftinfo@ucl.ac.uk; further details can be found at http://www.nshd.mrc.ac.uk/data.aspx. doi:10.5522/NSHD/Q101; doi:10.5522/NSHD/Q10.

Ethical approval was obtained from the Avon Longitudinal Study of Parents and Children (**ALSPAC**) Ethics and Law Committee and the Local Research Ethics Committees. The study website contains details of all the data that is available through a fully searchable data dictionary and variable search tool: http://www.bristol.ac.uk/alspac/researchers/our-data. ALSPAC data is available to researchers through an online proposal system. Information regarding access can be found on the ALSPAC website (http://www.bristol.ac.uk/media-library/sites/alspac/documents/researchers/data-access/ALSPAC_Access_Policy.pdf).

The University of Essex Ethics Committee has approved all data collection for the Understanding Society (**USoc**) main study and COVID-19 waves. No additional ethical approval was necessary for this secondary data analysis. All data are available through the UK Data Service (SN 6614 and SN 8644).

Waves 1-9 of the English Longitudinal Study of Aging (**ELSA)** were approved through the National Research Ethics Service, while the COVID-19 Sub-study was approved by the UCL Research Ethics Committee. All participants provided informed consent. All data are available through the UK Data Service (SN 8688 and 5050).

Generation Scotland (**GS**) obtained ethical approval from the East of Scotland Committee on Medical Research Ethics (on behalf of the National Health Service). Reference number 20/ES/0021. Access to data is approved by the Generation Scotland Access Committee. See https://www.ed.ac.uk/generation-scotland/for-researchers/access or email access@generationscotland.org for further details.

All waves of TwinsUK (**TwinsUK**) have received ethical approval associated with TwinsUK Biobank (19/NW/0187), TwinsUK (EC04/015) or Healthy Ageing Twin Study (H.A.T.S) (07/H0802/84) studies from NHS Research Ethics Committees at the Department of Twin Research and Genetic Epidemiology, King's College London. The TwinsUK Resource Executive Committee (TREC) oversees management, data sharing and collaborations involving the TwinsUK registry (for further details see https://twinsuk.ac.uk/resources-for-researchers/access-our-data/).

Born in Bradford (**BiB**) was granted ethical approval by the National Health Service Health Research Authority Yorkshire and the Humber (Bradford Leeds) Research Ethics Committee (references: 16/YH/0320, 15/YH/0455). Data from the various BiB family studies are available to researchers; see the study website for information on how to access data (https://borninbradford.nhs.uk/research/how-to-access-data/).

© 2022 Patel K et al. *JAMA Network Open.*

eAppendix 2. Details of Mental Health Measures in Each Longitudinal Study

The measures used by each study are summarised in S1.3 Supplementary Tables 1 and 2.

MCS: The K-6[1] is a 6-item measure of psychological distress (i.e., general anxiety and depression). Responses are rated on a 5-point Likert-type scale, and capture distress over a period of four weeks prior to administration of the scale. Scores range from 0 to 24, with a conservative cut-off of 13+ applied to indicate probable psychological distress.

ALSPAC: Self-reported depressive symptoms were measured using the short mood and feelings questionnaire (SMFQ).[5] The SMFQ is a 13-item questionnaire that measures the presence of depression symptoms in the previous two weeks and was administered via postal questionnaire or in research clinics. Each item is scored between 0-2, resulting in a summed score between 0-26. Depression severity can be rated in the following score bands: 0-4 none, 5-9 mild, 10-14 moderate, 15-19 moderately severe, 20-27 severe. ALSPAC also used the seven-item Generalized Anxiety Disorder Scale (GAD-7)[11], which is a validated, self-report measure of anxiety used widely by healthcare professionals. Individuals were asked to consider how frequently they have been bothered by a number of problems in the previous two weeks and respond on a four-point Likert scale from 0 "Not at all" to 3 "Nearly every day". The cut-off points for mild, moderate and severe anxiety, are 5, 10, and 15 respectively, and the maximum score is 21.

NS: The 12-item General Health Questionnaire (GHQ)[3] was used to detect symptoms of psychological distress in NS and USoc. The GHQ is a screening instrument designed to detect symptoms of psychological distress (i.e. general anxiety and depression). Each item is scored 0-3 resulting in scores ranging from 0-36. There is an alternative scoring where each item is scored as 0-0-1-1.

BCS70, NCDS: The 9-item version of the Malaise Inventory[2] was used to assess general psychological distress. Items are scored using a simple 'Yes/No' response, meaning continuous scores range from 0-9. Scores of four or more are indicative of probable psychiatric distress.

NSHD: The General Health Questionnaire (GHQ)[3] was also used to detect symptoms of psychological distress in NSHD. For the pre-pandemic sweep, the 28-item GHQ was used, though the 12-item GHQ was used for the subsequent 3 sweeps. A common set of 6 GHQ questions were identified and used in theses analyses. For continuous analyses, each item is scored 0-3 resulting in scores ranging from 0-18. For binary analyses, each item is scored as 0-0-1-1, resulting in scores ranging from 0-6 with a threshold of 2. Sensitivity analyses were conducted to confirm the validity of the 6-item GHQ.

USoc: The 12-item General Health Questionnaire (GHQ)[3] was used, as described for NS

ELSA: Depressive symptoms were measured using an abbreviated 8-item version of the validated Center for Epidemiologic Studies Depression Scale (CES-D).[8] Respondents were asked whether they had experienced any depressive symptoms, such as feeling sad or having restless sleep, in the week prior to interview. For the binary classification, we considered respondents who reported four or more depressive symptoms on the CES-D scale as having elevated depressive symptoms.

© 2022 Patel K et al. *JAMA Network Open.*

GS: Depression and anxiety were assessed during the pandemic sweeps using the GAD-7 and the Patient Health Questionnaire (PHQ-9)[4]. The PHQ-9 is a nine-item, validated tool for the assessment of depressive symptoms experienced in the previous two weeks. Participants are asked to indicate how often they have been bothered by problems such as "Little interest or pleasure in doing things?" on a four-point Likert scale from 0 "Not at all" to 3 "Nearly every day". The score is the sum of the nine items, to a total of 27. A score of 10 or more indicates major depression. The 28-item GHQ[3] was used to assess pre-pandemic psychological distress, and so a comparable composite measure was created from the GAD-7 and the PHQ-9 scales to enable evaluation of change over time. Correlations between each GHQ-28 item and items of the GAD-7 and PHQ-9 scales were examined, and the text of the most highly correlated (r > 0.25) questions was then reviewed and sense-checked; matched items are presented in table S1.2.T1 below. Cut-off scores were determined using ROC curves, which found a general cut-off of >4, a depression cut-off of >5 and an anxiety cut-off of >2. The latter matched the general "proportion" of PHQ-9/GAD-7 scores required for a cut-off.

**Pre-pandemic**				**Pandemic**			
**Measure**	**Subscale**	**Item**	**Question**	**Measure**	**Subscale**	**Item**	**Question**
GHQ-28	Somatic	3	Been feeling run down and out of sorts	PHQ-9	-	4	Feeling tired or having little energy
GHQ-28	Anxiety	2	Had difficulty in staying asleep once you are off	PHQ-9	-	3	Trouble falling or staying asleep, or sleeping too much
GHQ-28	Anxiety	4	Been getting edgy and bad-tempered	GAD-7	-	6	Becoming easily annoyed or irritable
GHQ-28	Anxiety	5	Been getting scared or panicky for no good reason	GAD-7	-	1	Feeling nervous, anxious or on edge
GHQ-28	Depression	1	Been thinking of yourself as a worthless person	PHQ-9	-	6	Feeling bad about yourself - or that you are a failure or have let yourself or your family down
GHQ-28	Depression	4	Thought of the possibility that you might make away with yourself	PHQ-9	-	9	Thoughts that you would be better off dead or of hurting yourself in some way
GHQ-28	Depression	5	Found at times you couldn't do anything because your nerves were too bad	PHQ-9	-	7	Trouble concentrating on things, such as reading the newspaper or watching television

TwinsUK: The Hospital Anxiety and Depression Scale (HADS)[9] is a 14-item scale used to measure levels of psychiatric distress in non-psychiatric patient populations. Responses are indicated on a 4-point ordinal Likert scale.

BiB: Self-reported depressive symptoms were measured using the PHQ-8[10]. The PHQ-8 is an eight-item, validated tool for the assessment of depressive symptoms experienced in the previous two weeks. Participants are asked to indicate how often they have been bothered by problems such as "Little interest or pleasure in doing things?" on a four-point Likert scale from 0 "Not at all" to 3 "Nearly every day". The score is the sum of the eight items, to a total of 24. A score of 10 or more indicates major depression. The pre-pandemic measure was administered in a clinic setting and subsequent data collection waves were completed by post. The GAD-7 anxiety measure is also used, as described for ALSPAC.

© 2022 Patel K et al. *JAMA Network Open.*

eAppendix 3. Participant Flow Diagrams

MCS

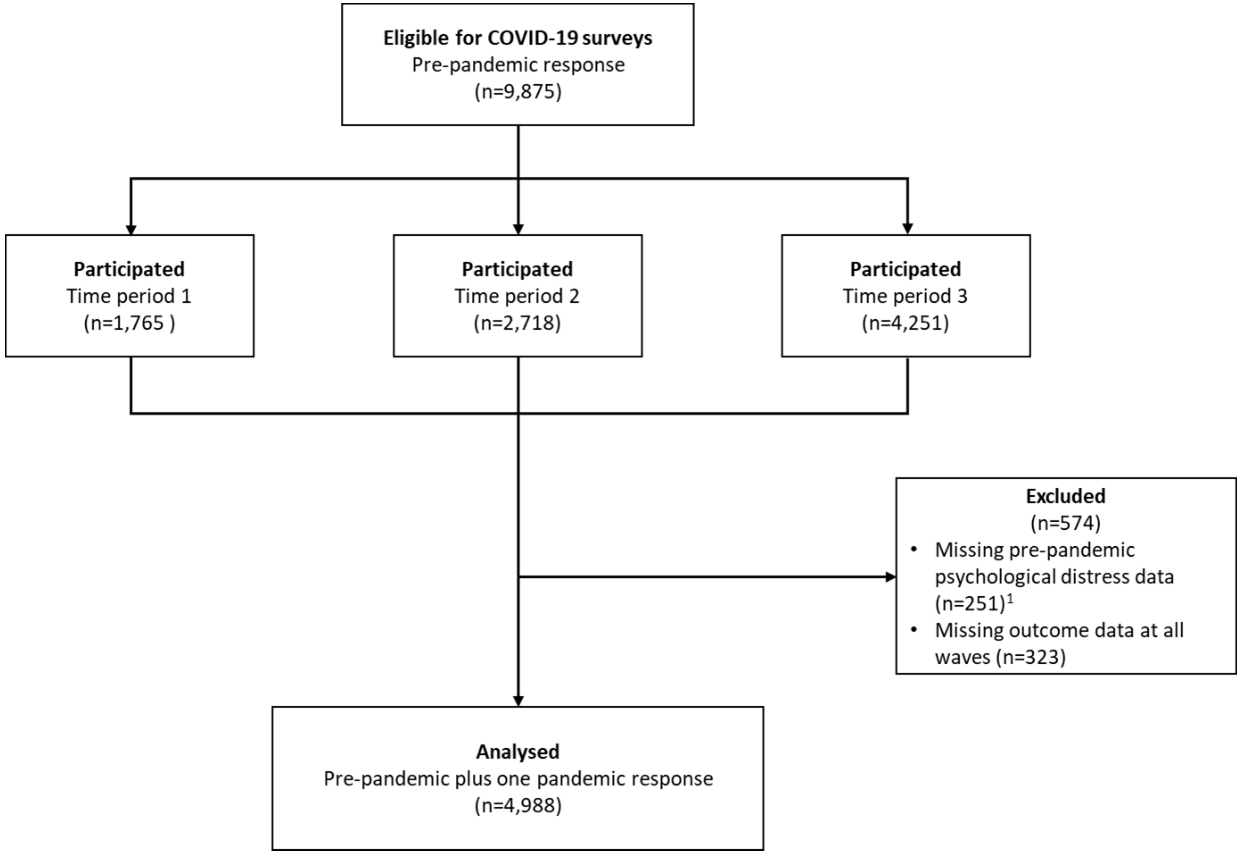

© 2022 Patel K et al. *JAMA Network Open.*

ALSPAC

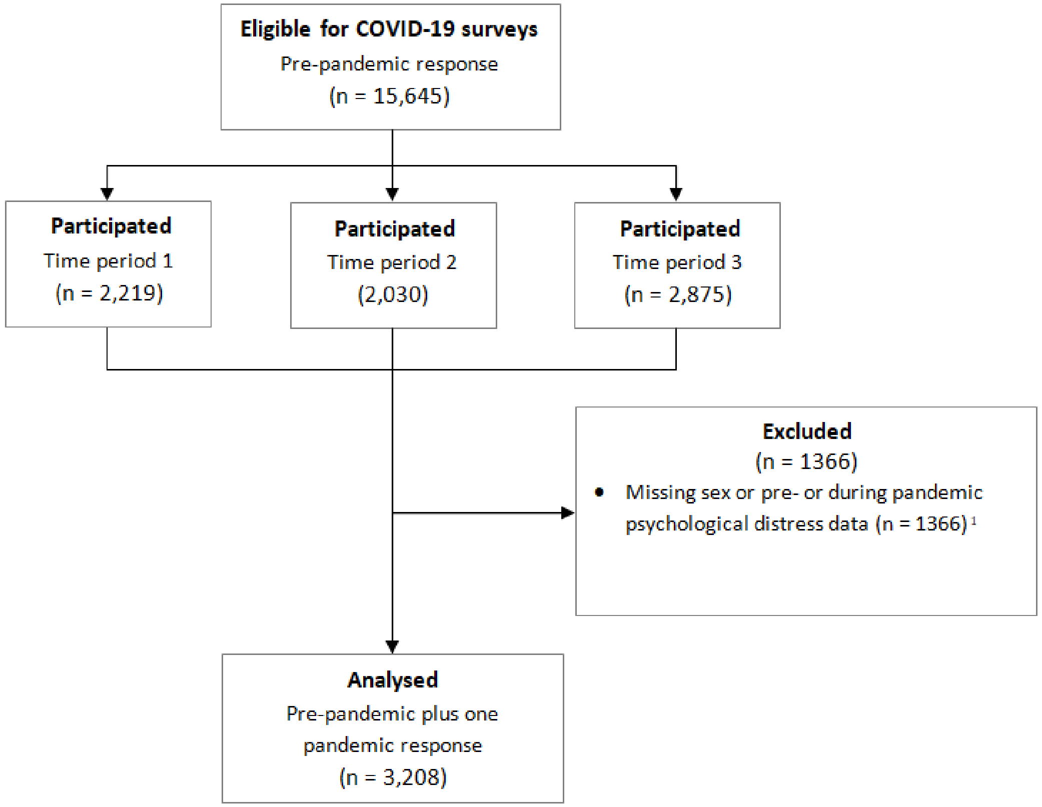

© 2022 Patel K et al. *JAMA Network Open.*

NS

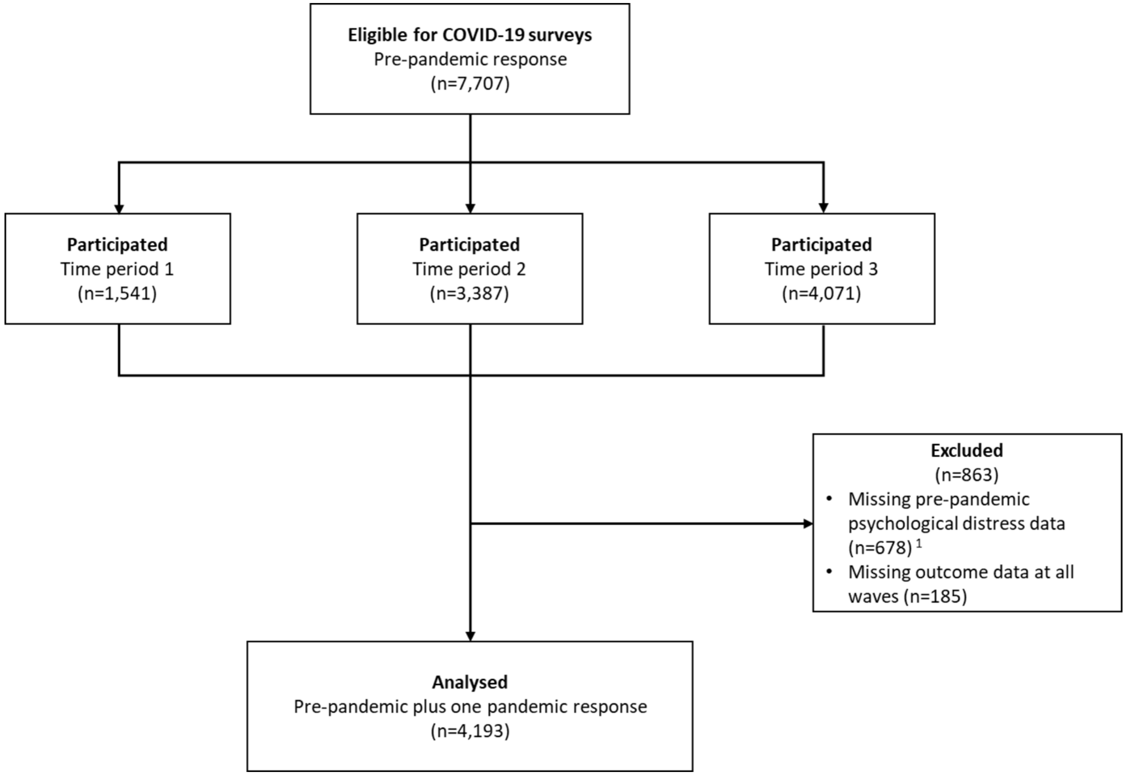

© 2022 Patel K et al. *JAMA Network Open.*

BCS70

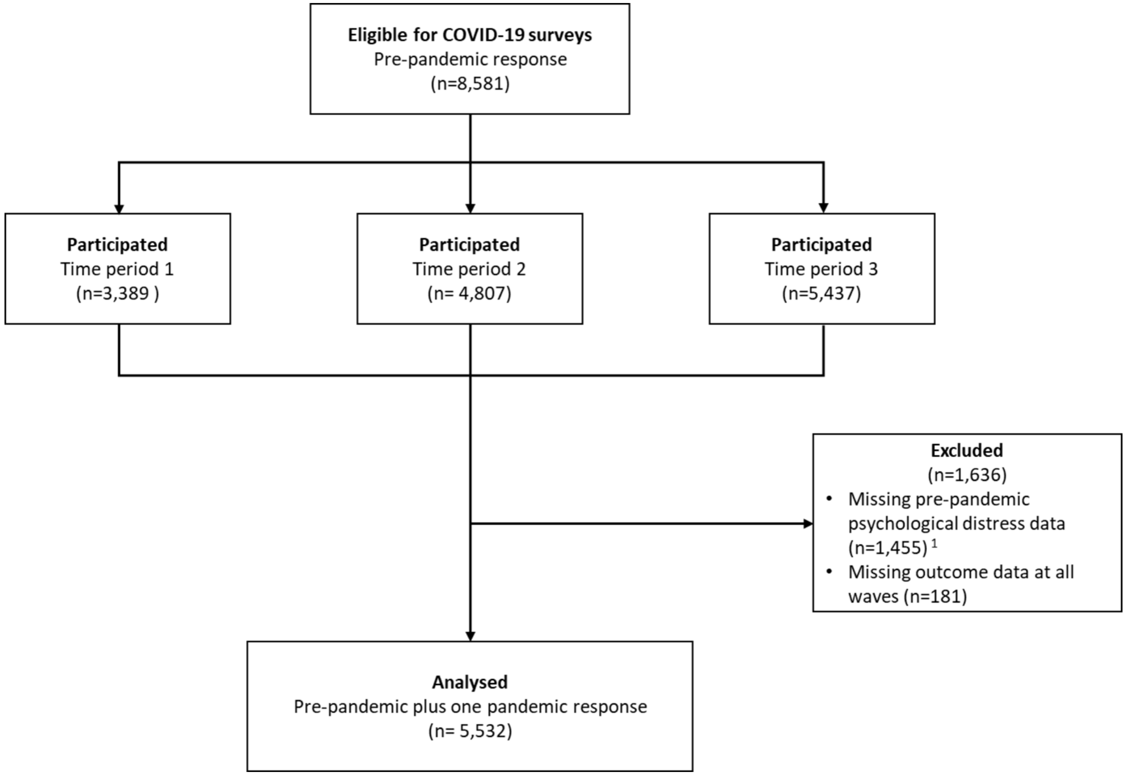

© 2022 Patel K et al. *JAMA Network Open.*

NCDS

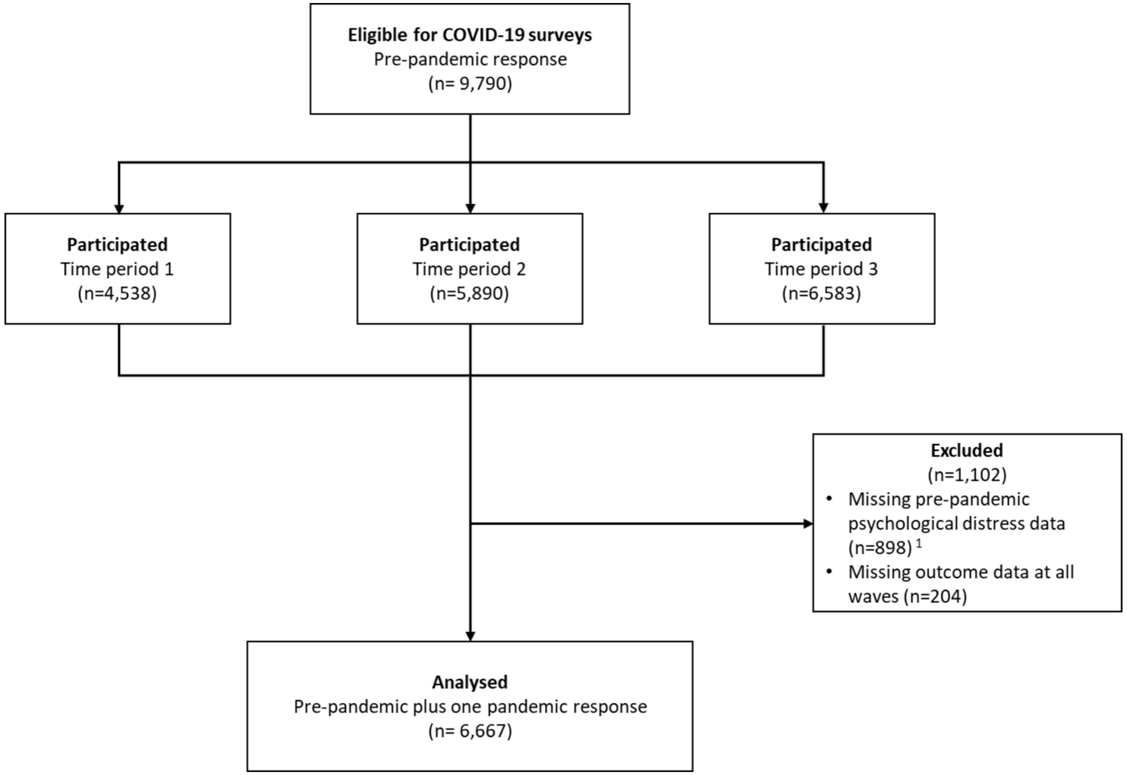

© 2022 Patel K et al. *JAMA Network Open.*

NSHD

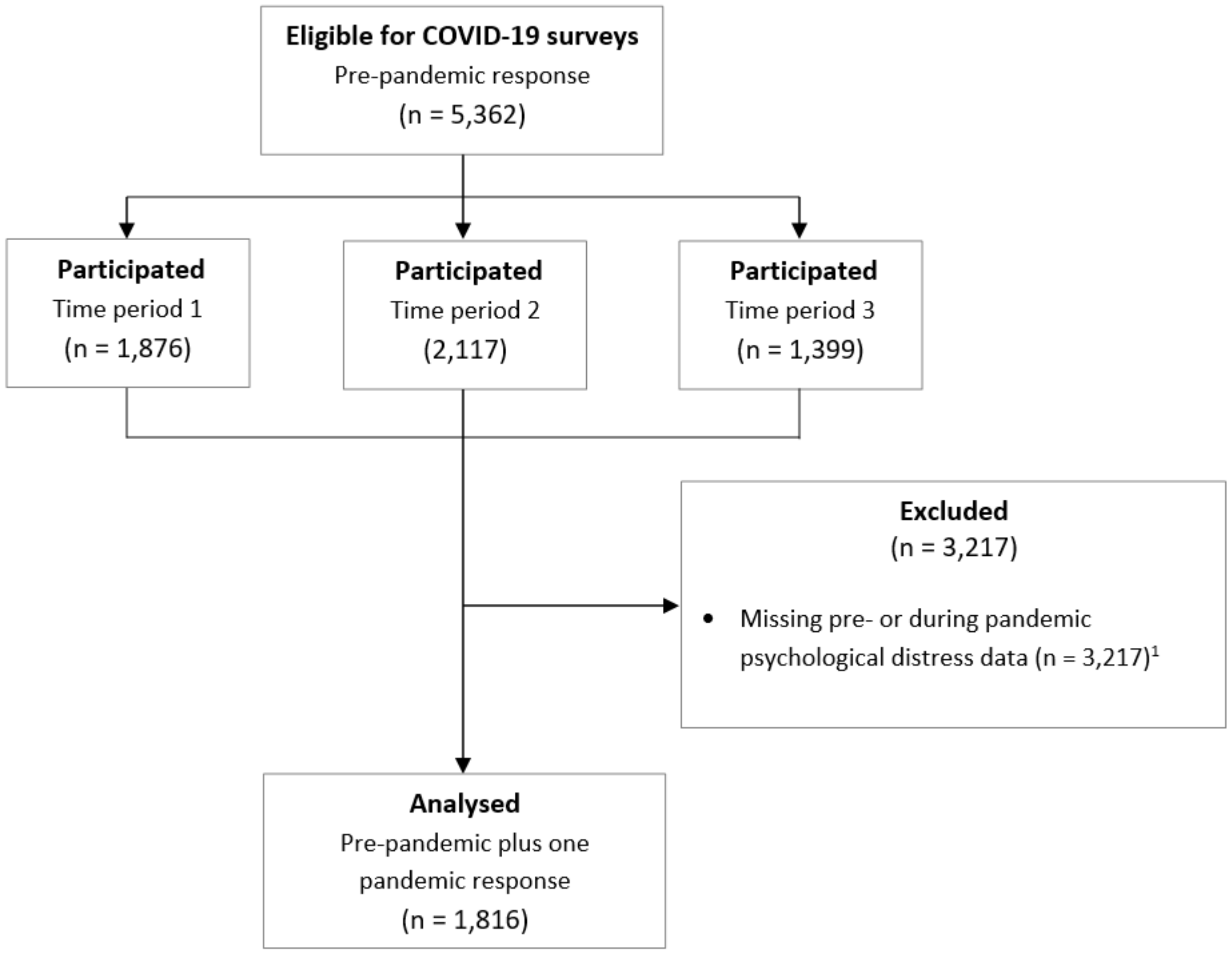

© 2022 Patel K et al. *JAMA Network Open.*

USoc

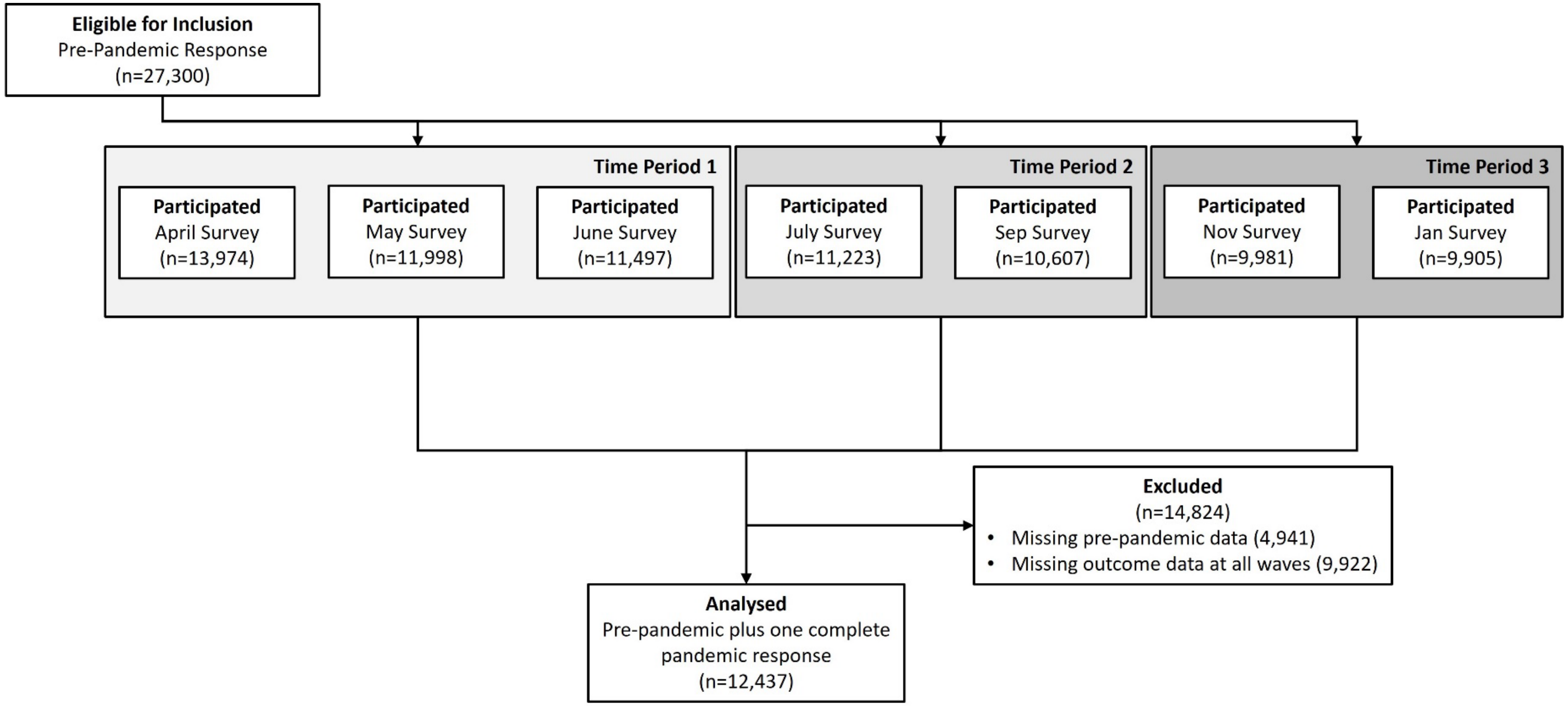

Note: N participated at each wave excludes those without valid survey weights (n=2,054-3,787).

© 2022 Patel K et al. *JAMA Network Open.*

ELSA

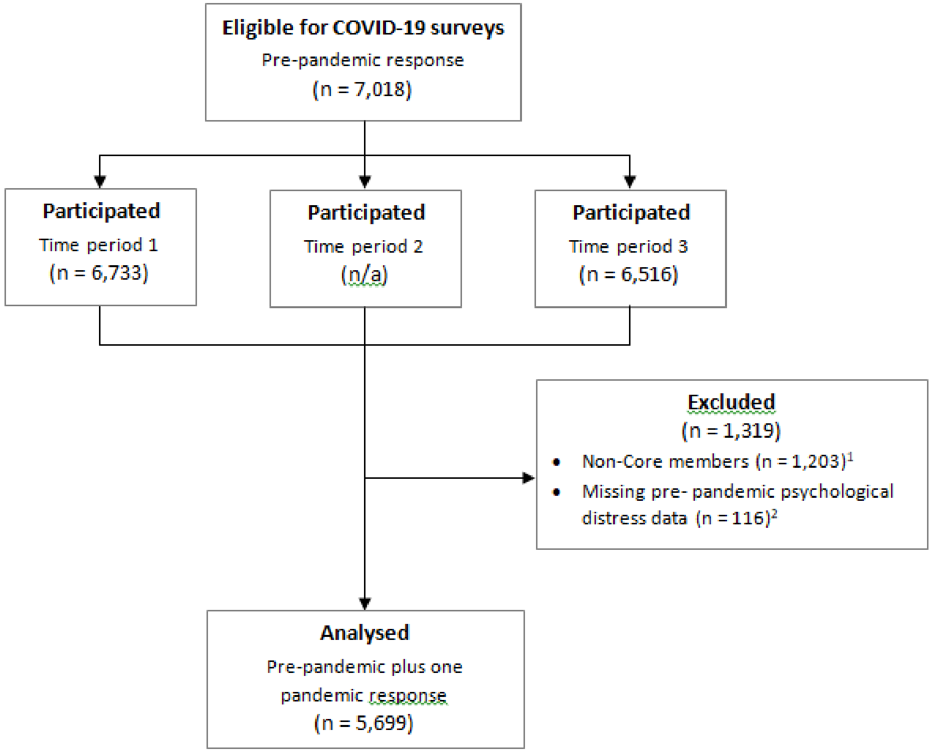

© 2022 Patel K et al. *JAMA Network Open.*

GS

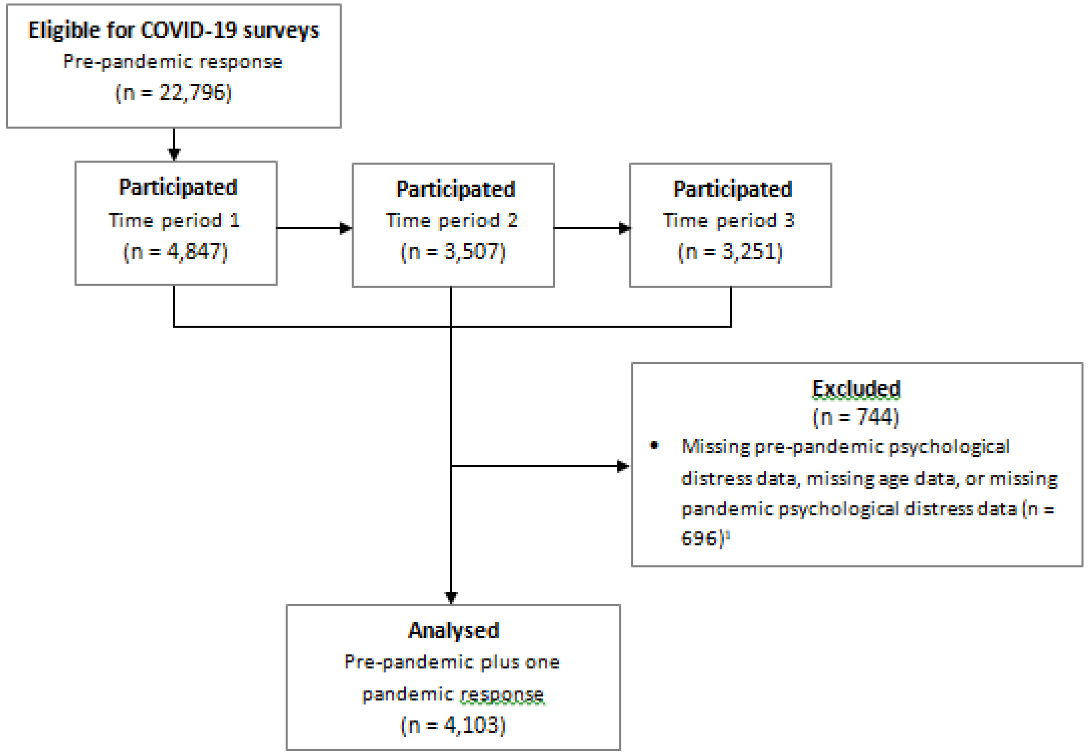

© 2022 Patel K et al. *JAMA Network Open.*

TwinsUK

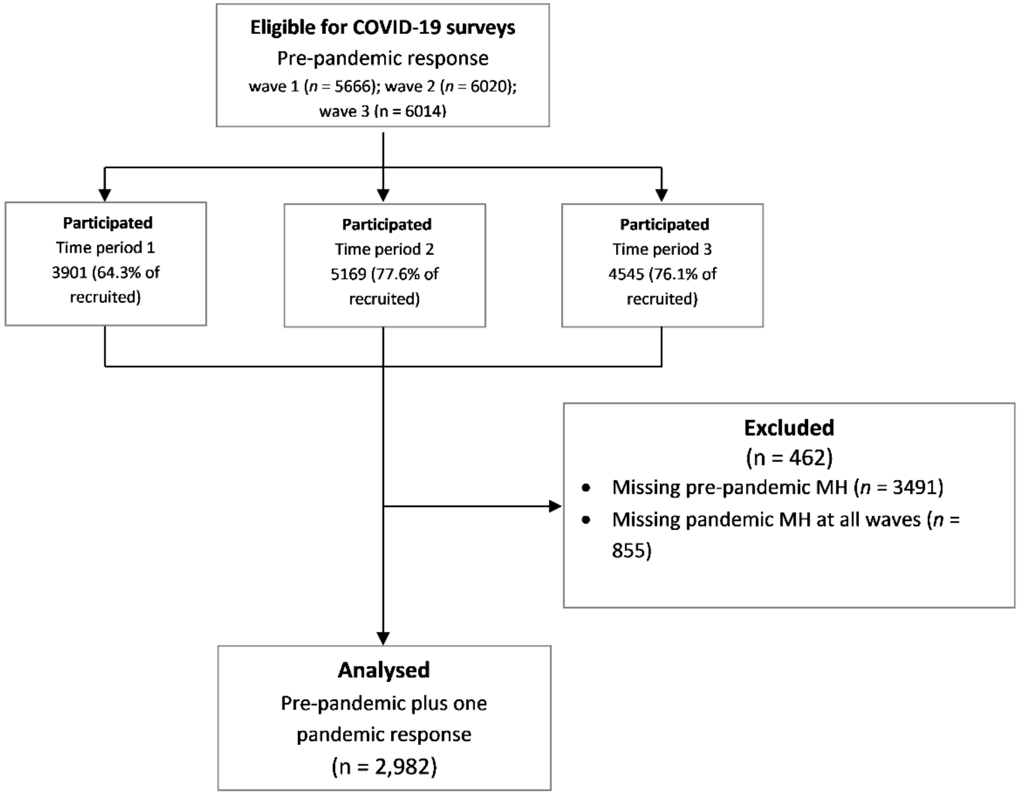

© 2022 Patel K et al. *JAMA Network Open.*

BiB

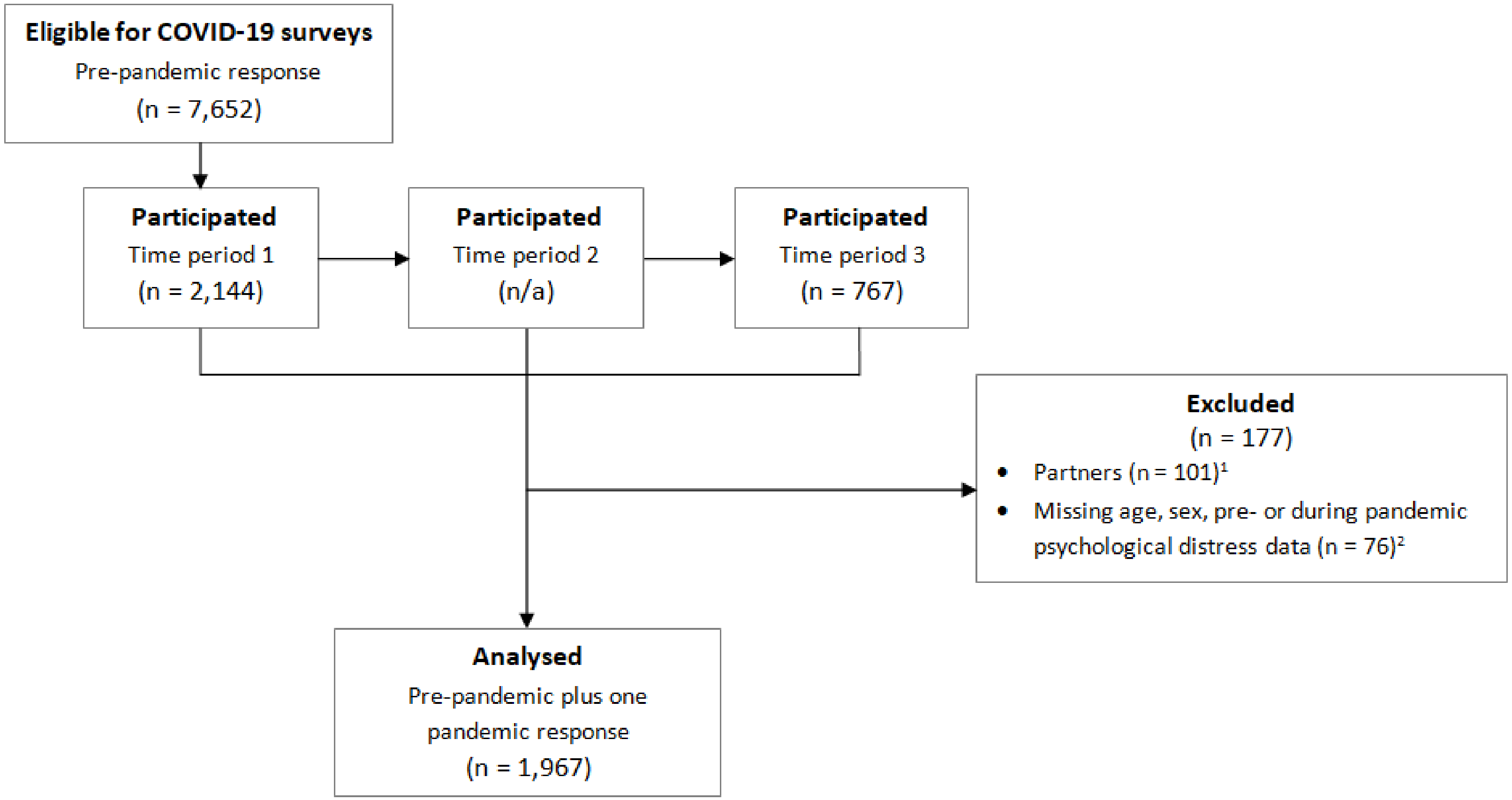

[2] Decision to exclude from all contributing studies

© 2022 Patel K et al. *JAMA Network Open.*

eTable 1. Measures of General Psychological Distress in Each Study

	MCS	ALSPAC	NS	BCS 70	NCDS	NSHD	USoc	ELSA	GS	TwinsUK	BiB
Measure	K-6	SMFQ	GHQ-12	Malaise Inventory	Malaise Inventory	GHQ-6	GHQ-12	CES-D-8	Composite*	HADS	PHQ-8
Number of items	6	13	12	9	9	6	12	8	7	14	8
Possible range	0-24	0-26	0-36	0-9	0-9	0-18	0-36	0-70	0-21	0-36	0-24
Threshold for high psychological distress	13+	11+	4+	4+	4+	2+	4+	24+	5+	11+	10+

*see eAppendix 2.

© 2022 Patel K et al. *JAMA Network Open.*

eTable 2. Additional Measures of Anxiety by Study

Study	ALSPAC	GS	BiB
**Measure**	**GAD-7**	**Composite***	**GAD-7**
Number of items	7	2	7
Possible range	0-21	0-6	0-21
Threshold for high psychological distress	10+	3+	10+

© 2022 Patel K et al. *JAMA Network Open.*

eTable 3. Distribution of Participant Characteristics by Study

Characteristics (n (%))	MCS	ALSPAC	NS	BCS 70	NCDS	NSHD	USoc	ELSA	GS	TwinsUK	BiB
Analytic sample	4988	3208	3579	5532	6667	1816	12437	5699	4103	2981	1967
**Gender**											
Men	1956 (39.2)	986 (30.8)	1676 (37.4)	2420 (43.8)	3145 (47.2)	853 (47.0)	5229 (47.9)	2440 (47.0)	1539 (37.5)	306 (10.3)	- -
Women	3032 (60.8)	2219 (69.2)	2219 (62.5)	3112 (56.3)	3522 (52.8)	963 (53.0)	7208 (52.1)	3259 (53.0)	2565 (62.5)	2675 (89.7)	1967 (100.0)
**Age group**											
16-24	4988 (100.0)	- -	- -	- -	- -	- -	972 (12.7)	- -	- -	10 (0.3)	50 (2.5)
25-34	- -	3208 (100.0)	4193 (100.0)	- -	- -	- -	1278 (12.8)	- -	182 (4.4)	89 (3.0)	556 (28.2)
35-44	- -	- -	- -	- -	- -	- -	1905 (13.8)	- -	373 (9.1)	209 (7.0)	1006 (51.1)
45-54	- -	- -	- -	5532 (100.0)	- -	- -	2574 (17.2)	642 (18.4)	739 (18.0)	323 (10.8)	355** (18.0)
55-64	- -	- -	- -	- -	6667 (100.0)	- -	2641 (17.2)	1210 (32.7)	1151 (28.1)	654 (21.6)	- -
65-74	- -	- -	- -	- -	- -	1816 (100.0)	2210 (14.7)	2342 (30.1)	1393 (33.9)	1126 (37.8)	- -
75+	- -	- -	- -	- -	- -	- -	857 (11.6)	1505 (18.8)	265 (6.5)	579 (19.4)	- -
**Education**											
Degree	2249 (45.1)	1767 (64.7)	2121 (50.6)	2983 (53.9)	3576 (56.1)	212 (12.3)	5917 (37.0)	1407 (23.0)	1887 (46.0)	1525 (51.2)	542 (27.6)
Non-Degree	2736 (54.9)	962 (35.3)	2072 (49.4)	2548 (46.1)	2796 (43.9)	1510 (87.7)	6520 (63.0)	4289 (77.0)	2132 (52.0)	1368 (45.9)	1013 (51.5)
**Ethnicity**											
White	4161 (83.8)	2822 (96.3)	3073 (74.6)	- -	- -	1816 (100.0)	10966 (92.5)	5467 (93.5)	3994 (96.3)	2927 (98.2)	694 (35.3)
Non-White	804 (16.2)	108 (3.7)	1049 (25.4)	- -	- -	- -	1471 (7.5)	232 (6.5)	26 (0.6)	47 (1.6)	1223 (62.2)
**Country of residence**											
England	3165 (82.9)	3208 (100.0)	3965 (96.8)	4544 (85.9)	5453 (84.0)	1517 (87.6)	10442 (84.8)	5699 (100.0)	- -	2733 (91.6)	1967 (100.0)
Scotland	578 (9.3)	- -	24 (0.6)	426 (8.1)	578 (8.9)	138 (8.0)	942 (8.0)	- -	4103 (100.0)	121 (4.1)	- -
Wales	567 (5.1)	- -	28 (0.7)	273 (5.2)	351 (5.4)	77 (4.5)	634 (4.6)	- -	- -	108 (3.6)	- -
Northern Ireland	376 (8.0)	- -	5 (0.1)	2 (0.1)	5 (0.1)	- -	419 (2.6)	- -	- -	2 (0.1)	- -

Notes: Samples for each study restricted to respondents with non-missing pre-pandemic psychological distress measure, with at least one response during the pandemic, and valid information on sex and age. Percentages are calculated from available data within strata. Information not available/applicable: - ; suppressed small number: *, categories collapsed e.g. Age group > 44: **

© 2022 Patel K et al. *JAMA Network Open.*

eTable 4. Mean Psychological Distress Scores and Percentage With High Psychological Distress, by Study and Over Time

	MCS	ALSPAC	NS	BCS 70	NCDS	NSHD	USoc	ELSA	GS	TwinsUK	BiB
Measure	K-6	SMFQ	GHQ-12	Malaise Inventory	Malaise Inventory	GHQ-6	GHQ-12	CES-D-8	Composite*	HADS	PHQ-8
**Mean score (SD)**											
Pre-pandemic	7.8 (4.9)	6.8 (6.4)	11.8 (6.3)	1.9 (2.2)	1.4 (1.9)	4.3 (1.9)	11.3 (5.5)	1.3 (1.8)	15.1 (7.9)	7.8 (6.2)	4.1 (4.7)
Wave 1	8.0 (5.1)	6.2 (5.6)	13.5 (6.0)	1.8 (2.0)	1.4 (1.8)	5.7 (2.8)	12.5 (6.1)	2.0 (2.1)	25.4 (4.9)	8.6 (6.5)	5.2 (5.4)
Wave 2	8.5 (5.5)	6.5 (5.7)	13.2 (5.7)	2.1 (2.2)	1.6 (2.0)	5.4 (2.8)	11.9 (5.7)	- -	25.8 (4.8)	10.2 (5.5)	- -
Wave 3	9.8 (5.9)	6.6 (6.0)	13.4 (5.9)	1.9 (2.1)	1.5 (1.9)	4.7 (1.9)	12.9 (6.2)	2.3 (2.4)	24.8 (4.7)	10.5 (5.8)	5.0 (5.3)
**High psychological distress (%)**											
Pre-pandemic	18.9	23.9	26.0	21.9	14.3	11.4	19.6	11.5	11.7	6.8	11.6
Wave 1	19.0	17.8	35.5	18.0	12.2	35.0	27.3	22.0	5.4	7.4	18.9
Wave 2	24.8	21.3	30.5	22.7	15.9	29.1	20.8	-	5.1	6.9	-
Wave 3	33.5	21.6	34.4	21.1	14.8	16.6	27.0	28.0	6.5	7.8	16.8

*see eAppendix 2

© 2022 Patel K et al. *JAMA Network Open.*

eTable 5. Mean Additional Anxiety Scores and Percentage With Psychological Distress, by Study and Over Time

	ALSPAC		GS		BiB	
Measure	GAD-7		Composite*		GAD-7	
**Mean score (SD)**						
Pre-pandemic	6.8	(6.4)	0.9	(1.0)	3.2	(4.4)
Wave 1	6.2	(5.6)	3.5	(4.3)	4.6	(5.2)
Wave 2	6.5	(5.7)	2.8	(4.0)	-	-
Wave 3	6.6	(6.0)	3.8	(4.5)	4.3	(5.0)
**High psychological distress (%)**						
Pre-pandemic	23.9		6.6		10.1	
Wave 1	17.8		10.3		15.7	
Wave 2	21.3		7.3		-	
Wave 3	21.6		10.9		13.4	

*see S1.2

© 2022 Patel K et al. *JAMA Network Open.*

eTable 6. Mean Prepandemic Psychological Distress Scores by Sociodemographic Characteristics and Study

**Characteristics** (Mean (95% CI))	MCS	ALSPAC	NS	BCS 70	NCDS	NSHD	USoc	ELSA	GS	TwinsUK	BiB
**Overall**	7.8 (7.6-8.1)	6.8 (6.6-7.0)	11.8 (11.4-12.2)	1.9 (1.7-2.0)	1.4 (1.3-1.5)	4.3 (4.2-4.4)	11.3 (11.2-11.5)	1.3 (1.3-1.4)	15.1 (14.9-15.3)	7.8 (7.6-8.0)	4.1 (3.9-4.3)
**Gender**											
Men	6.7 (6.3-7.1)	5.2 (4.9-5.6)	11.1 (10.5-11.8)	1.5 (1.3-1.7)	1.1 (1.0-1.2)	4.0 (3.9-4.1)	10.6 (10.4-10.8)	1.0 (1.0-1.1)	14.1 (13.7-14.5)	6.8 (6.2-7.3)	-
Women	8.5 (8.2-8.8)	7.5 (7.2-7.8)	12.4 (11.9-13.0)	2.2 (2.0-2.4)	1.8 (1.6-1.9)	4.4 (4.4-4.7)	12.0 (11.9-12.2)	1.6 (1.5-1.6)	15.8 (15.5-16.1)	7.9 (7.7-8.1)	4.1 (3.9-4.3)
**Age group**											
16-24	7.8 (7.6-8.1)	-	-	-	-	-	12.4 (12.0-12.9)	-	-	11.6 (7.5-15.7)	6.2 (4.6-7.8)
25-34	-	6.8 (6.6-7.0)	11.8 (11.4-12.2)	-	-	-	12.2 (11.7-12.7)	-	15.6 (14.8-16.4)	9.6 (8.4-10.8)	4.6 (4.2-5.0)
35-44	-	-	-	-	-	-	11.9 (11.6-12.3)	-	15.6 (15.1-16.2)	8.9 (8.0-9.7)	3.8 (3.5-4.1)
45-54	-	-	-	1.9 (1.7-2.0)	-	-	11.8 (11.5-12.0)	1.3 (1.2-1.5)	16.17 (15.7-16.7)	8.5 (07.9-9.2	3.8** (3.3-4.2)
55-64	-	-	-	-	1.4 (1.3-1.5)	-	11.3 (11.1-11.6)	1.4 (1.3-1.5)	14.3 (14.0-14.7)	8.0 (7.6-8.5)	-
65-74	-	-	-	-	-	4.3 (4.2-4.4)	9.9 (9.7-10.1)	1.2 (1.1-1.3)	12.4 (11.6-13.2)	7.1 (6.8-7.4)	-
75+	-	-	-	-	-		9.8 (9.5-10.1)	1.4 (1.3-1.5)	15.4 (8.9-22.0)	7.6 (7.2-7.9)	-
**Education**											
Degree	7.7 (7.4-8.0)	6.2 (5.9-6.5)	11.5 (11.0-12.0)	1.6 (1.5-1.7)	1.2 (1.1-1.3)	4.0 (3.8-4.2)	11.2 (11.1-11.4)	1.0 (0.9-1.1)	14.9 (14.5-15.2)	7.9 (7.6-8.2)	3.5 (3.2-3.9)
Non-Degree	8.0 (7.6-8.4)	7.7 (7.3-8.2)	12.1 (11.5-12.7)	2.0 (1.8-2.2)	1.6 (1.4-1.7)	4.3 (4.2-4.4)	11.4 (11.2-11.6)	1.4 (1.3-1.5)	15.3 (14.9-15.6)	7.4 (7.1-7.7)	4.4 (4.1-4.7)

© 2022 Patel K et al. *JAMA Network Open.*

Characteristics (Mean (95% CI))	MCS	ALSPAC	NS	BCS 70	NCDS	NSHD	USoc	ELSA	GS	TwinsUK	BiB
**Ethnicity**											
White	7.9 (7.6-8.1)	6.8 (6.6-7.0)	11.7 (11.2-12.2)	-	-	4.3 (4.2-4.4)	11.3 (11.2-11.4)	1.3 (1.2-1.3)	15.1 (14.9-15.4)	9.2 (7.4-10.9)	4.3 (3.9-4.6)
Non-White	7.4 (6.7-8.0)	8.0 (6.5-9.4)	12.0 (11.2-12.8)	-	-	-	11.8 (11.3-12.4)	2.0 (1.6-2.3)	16.6 (12.21.0)	7.7 (7.5-7.9)	4.1 (3.8-4.4)
**Country of residence**											
England	7.7 (7.4-8.0)	6.8 (6.6-7.0)	11.8 (11.4-12.3)	1.9 (1.7-2.0)	1.4 (1.3-1.5)	4.3 (4.2-4.4)	11.3 (11.2-11.5)	1.3 (1.3-1.4)	- -	7.8 (7.6-8.0)	4.1 (3.9-4.3)
Scotland	8.2 (7.6-8.8)	-	11.3 (7.1-15.6)	2.0 (1.6-2.4)	1.3 (0.9-1.7)	4.1 (3.9-4.4)	11.6 (11.1-12.0)	-	15.1 (14.9-15.3)	7.6 (6.5-8.7)	-
Wales	8.0 (7.4-8.6)	-	9.7 (8.0-11.4)	1.7 (1.3-2.0)	1.5 (1.1-1.9)	4.2 (3.7-4.7)	11.6 (11.1-12.2)	-	-	7.7 (6.8-8.7)	-
Northern Ireland	8.2 (7.3-9.0)	-	12.5 (-6.6-31.6)	-	-	-	-	-	-	-	-

© 2022 Patel K et al. *JAMA Network Open.*

eTable 7. Meta-analyzed Regression Coefficients: Continuous; Unstratified

Timepoint	Study	Strata	SMD	lower_ci	upper_ci	%Weight	%I2
1 vs 0	ALSPAC	Overall	-0.08	-0.11	-0.05	9.2	
1 vs 0	BCS70	Overall	-0.03	-0.07	0.02	9.2	
1 vs 0	BiB	Overall	0.22	0.17	0.26	9.1	
1 vs 0	ELSA	Overall	0.25	0.22	0.29	9.2	
1 vs 0	GS	Overall	0.29	0.26	0.32	9.2	
1 vs 0	MCS	Overall	0.09	0.05	0.14	9.1	
1 vs 0	NCDS	Overall	-0.04	-0.07	-0.01	9.2	
1 vs 0	NS	Overall	0.28	0.19	0.38	8.6	
1 vs 0	NSHD	Overall	0.46	0.37	0.55	8.6	
1 vs 0	TwinsUK	Overall	0.21	0.19	0.24	9.2	
1 vs 0	USoc	Overall	0.07	0.06	0.07	9.3	
**1 vs 0**	**Overall**	**Overall**	**0.15**	**0.06**	**0.25**		**99.2**
2 vs 0	BCS70	Overall	0.09	0.05	0.14	13.2	
2 vs 0	GS	Overall	0.24	0.21	0.27	13.4	
2 vs 0	MCS	Overall	0.16	0.10	0.21	13.0	
2 vs 0	NCDS	Overall	0.08	0.05	0.11	13.4	
2 vs 0	NS	Overall	0.21	0.14	0.27	12.8	
2 vs 0	NSHD	Overall	0.16	-0.08	0.40	6.9	
2 vs 0	TwinsUK	Overall	0.42	0.40	0.45	13.5	
2 vs 0	USoc	Overall	0.04	0.03	0.04	13.7	
**2 vs 0**	**Overall**	**Overall**	**0.18**	**0.09**	**0.27**		**98.6**
3 vs 0	ALSPAC	Overall	-0.04	-0.08	-0.01	9.2	
3 vs 0	BCS	Overall	0.02	-0.03	0.07	9.1	
3 vs 0	BiB	Overall	0.23	0.16	0.29	9.0	
3 vs 0	ELSA	Overall	0.43	0.39	0.46	9.2	
3 vs 0	GS	Overall	0.47	0.43	0.50	9.2	
3 vs 0	MCS	Overall	0.19	0.13	0.24	9.1	
3 vs 0	NCDS	Overall	0.05	0.00	0.09	9.2	
3 vs 0	NS	Overall	0.25	0.19	0.32	9.0	
3 vs 0	NSHD	Overall	0.10	-0.02	0.21	8.4	
3 vs 0	TwinsUK	Overall	0.47	0.45	0.50	9.3	
3 vs 0	USoc	Overall	0.09	0.08	0.10	9.3	
**3 vs 0**	**Overall**	**Overall**	**0.21**	**0.10**	**0.32**		**99.2**

© 2022 Patel K et al. *JAMA Network Open.*

eTable 8. Meta-analyzed Regression Coefficients: Continuous; Stratified by Sex

Timepoint	Study	Strata	SMD	lower_ci	upper_ci	%Weight	%I2
1 vs 0	MCS	female	0.14	0.08	0.20	9.1	
1 vs 0	ALSPAC	female	-0.08	-0.13	-0.04	9.3	
1 vs 0	NS	female	0.36	0.25	0.47	8.6	
1 vs 0	BCS70	female	0.03	-0.03	0.08	9.1	
1 vs 0	NCDS	female	-0.06	-0.11	-0.01	9.2	
1 vs 0	NSHD	female	0.47	0.34	0.60	8.3	
1 vs 0	USoc	female	0.08	0.07	0.09	9.4	
1 vs 0	ELSA	female	0.27	0.23	0.32	9.2	
1 vs 0	GS	female	0.37	0.33	0.41	9.3	
1 vs 0	TwinsUK	female	0.22	0.20	0.25	9.3	
1 vs 0	BiB	female	-0.02	-0.07	0.03	9.2	
**1 vs 0**	**Overall**	**female**	**0.16**	**0.05**	**0.27**		**99.0**
1 vs 0	MCS	male	0.05	-0.04	0.14	9.8	
1 vs 0	ALSPAC	male	-0.07	-0.12	-0.02	10.4	
1 vs 0	NS	male	0.16	0.00	0.33	8.1	
1 vs 0	BCS70	male	-0.08	-0.14	-0.03	10.4	
1 vs 0	NCDS	male	-0.02	-0.06	0.01	10.6	
1 vs 0	NSHD	male	0.45	0.32	0.58	8.9	
1 vs 0	USoc	male	0.05	0.04	0.06	10.8	
1 vs 0	ELSA	male	0.23	0.18	0.28	10.4	
1 vs 0	GS	male	0.15	0.11	0.20	10.5	
1 vs 0	TwinsUK	male	0.12	0.04	0.19	10.1	
**1 vs 0**	**Overall**	**male**	**0.10**	**0.00**	**0.19**		**97.8**
2 vs 0	MCS	female	0.19	0.11	0.27	13.2	
2 vs 0	NS	female	0.21	0.13	0.30	13.0	
2 vs 0	BCS70	female	0.17	0.11	0.23	13.8	
2 vs 0	NCDS	female	0.11	0.07	0.16	14.0	
2 vs 0	NSHD	female	0.16	-0.29	0.61	3.4	
2 vs 0	USoc	female	0.04	0.03	0.05	14.5	
2 vs 0	GS	female	0.30	0.26	0.35	14.0	
2 vs 0	TwinsUK	female	0.43	0.40	0.45	14.3	
**2 vs 0**	**Overall**	**female**	**0.21**	**0.11**	**0.30**		**97.8**
2 vs 0	MCS	male	0.14	0.05	0.22	12.6	
2 vs 0	NS	male	0.20	0.11	0.29	12.3	
2 vs 0	BCS70	male	0.01	-0.06	0.08	13.0	
2 vs 0	NCDS	male	0.05	0.00	0.10	13.7	
2 vs 0	NSHD	male	0.16	-0.06	0.38	7.6	
2 vs 0	USoc	male	0.03	0.02	0.04	14.2	
2 vs 0	GS	male	0.13	0.08	0.18	13.6	
2 vs 0	TwinsUK	male	0.39	0.32	0.46	13.0	

© 2022 Patel K et al. *JAMA Network Open.*

**2 vs 0**	**Overall**	**male**	**0.14**	**0.05**	**0.22**		**95.7**
3 vs 0	MCS	female	0.26	0.18	0.34	9.1	
3 vs 0	ALSPAC	female	-0.06	-0.11	-0.02	9.3	
3 vs 0	NS	female	0.27	0.19	0.36	9.0	
3 vs 0	BCS70	female	0.06	-0.01	0.13	9.2	
3 vs 0	NCDS	female	0.07	-0.01	0.14	9.1	
3 vs 0	NSHD	female	0.11	-0.07	0.28	7.9	
3 vs 0	USoc	female	0.10	0.09	0.11	9.4	
3 vs 0	ELSA	female	0.47	0.42	0.52	9.3	
3 vs 0	GS	female	0.56	0.51	0.60	9.3	
3 vs 0	TwinsUK	female	0.48	0.45	0.51	9.4	
3 vs 0	BiB	female	0.22	0.14	0.31	9.0	
**3 vs 0**	**Overall**	**female**	**0.23**	**0.11**	**0.35**		**99.0**
3 vs 0	MCS	male	0.12	0.04	0.20	9.9	
3 vs 0	ALSPAC	male	0.00	-0.06	0.05	10.3	
3 vs 0	NS	male	0.24	0.14	0.34	9.6	
3 vs 0	BCS70	male	-0.02	-0.09	0.05	10.1	
3 vs 0	NCDS	male	0.03	-0.02	0.08	10.4	
3 vs 0	NSHD	male	0.09	-0.06	0.23	8.6	
3 vs 0	USoc	male	0.08	0.07	0.09	10.6	
3 vs 0	ELSA	male	0.38	0.32	0.43	10.3	
3 vs 0	GS	male	0.31	0.26	0.36	10.3	
3 vs 0	TwinsUK	male	0.40	0.33	0.47	10.0	
**3 vs 0**	**Overall**	**male**	**0.16**	**0.06**	**0.26**		**97.5**

© 2022 Patel K et al. *JAMA Network Open.*

eTable 9. Meta-analyzed Regression Coefficients: – Continuous; Stratified by Age

Timepoint	Study	Strata	SMD	lower_ci	upper_ci	%Weight	%I2
1 vs 0	MCS	16-24	0.09	0.05	0.14	20.6	
1 vs 0	USoc	16-24	0.10	0.08	0.13	78.7	
1 vs 0	TwinsUK	16-24	0.34	-0.05	0.73	0.3	
1 vs 0	BiB	16-24	-0.15	-0.46	0.17	0.5	
**1 vs 0**	**Overall**	**16-24**	**0.10**	**0.08**	**0.12**		**0.0**
1 vs 0	ALSPAC	25-34	-0.08	-0.11	-0.05	17.1	
1 vs 0	NS	25-34	0.28	0.19	0.38	16.8	
1 vs 0	USoc	25-34	0.11	0.09	0.13	17.1	
1 vs 0	GS	25-34	0.93	0.76	1.10	16.1	
1 vs 0	TwinsUK	25-34	0.43	0.27	0.59	16.2	
1 vs 0	BiB	25-34	0.14	0.04	0.23	16.8	
**1 vs 0**	**Overall**	**25-34**	**0.29**	**0.02**	**0.57**		**99.4**
1 vs 0	USoc	35-44	0.08	0.06	0.10	25.6	
1 vs 0	GS	35-44	0.75	0.64	0.87	24.5	
1 vs 0	TwinsUK	35-44	0.41	0.30	0.53	24.6	
1 vs 0	BiB	35-44	0.27	0.21	0.33	25.3	
**1 vs 0**	**Overall**	**35-44**	**0.38**	**0.10**	**0.65**		**98.7**
1 vs 0	BCS70	45-54	-0.03	-0.07	0.02	20.3	
1 vs 0	USoc	45-54	0.04	0.03	0.05	20.5	
1 vs 0	ELSA	45-54	0.25	0.16	0.33	19.7	
1 vs 0	GS	45-54	0.50	0.42	0.57	19.8	
1 vs 0	TwinsUK	45-54	0.22	0.14	0.30	19.7	
**1 vs 0**	**Overall**	**45-54**	**0.19**	**0.01**	**0.37**		**98.7**
1 vs 0	NCDS	55-64	-0.04	-0.07	-0.01	20.4	
1 vs 0	USoc	55-64	0.04	0.03	0.06	20.6	
1 vs 0	ELSA	55-64	0.30	0.23	0.38	19.3	
1 vs 0	GS	55-64	0.22	0.16	0.27	19.9	
1 vs 0	TwinsUK	55-64	0.28	0.22	0.34	19.8	
**1 vs 0**	**Overall**	**55-64**	**0.16**	**0.02**	**0.29**		**98.5**
1 vs 0	NSHD	65-74	0.46	0.37	0.55	18.9	
1 vs 0	USoc	65-74	0.06	0.05	0.07	20.6	
1 vs 0	ELSA	65-74	0.28	0.24	0.33	20.1	
1 vs 0	GS	65-74	0.07	0.03	0.11	20.2	
1 vs 0	TwinsUK	65-74	0.18	0.14	0.23	20.2	
**1 vs 0**	**Overall**	**65-74**	**0.21**	**0.07**	**0.35**		**98.7**
1 vs 0	USoc	75+	0.04	0.03	0.06	34.8	
1 vs 0	ELSA	75+	0.15	0.09	0.22	23.1	

© 2022 Patel K et al. *JAMA Network Open.*

1 vs 0	GS	75+	0.08	0.00	0.17	16.8	
1 vs 0	TwinsUK	75+	0.09	0.03	0.14	25.3	
**1 vs 0**	**Overall**	**75+**	**0.09**	**0.04**	**0.14**		**75.7**
2 vs 0	MCS	16-24	0.16	0.10	0.21	44.2	
2 vs 0	USoc	16-24	0.04	0.02	0.07	48.1	
2 vs 0	TwinsUK	16-24	0.32	-0.05	0.69	7.7	
**2 vs 0**	**Overall**	**16-24**	**0.11**	**0.00**	**0.23**		**87.4**
2 vs 0	NS	25-34	0.21	0.14	0.27	26.1	
2 vs 0	USoc	25-34	0.06	0.04	0.08	26.4	
2 vs 0	GS	25-34	0.64	0.43	0.86	22.9	
2 vs 0	TwinsUK	25-34	0.57	0.42	0.72	24.6	
**2 vs 0**	**Overall**	**25-34**	**0.36**	**0.08**	**0.63**		**98.2**
2 vs 0	USoc	35-44	0.03	0.01	0.05	34.4	
2 vs 0	GS	35-44	0.46	0.32	0.59	32.5	
2 vs 0	TwinsUK	35-44	0.56	0.46	0.67	33.2	
**2 vs 0**	**Overall**	**35-44**	**0.35**	**0.02**	**0.67**		**97.7**
2 vs 0	BCS70		0.09	0.05	0.14	25.3	
2 vs 0	USoc	45-54	0.02	0.01	0.04	25.7	
2 vs 0	GS	45-54	0.41	0.32	0.49	24.4	
2 vs 0	TwinsUK	45-54	0.36	0.29	0.44	24.6	
**2 vs 0**	**Overall**	**45-54**	**0.22**	**0.03**	**0.41**		**98.5**
2 vs 0	NCDS	55-64	0.08	0.05	0.11	25.2	
2 vs 0	USoc	55-64	0.02	0.01	0.03	25.4	
2 vs 0	GS	55-64	0.17	0.11	0.24	24.6	
2 vs 0	TwinsUK	55-64	0.43	0.37	0.49	24.8	
**2 vs 0**	**Overall**	**55-64**	**0.18**	**0.00**	**0.35**		**99.1**
2 vs 0	NSHD	65-74	0.16	-0.08	0.40	18.7	
2 vs 0	USoc	65-74	0.04	0.03	0.05	27.4	
2 vs 0	GS	65-74	0.11	0.06	0.15	27.0	
2 vs 0	TwinsUK	65-74	0.43	0.39	0.48	27.0	
**2 vs 0**	**Overall**	**65-74**	**0.19**	**0.00**	**0.37**		**98.8**
2 vs 0	USoc	75+	0.04	0.02	0.05	34.3	
2 vs 0	GS	75+	0.17	0.07	0.27	32.1	
2 vs 0	TwinsUK	75+	0.41	0.36	0.47	33.6	
**2 vs 0**	**Overall**	**75+**	**0.21**	**-0.01**	**0.43**		**98.3**
3 vs 0	MCS	16-24	0.19	0.13	0.24	42.9	
3 vs 0	USoc	16-24	0.12	0.09	0.15	52.6	
3 vs 0	TwinsUK	16-24	0.46	0.08	0.84	2.9	
3 vs 0	BiB	16-24	-0.08	-0.59	0.45	1.6	
**3 vs 0**	**Overall**	**16-24**	**0.16**	**0.09**	**0.22**		**55.8**

© 2022 Patel K et al. *JAMA Network Open.*

3 vs 0	ALSPAC	25-34	-0.04	-0.08	-0.01	17.1	
3 vs 0	NS	25-34	0.25	0.19	0.32	17.0	
3 vs 0	USoc	25-34	0.14	0.11	0.16	17.1	
3 vs 0	GS	25-34	1.06	0.85	1.27	16.0	
3 vs 0	TwinsUK	25-34	0.61	0.46	0.77	16.5	
3 vs 0	BiB	25-34	0.02	-0.15	0.18	16.4	
**3 vs 0**	**Overall**	**25-34**	**0.33**	**0.00**	**0.66**		**99.5**
3 vs 0	USoc	35-44	0.11	0.09	0.13	25.5	
3 vs 0	GS	35-44	0.90	0.76	1.04	24.5	
3 vs 0	TwinsUK	35-44	0.66	0.56	0.77	24.9	
3 vs 0	BiB	35-44	0.31	0.22	0.40	25.1	
**3 vs 0**	**Overall**	**35-44**	**0.49**	**0.14**	**0.84**		**98.7**
3 vs 0	BCS70	45-54	0.02	-0.03	0.07	20.2	
3 vs 0	USoc	45-54	0.07	0.05	0.08	20.3	
3 vs 0	ELSA	45-54	0.52	0.42	0.61	19.8	
3 vs 0	GS	45-54	0.65	0.56	0.74	19.8	
3 vs 0	TwinsUK	45-54	0.51	0.43	0.59	19.9	
**3 vs 0**	**Overall**	**45-54**	**0.35**	**0.10**	**0.60**		**99.2**
3 vs 0	NCDS	55-64	0.05	0.00	0.09	20.1	
3 vs 0	USoc	55-64	0.06	0.05	0.07	20.3	
3 vs 0	ELSA	55-64	0.48	0.40	0.56	19.7	
3 vs 0	GS	55-64	0.41	0.35	0.48	19.9	
3 vs 0	TwinsUK	55-64	0.45	0.39	0.51	20.0	
**3 vs 0**	**Overall**	**55-64**	**0.29**	**0.10**	**0.48**		**99.0**
3 vs 0	NSHD	65-74	0.10	-0.02	0.21	18.5	
3 vs 0	USoc	65-74	0.09	0.08	0.10	20.7	
3 vs 0	ELSA	65-74	0.41	0.36	0.45	20.3	
3 vs 0	GS	65-74	0.28	0.23	0.32	20.3	
3 vs 0	TwinsUK	65-74	0.47	0.43	0.51	20.3	
**3 vs 0**	**Overall**	**65-74**	**0.27**	**0.12**	**0.42**		**98.8**
3 vs 0	USoc	75+	0.08	0.07	0.10	26.4	
3 vs 0	ELSA	75+	0.29	0.22	0.35	25.1	
3 vs 0	GS	75+	0.32	0.22	0.43	23.1	
3 vs 0	TwinsUK	75+	0.41	0.35	0.46	25.5	
**3 vs 0**	**Overall**	**75+**	**0.27**	**0.13**	**0.41**		**96.8**

© 2022 Patel K et al. *JAMA Network Open.*

eTable 10. Meta-analyzed Regression Coefficients: Continuous; Stratified by Education

Timepoint	Study	Strata	SMD	lower_ci	upper_ci	%Weight	%I2
1 vs 0	MCS	degree	0.10	0.04	0.15	9.2	
1 vs 0	ALSPAC	degree	-0.04	-0.08	0.00	9.3	
1 vs 0	NS	degree	0.30	0.20	0.40	8.8	
1 vs 0	BCS70	degree	-0.02	-0.06	0.03	9.3	
1 vs 0	NCDS	degree	-0.05	-0.09	-0.01	9.3	
1 vs 0	NSHD	degree	0.62	0.45	0.79	7.9	
1 vs 0	USoc	degree	0.08	0.07	0.09	9.4	
1 vs 0	ELSA	degree	0.30	0.23	0.37	9.1	
1 vs 0	GS	degree	0.34	0.29	0.38	9.3	
1 vs 0	TwinsUK	degree	0.26	0.23	0.30	9.3	
1 vs 0	BiB	degree	0.22	0.14	0.31	9.0	
**1 vs 0**	**Overall**	**degree**	**0.17**	**0.05**	**0.29**		**98.9**
1 vs 0	MCS	no degree	0.08	0.01	0.16	9.0	
1 vs 0	ALSPAC	no degree	-0.17	-0.24	-0.11	9.2	
1 vs 0	NS	no degree	0.28	0.13	0.43	8.0	
1 vs 0	BCS70	no degree	-0.02	-0.09	0.04	9.2	
1 vs 0	NCDS	no degree	-0.04	-0.09	0.01	9.3	
1 vs 0	NSHD	no degree	0.47	0.38	0.57	8.8	
1 vs 0	USoc	no degree	0.06	0.05	0.07	9.5	
1 vs 0	ELSA	no degree	0.24	0.20	0.28	9.3	
1 vs 0	GS	no degree	0.24	0.20	0.28	9.3	
1 vs 0	TwinsUK	no degree	0.17	0.13	0.21	9.3	
1 vs 0	BiB	no degree	0.22	0.16	0.29	9.1	
**1 vs 0**	**Overall**	**no degree**	**0.12**	**0.01**	**0.22**		**98.6**
2 vs 0	MCS	degree	0.10	0.04	0.16	13.2	
2 vs 0	NS	degree	0.27	0.19	0.34	13.0	
2 vs 0	BCS70	degree	0.12	0.08	0.17	13.5	
2 vs 0	NCDS	degree	0.07	0.04	0.11	13.5	
2 vs 0	NSHD	degree	0.74	0.37	1.11	6.3	
2 vs 0	USoc	degree	0.04	0.04	0.05	13.6	
2 vs 0	GS	degree	0.26	0.21	0.31	13.4	
2 vs 0	TwinsUK	degree	0.47	0.43	0.50	13.5	
**2 vs 0**	**Overall**	**degree**	**0.22**	**0.10**	**0.35**		**98.8**
2 vs 0	MCS	no degree	0.23	0.14	0.32	11.9	
2 vs 0	NS	no degree	0.16	0.07	0.25	11.9	
2 vs 0	BCS70	no degree	0.08	0.01	0.15	12.7	
2 vs 0	NCDS	no degree	0.07	0.03	0.12	13.2	
2 vs 0	NSHD	no degree	0.23	0.08	0.38	9.8	
2 vs 0	USoc	no degree	0.03	0.02	0.04	13.8	
2 vs 0	GS	no degree	0.22	0.17	0.27	13.3	

© 2022 Patel K et al. *JAMA Network Open.*

2 vs 0	TwinsUK	no degree	0.38	0.34	0.42	13.4	
**2 vs 0**	**Overall**	**no degree**	**0.17**	**0.08**	**0.26**		**96.4**
3 vs 0	MCS	degree	0.16	0.09	0.22	9.2	
3 vs 0	ALSPAC	degree	0.00	-0.05	0.04	9.4	
3 vs 0	NS	degree	0.32	0.24	0.41	9.0	
3 vs 0	BCS70	degree	0.09	0.04	0.14	9.3	
3 vs 0	NCDS	degree	0.05	0.01	0.09	9.4	
3 vs 0	NSHD	degree	0.48	0.31	0.65	7.9	
3 vs 0	USoc	degree	0.10	0.09	0.11	9.5	
3 vs 0	ELSA	degree	0.44	0.37	0.50	9.2	
3 vs 0	GS	degree	0.51	0.46	0.56	9.3	
3 vs 0	TwinsUK	degree	0.52	0.48	0.56	9.4	
3 vs 0	BiB	degree	0.20	0.09	0.31	8.5	
**3 vs 0**	**Overall**	**degree**	**0.26**	**0.14**	**0.38**		**98.7**
3 vs 0	MCS	no degree	0.22	0.13	0.31	8.9	
3 vs 0	ALSPAC	no degree	-0.14	-0.21	-0.07	9.2	
3 vs 0	NS	no degree	0.21	0.11	0.30	8.9	
3 vs 0	BCS70	no degree	-0.02	-0.09	0.05	9.2	
3 vs 0	NCDS	no degree	0.03	-0.04	0.10	9.2	
3 vs 0	NSHD	no degree	0.09	-0.04	0.21	8.5	
3 vs 0	USoc	no degree	0.08	0.07	0.09	9.5	
3 vs 0	ELSA	no degree	0.42	0.38	0.47	9.4	
3 vs 0	GS	no degree	0.42	0.38	0.47	9.3	
3 vs 0	TwinsUK	no degree	0.42	0.38	0.46	9.4	
3 vs 0	BiB	no degree	0.23	0.13	0.33	8.6	
**3 vs 0**	**Overall**	**no degree**	**0.18**	**0.06**	**0.30**		**98.4**

© 2022 Patel K et al. *JAMA Network Open.*

eTable 11. Meta-analyzed Regression Coefficients: Continuous; Stratified by Education

Timepoint	Study	Strata	SMD	lower_ci	upper_ci	%Weight	%I2
1 vs 0	MCS	white	0.07	0.01	0.12	12.5	
1 vs 0	ALSPAC	white	-0.09	-0.12	-0.05	12.8	
1 vs 0	NS	white	0.27	0.17	0.38	11.2	
1 vs 0	USoc	white	0.06	0.06	0.07	13.0	
1 vs 0	ELSA	white	0.27	0.24	0.31	12.8	
1 vs 0	GS	white	0.29	0.26	0.32	12.8	
1 vs 0	TwinsUK	white	0.22	0.19	0.24	12.8	
1 vs 0	BiB	white	0.31	0.24	0.38	12.1	
**1 vs 0**	**Overall**	**white**	**0.17**	**0.07**	**0.27**		**99.0**
1 vs 0	MCS	non-white	0.30	0.18	0.41	15.1	
1 vs 0	ALSPAC	non-white	-0.12	-0.35	0.11	9.5	
1 vs 0	NS	non-white	0.30	0.16	0.44	13.9	
1 vs 0	USoc	non-white	0.09	0.06	0.11	18.7	
1 vs 0	ELSA	non-white	0.05	-0.12	0.21	12.6	
1 vs 0	GS	non-white	0.37	-0.15	0.88	3.2	
1 vs 0	TwinsUK	non-white	0.36	0.12	0.59	9.3	
1 vs 0	BiB	non-white	0.16	0.10	0.22	17.7	
**1 vs 0**	**Overall**	**non-white**	**0.17**	**0.07**	**0.27**		**86.3**
2 vs 0	MCS	white	0.12	0.07	0.17	19.9	
2 vs 0	NS	white	0.19	0.12	0.26	19.3	
2 vs 0	USoc	white	0.03	0.03	0.04	20.4	
2 vs 0	GS	white	0.24	0.21	0.27	20.2	
2 vs 0	TwinsUK	white	0.43	0.40	0.45	20.3	
**2 vs 0**	**Overall**	**white**	**0.20**	**0.07**	**0.33**		**99.1**
2 vs 0	MCS	non-white	0.40	0.15	0.64	19.0	
2 vs 0	NS	non-white	0.27	0.16	0.38	25.7	
2 vs 0	USoc	non-white	0.04	0.02	0.06	28.1	
2 vs 0	GS	non-white	0.25	-0.33	0.83	7.6	
2 vs 0	TwinsUK	non-white	0.43	0.19	0.66	19.6	
**2 vs 0**	**Overall**	**non-white**	**0.25**	**0.06**	**0.43**		**87.3**
3 vs 0	MCS	white	0.17	0.11	0.22	12.5	
3 vs 0	ALSPAC	white	-0.05	-0.08	-0.01	12.6	
3 vs 0	NS	white	0.25	0.17	0.32	12.3	
3 vs 0	USoc	white	0.09	0.08	0.10	12.7	
3 vs 0	ELSA	white	0.44	0.40	0.48	12.6	
3 vs 0	GS	white	0.47	0.43	0.50	12.6	
3 vs 0	TwinsUK	white	0.48	0.45	0.50	12.7	
3 vs 0	BiB	white	0.36	0.27	0.45	12.0	
**3 vs 0**	**Overall**	**white**	**0.28**	**0.14**	**0.41**		**99.4**

3 vs 0	MCS	non-white	0.35	0.17	0.53	12.9	
3 vs 0	ALSPAC	non-white	-0.16	-0.37	0.05	11.9	
3 vs 0	NS	non-white	0.29	0.17	0.41	14.7	
3 vs 0	USoc	non-white	0.09	0.06	0.12	16.6	
3 vs 0	ELSA	non-white	0.26	0.08	0.45	12.8	
3 vs 0	GS	non-white	0.56	-0.02	1.14	4.2	
3 vs 0	TwinsUK	non-white	0.47	0.24	0.70	11.4	
3 vs 0	BiB	non-white	0.10	0.01	0.19	15.6	
**3 vs 0**	**Overall**	**non-white**	**0.21**	**0.07**	**0.35**		**88.8**

© 2022 Patel K et al. *JAMA Network Open.*

eTable 12. Meta-analyzed Regression Coefficients: Continuous; Stratified by Country

Timepoint	Study	Strata	SMD	lower_ci	upper_ci	%Weight	%I2
1 vs 0	MCS	england	0.11	0.05	0.16	10.0	
1 vs 0	ALSPAC	england	-0.08	-0.11	-0.05	10.1	
1 vs 0	NS	england	0.30	0.24	0.36	9.9	
1 vs 0	BCS70	england	-0.05	-0.08	-0.01	10.1	
1 vs 0	NCDS	england	-0.05	-0.09	-0.02	10.1	
1 vs 0	NSHD	england	0.42	0.32	0.53	9.3	
1 vs 0	USoc	england	0.06	0.06	0.07	10.2	
1 vs 0	ELSA	england	0.25	0.22	0.29	10.1	
1 vs 0	TwinsUK	england	0.21	0.19	0.24	10.2	
1 vs 0	BiB	england	0.22	0.17	0.26	10.0	
1 vs 0	Overall	england	0.14	0.04	0.24		99.2
1 vs 0	MCS	wales	0.13	-0.06	0.31	15.8	
1 vs 0	NS	wales	0.72	-0.14	1.58	1.3	
1 vs 0	BCS70	wales	-0.01	-0.14	0.12	22.3	
1 vs 0	NCDS	wales	-0.08	-0.22	0.06	20.8	
1 vs 0	NSHD	wales	0.56	0.14	0.98	4.7	
1 vs 0	USoc	wales	0.08	0.05	0.11	35.2	
1 vs 0	Overall	wales	0.06	-0.03	0.16		59.7
1 vs 0	MCS	scotland	0.14	0.01	0.27	15.3	
1 vs 0	NS	scotland	0.06	-0.35	0.46	7.1	
1 vs 0	BCS70	scotland	-0.10	-0.19	0.00	16.3	
1 vs 0	NCDS	scotland	0.01	-0.08	0.11	16.3	
1 vs 0	NSHD	scotland	0.55	0.25	0.84	9.7	
1 vs 0	USoc	scotland	0.07	0.05	0.09	17.6	
1 vs 0	GS	scotland	0.29	0.26	0.32	17.5	
1 vs 0	Overall	scotland	0.13	-0.01	0.27		35.6
1 vs 0	MCS	northern ireland	0.05	-0.11	0.21	13.1	
1 vs 0	USoc	northern ireland	0.10	0.04	0.16	87.0	
1 vs 0	Overall	northern ireland	0.09	0.04	0.15		0.0
2 vs 0	MCS	england	0.18	0.11	0.24	15.0	
2 vs 0	NS	england	0.22	0.15	0.28	15.0	
2 vs 0	BCS70	england	0.07	0.02	0.13	15.3	
2 vs 0	NCDS	england	0.08	0.04	0.11	15.6	
2 vs 0	NSHD	england	0.15	-0.12	0.42	7.7	
2 vs 0	USoc	england	0.03	0.03	0.04	15.8	
2 vs 0	TwinsUK	england	0.42	0.40	0.45	15.7	

© 2022 Patel K et al. *JAMA Network Open.*

2 vs 0	Overall	england	0.17	0.06	0.27		98.6
2 vs 0	MCS	wales	0.10	-0.06	0.25	16.6	
2 vs 0	NS	wales	0.19	-0.75	1.13	0.6	
2 vs 0	BCS70	wales	0.20	0.09	0.31	24.0	
2 vs 0	NCDS	wales	0.01	-0.20	0.22	10.0	
2 vs 0	NSHD	wales	0.26	-0.22	0.73	2.4	
2 vs 0	USoc	wales	0.06	0.03	0.09	46.5	
2 vs 0	Overall	wales	0.10	0.03	0.18		38.2
2 vs 0	MCS	scotland	0.13	0.03	0.24	16.1	
2 vs 0	NS	scotland	0.59	0.20	0.98	3.7	
2 vs 0	BCS70	scotland	0.15	0.04	0.26	15.7	
2 vs 0	NCDS	scotland	0.13	0.04	0.22	17.3	
2 vs 0	NSHD	scotland	0.31	-0.01	0.63	5.2	
2 vs 0	USoc	scotland	0.02	0.00	0.04	21.3	
2 vs 0	GS	scotland	0.24	0.21	0.27	20.8	
2 vs 0	Overall	scotland	0.16	0.08	0.24		90.6
2 vs 0	MCS	northern ireland	0.21	-0.05	0.47	17.5	
2 vs 0	USoc	northern ireland	0.05	-0.01	0.12	82.5	
2 vs 0	Overall	northern ireland	0.08	-0.04	0.20		26.6
3 vs 0	MCS	england	0.20	0.14	0.27	10.0	
3 vs 0	ALSPAC	england	-0.04	-0.08	-0.01	10.2	
3 vs 0	NS	england	0.26	0.20	0.33	10.0	
3 vs 0	BCS70	england	0.00	-0.05	0.06	10.1	
3 vs 0	NCDS	england	0.04	-0.01	0.09	10.1	
3 vs 0	NSHD	england	0.08	-0.05	0.21	9.0	
3 vs 0	USoc	england	0.09	0.08	0.10	10.3	
3 vs 0	ELSA	england	0.43	0.39	0.46	10.2	
3 vs 0	TwinsUK	england	0.47	0.45	0.50	10.3	
3 vs 0	BiB	england	0.23	0.16	0.29	10.0	
3 vs 0	Overall	england	0.18	0.07	0.29		99.1
3 vs 0	MCS	wales	0.13	-0.03	0.30	18.1	
3 vs 0	NS	wales	0.59	-0.34	1.52	1.6	
3 vs 0	BCS70	wales	0.11	0.01	0.22	22.0	
3 vs 0	NCDS	wales	-0.02	-0.20	0.15	17.4	
3 vs 0	NSHD	wales	0.45	0.24	0.67	14.5	
3 vs 0	USoc	wales	0.12	0.09	0.15	26.5	
3 vs 0	Overall	wales	0.15	0.03	0.27		77.8
3 vs 0	MCS	scotland	0.12	0.01	0.24	16.2	
3 vs 0	NS	scotland	0.30	-0.15	0.75	5.8	

© 2022 Patel K et al. *JAMA Network Open.*

3 vs 0	BCS70	scotland	0.09	-0.02	0.21	16.1	
3 vs 0	NCDS	scotland	0.10	0.00	0.20	16.7	
3 vs 0	NSHD	scotland	0.12	-0.21	0.45	8.5	
3 vs 0	USoc	scotland	0.06	0.04	0.08	18.4	
3 vs 0	GS	scotland	0.47	0.43	0.50	18.3	
3 vs 0	Overall	scotland	0.18	0.05	0.31		96.2
3 vs 0	MCS	northern ireland	0.11	-0.01	0.23	20.7	
3 vs 0	USoc	northern ireland	0.11	0.05	0.17	79.3	
3 vs 0	Overall	northern ireland	0.11	0.06	0.17		0.0

© 2022 Patel K et al. *JAMA Network Open.*

eTable 13. Meta-analyzed Regression Coefficients: Continuous; Sensitivity; Cohorts With All Time Points

	TIME	STRATA	SMD	LOWER CI	UPPER CI	%I2
UNSTRATIFIED	1	Overall	0.166	0.048	0.284	99.21
	2		0.173	0.081	0.265	98.5
	3		0.205	0.078	0.331	99.17

	TIME	STRATA	SMD	LOWER CI	UPPER CI	%I2
AGE	1	16-24	0.106	0.078	0.134	0.03
		25-34	0.435	0.096	0.773	98.09
		35-44	0.42	0.053	0.786	98.3
		45-54	0.188	-0.036	0.411	99.03
		55-64	0.127	-0.015	0.27	98.45
		65-74	0.192	0.018	0.367	98.79
		75+	0.06	0.031	0.088	22.18
	2	16-24	0.1	-0.072	0.272	93.91
		25-34	0.353	0.071	0.635	98.12
		35-44	0.34	0.006	0.674	97.83
		45-54	0.216	0.027	0.406	98.38
		55-64	0.174	-0.006	0.353	98.97
		65-74	0.186	0.001	0.371	98.69
		75+	0.209	-0.007	0.425	98.05
	3	16-24	0.161	0.018	0.305	90.15
		25-34	0.497	0.083	0.911	99.05
		35-44	0.555	0.092	1.018	98.79
		45-54	0.307	-0.002	0.616	99.33
		55-64	0.239	0.024	0.454	99.07
		65-74	0.236	0.059	0.413	98.67
		75+	0.27	0.079	0.462	97.49

	TIME	STRATA	SMD	LOWER CI	UPPER CI	%I2
SEX	1	Female	0.2	0.075	0.325	98.82
		Male	0.101	-0.005	0.207	97.54
	2	Female	0.204	0.109	0.299	97.68
		Male	0.133	0.043	0.223	95.57
	3	Female	0.242	0.105	0.378	98.79
		Male	0.154	0.049	0.258	96.81

EDUCATION	TIME	STRATA	SMD	LOWER	UPPER	%I2

© 2022 Patel K et al. *JAMA Network Open.*

				CI	CI	
	1	Degree	0.242	0.138	0.422	97.78
		No Degree	0.195	0.105	0.363	96.4
	2	Degree	0.223	0.096	0.35	98.76
		No Degree	0.169	0.084	0.255	96.42
	3	Degree	0.239	0.137	0.418	98.52
		No Degree	0.222	0.111	0.445	98.61

	TIME	STRATA	SMD	LOWER CI	UPPER CI	%I2
**ETHNICITY**	1	White	0.173	0.098	0.303	98.01
		Non-White	0.219	0.115	0.419	79.09
	2	White	0.201	0.068	0.333	99.1
		Non-White	0.246	0.059	0.432	87.27
	3	White	0.238	0.123	0.461	99.43
		Non-White	0.348	0.24	0.505	0

	TIME	STRATA	SMD	LOWER CI	UPPER CI	%I2
**COUNTRY**	1	England	0.131	-0.017	0.279	99.04
		Scotland	0.086	-0.034	0.205	71.37
		Wales	0.093	-0.051	0.237	90.53
		NI	0.122	0.066	0.178	0
	2	England	0.111	0.044	0.177	92.62
		Scotland	0.1	0.022	0.178	38.39
		Wales	0.133	0.039	0.228	78.13
		NI	0.077	-0.066	0.22	39.42
	3	England	0.112	0.031	0.192	94.32
		Scotland	0.148	0.026	0.27	77.72
		Wales	0.075	0.029	0.121	21.24
		NI	0.119	0.06	0.178	0

© 2022 Patel K et al. *JAMA Network Open.*

eTable 14. Meta-analyzed Regression Coefficients: Continuous; Sensitivity; Anxiety

	TIME	STRATA	SMD	LOWER CI	UPPER CI	%I2
**UNSTRATIFIED**	1	Overall	0.215	0.065	0.364	98.53
	2		0.089	-0.023	0.201	96
	3		0.262	0.103	0.42	98.45

	TIME	STRATA	SMD	LOWER CI	UPPER CI	%I2
**AGE**	1	16-24	-0.022	-0.363	0.32	-
		25-34	0.478	0.019	0.938	98.55
		35-44	0.583	0.114	1.052	97.68
		45-54	0.492	0.409	0.575	-
		55-64	0.192	0.13	0.255	-
		65-74	0.135	0.087	0.182	-
		75+	0.115	0.014	0.216	-
	2	16-24	-	-	-	-
		25-34	0.559	0.332	0.786	-
		35-44	0.439	0.294	0.583	-
		45-54	0.232	0.138	0.325	-
		55-64	0.055	-0.015	0.125	-
		65-74	0.053	0.002	0.105	-
		75+	0.097	-0.016	0.209	-
	3	16-24	0.238	-0.248	0.723	-
		25-34	0.445	0.066	0.823	95.52
		35-44	0.657	0.075	1.238	97.43
		45-54	0.561	0.464	0.658	-
		55-64	0.291	0.22	0.363	-
		65-74	0.221	0.169	0.274	-
		75+	0.203	0.086	0.32	-

	TIME	STRATA	SMD	LOWER CI	UPPER CI	%I2
**SEX**	1	Female	0.237	-0.002	0.475	99.06
		Male	0.068	-0.073	0.21	93.63
	2	Female	0.117	-0.046	0.28	97.11
		Male	0.036	-0.01	0.082	9.7
	3	Female	0.305	0.044	0.566	99.12
		Male	0.172	-0.018	0.362	96.32

© 2022 Patel K et al. *JAMA Network Open.*

	TIME	STRATA	SMD	LOWER CI	UPPER CI	%I2
**EDUCATION**	1	Degree	0.3	0.211	0.426	84.4
		No Degree	0.282	0.233	0.341	42.35
	2	Degree	0.161	0.063	0.414	19.09
		No Degree	0.107	0.058	0.197	97.12
	3	Degree	0.241	0.112	0.52	97.05
		No Degree	0.351	0.285	0.432	52.16

	TIME	STRATA	SMD	LOWER CI	UPPER CI	%I2
**ETHNICITY**	1	White	0.299	0.259	0.345	48.07
		Non-White	0.287	0.218	0.377	-
	2	White	0.086	0.022	0.329	74.45
		Non-White	-	-	-	-
	3	White	0.246	0.097	0.624	99.02
		Non-White	0.207	0.085	0.507	0

© 2022 Patel K et al. *JAMA Network Open.*

eTable 15. Meta-analyzed Regression Coefficients: Continuous; Sensitivity; Depression

	TIME	STRATA	SMD	LOWER CI	UPPER CI	%I2
UNSTRATIFIED	1	Overall	0.166	-0.023	0.355	99.36
	2		0.541	0.005	1.078	99.83
	3		0.394	0.083	0.704	99.71

	TIME	STRATA	SMD	LOWER CI	UPPER CI	%I2
AGE	1	16-24	-0.522	-0.842	-0.202	-
		25-34	0.339	-0.564	1.242	98.84
		35-44	0.324	-0.215	0.863	98.51
		45-54	0.345	0.149	0.541	91.84
		55-64	0.253	0.163	0.343	71.77
		65-74	0.158	-0.086	0.402	98.32
		75+	0.113	0.023	0.203	63.16
	2	16-24	-	-	-	-
		25-34	0.637	0.424	0.85	-
		35-44	0.402	0.27	0.535	-
		45-54	0.478	0.391	0.566	-
		55-64	0.221	0.156	0.286	-
		65-74	0.127	0.08	0.173	-
		75+	0.2	0.098	0.302	-
	3	16-24	-0.119	-0.712	0.473	-
		25-34	0.517	-0.486	1.521	97.89
		35-44	0.543	0.089	0.997	95.92
		45-54	0.576	0.457	0.694	69.16
		55-64	0.453	0.402	0.503	0
		65-74	0.34	0.207	0.472	93.74
		75+	0.314	0.237	0.39	39.4

	TIME	STRATA	SMD	LOWER CI	UPPER CI	%I2
SEX	1	Female	0.207	0.037	0.376	98.98
		Male	0.145	-0.026	0.316	97.4
	2	Female	0.574	0.094	1.054	99.7
		Male	0.473	-0.133	1.079	99.35
	3	Female	0.392	0.122	0.662	99.47
		Male	0.376	0.032	0.72	99.26

© 2022 Patel K et al. *JAMA Network Open.*

EDUCATION	TIME	STRATA	SMD	LOWER CI	UPPER CI	%I2
	1	Degree	0.349	0.232	0.527	94.78
		No Degree	0.272	0.2	0.37	89.8
	2	Degree	0.494	0.153	1.601	99.27
		No Degree	0.441	0.16	1.213	98.98
	3	Degree	0.508	0.298	0.864	98.59
		No Degree	0.471	0.301	0.736	98.37

ETHNICITY	TIME	STRATA	SMD	LOWER CI	UPPER CI	%I2
	1	White	0.307	0.213	0.441	96.84
		Non-White	0.451	0.419	0.485	-
	2	White	0.467	0.155	1.411	99.6
		Non-White	0.819	0.788	0.851	-
	3	White	0.513	0.34	0.775	99.07
		Non-White	0.583	0.195	1.745	58.01

© 2022 Patel K et al. *JAMA Network Open.*

eTable 16. Meta-analysed High Psychological Distress When Named Cohort Is Removed

Cohort	Timepoint	SMD	Lower CI	Upper CI	I2 (%)
All Cohorts	1	0.15	0.06	0.25	99.2
MCS	1	0.161	0.053	0.268	99.32
ALSPAC	1	0.178	0.082	0.273	99.08
NS	1	0.142	0.038	0.246	99.32
BCS	1	0.172	0.071	0.273	99.21
NCDS	1	0.174	0.075	0.273	99.15
NSHD	1	0.125	0.038	0.213	99.03
USoc	1	0.164	0.057	0.27	98.68
ELSA	1	0.144	0.038	0.25	99.26
GS	1	0.141	0.037	0.245	99.2
Twins	1	0.134	0.073	0.195	99.23
BIB	1	0.148	0.041	0.256	99.32
All Cohorts	2	0.175	0.085	0.265	98.6
MCS	2	0.177	0.073	0.281	98.85
ALSPAC	2	0.175	0.085	0.265	98.55
NS	2	0.17	0.067	0.274	98.86
BCS	2	0.187	0.087	0.288	98.72
NCDS	2	0.19	0.09	0.289	98.54
NSHD	2	0.176	0.078	0.274	98.86
USoc	2	0.197	0.104	0.29	96.98
ELSA	2	0.175	0.085	0.265	98.55
GS	2	0.165	0.063	0.268	98.62
Twins	2	0.134	0.073	0.195	95.21
BIB	2	0.175	0.085	0.265	98.55
All Cohorts	3	0.21	0.10	0.32	99.2
MCS	3	0.207	0.086	0.328	99.35
ALSPAC	3	0.23	0.122	0.339	99.12
NS	3	0.2	0.08	0.321	99.35
BCS	3	0.224	0.109	0.338	99.26
NCDS	3	0.221	0.105	0.337	99.27
NSHD	3	0.215	0.097	0.333	99.35
USoc	3	0.217	0.098	0.335	98.67
ELSA	3	0.183	0.071	0.294	99.17
GS	3	0.179	0.071	0.286	99.08
Twins	3	0.178	0.072	0.284	98.95
BIB	3	0.203	0.082	0.324	99.36

© 2022 Patel K et al. *JAMA Network Open.*

eTable 17. Meta-analyzed Regression Coefficients: Binary; Unstratified

Timepoint	Study	Strata	IRR	lower_ci	upper_ci	%Weight	%I2
1 vs 0	MCS	overall	1.14	1.02	1.28	9.2	
1 vs 0	ALSPAC	overall	0.81	0.75	0.87	9.4	
1 vs 0	NS	overall	1.49	1.31	1.69	9.1	
1 vs 0	BCS70	overall	0.86	0.77	0.97	9.2	
1 vs 0	NCDS	overall	0.81	0.73	0.89	9.3	
1 vs 0	NSHD	overall	2.49	1.82	3.39	7.8	
1 vs 0	USoc	overall	1.43	1.38	1.48	9.5	
1 vs 0	ELSA	overall	1.60	1.46	1.75	9.3	
1 vs 0	GS	overall	1.74	1.59	1.91	9.3	
1 vs 0	TwinsUK	overall	1.19	1.01	1.41	8.9	
1 vs 0	BiB	overall	1.61	1.42	1.83	9.1	
**1 vs 0**	**Overall**	**overall**	**1.29**	**1.05**	**1.58**		**98.1**
2 vs 0	MCS	overall	1.39	1.23	1.57	13.2	
2 vs 0	NS	overall	1.26	1.11	1.43	13.1	
2 vs 0	BCS70	overall	1.06	0.96	1.18	13.7	
2 vs 0	NCDS	overall	1.08	1.00	1.18	14.2	
2 vs 0	NSHD	overall	1.68	1.11	2.53	5.2	
2 vs 0	USoc	overall	1.10	1.06	1.15	15.1	
2 vs 0	GS	overall	1.58	1.43	1.75	13.7	
2 vs 0	TwinsUK	overall	1.06	0.90	1.24	11.8	
**2 vs 0**	**Overall**	**overall**	**1.23**	**1.09**	**1.38**		**87.3**
3 vs 0	MCS	overall	1.47	1.30	1.66	9.3	
3 vs 0	ALSPAC	overall	0.90	0.84	0.97	9.6	
3 vs 0	NS	overall	1.32	1.19	1.46	9.4	
3 vs 0	BCS70	overall	0.96	0.86	1.06	9.4	
3 vs 0	NCDS	overall	1.03	0.93	1.15	9.4	
3 vs 0	NSHD	overall	1.57	1.08	2.27	6.7	
3 vs 0	USoc	overall	1.42	1.36	1.48	9.7	
3 vs 0	ELSA	overall	2.13	1.96	2.32	9.5	
3 vs 0	GS	overall	2.09	1.90	2.31	9.4	
3 vs 0	TwinsUK	overall	1.20	1.02	1.41	9.0	
3 vs 0	BiB	overall	1.48	1.23	1.78	8.7	
**3 vs 0**	**Overall**	**overall**	**1.36**	**1.14**	**1.62**		**97.2**

© 2022 Patel K et al. *JAMA Network Open.*

eTable 18. Meta-analyzed Regression Coefficients: Binary; Stratified by Sex

Timepoint	Study	Strata	IRR	lower_ci	upper_ci	%Weight	%I2
1 vs 0	MCS	female	1.095	0.96	1.249	9.16	
1 vs 0	ALSPAC	female	0.815	0.752	0.884	9.34	
1 vs 0	NS	female	1.56	1.359	1.789	9.13	
1 vs 0	BCS70	female	0.896	0.77	1.044	9.06	
1 vs 0	NCDS	female	0.832	0.737	0.939	9.2	
1 vs 0	NSHD	female	3.021	2.234	4.086	8.05	
1 vs 0	USoc	female	1.463	1.401	1.527	9.43	
1 vs 0	ELSA	female	1.543	1.395	1.708	9.28	
1 vs 0	GS	female	1.805	1.613	2.019	9.24	
1 vs 0	TwinsUK	female	1.182	0.995	1.404	8.95	
1 vs 0	BiB	female	1.61	1.418	1.829	9.17	
**1 vs 0**	**Overall**	**female**	**1.325**	**1.062**	**1.653**		**97.9**
1 vs 0	MCS	male	1.158	0.897	1.493	10.44	
1 vs 0	ALSPAC	male	0.769	0.645	0.915	11.28	
1 vs 0	NS	male	1.302	0.954	1.777	9.76	
1 vs 0	BCS70	male	0.79	0.671	0.93	11.38	
1 vs 0	NCDS	male	0.751	0.62	0.911	11.11	
1 vs 0	NSHD	male	1.78	0.995	3.184	6.54	
1 vs 0	USoc	male	1.378	1.288	1.474	12.02	
1 vs 0	ELSA	male	1.706	1.425	2.043	11.23	
1 vs 0	GS	male	1.595	1.345	1.89	11.32	
1 vs 0	TwinsUK	male	1.535	0.718	3.282	4.93	
**1 vs 0**	**Overall**	**male**	**1.177**	**0.946**	**1.465**		**92.5**
2 vs 0	MCS	female	1.266	1.125	1.425	13.94	
2 vs 0	NS	female	1.286	1.117	1.482	13	
2 vs 0	BCS70	female	1.154	1.033	1.289	14.25	
2 vs 0	NCDS	female	1.11	1.006	1.226	14.71	
2 vs 0	NSHD	female	1.569	0.811	3.036	2.28	
2 vs 0	USoc	female	1.096	1.041	1.154	16.16	
2 vs 0	GS	female	1.636	1.446	1.851	13.74	
2 vs 0	TwinsUK	female	1.056	0.893	1.249	11.92	
**2 vs 0**	**Overall**	**female**	**1.222**	**1.098**	**1.361**		**84.7**
2 vs 0	MCS	male	1.75	1.356	2.259	12.88	
2 vs 0	NS	male	1.236	0.971	1.575	13.26	
2 vs 0	BCS70	male	0.918	0.747	1.128	14.28	
2 vs 0	NCDS	male	1.025	0.877	1.199	15.63	
2 vs 0	NSHD	male	1.804	1.15	2.829	8.06	
2 vs 0	USoc	male	1.116	1.031	1.208	17.31	
2 vs 0	GS	male	1.473	1.219	1.78	14.76	
2 vs 0	TwinsUK	male	1.228	0.561	2.685	3.81	

© 2022 Patel K et al. *JAMA Network Open.*

**2 vs 0**	**Overall**	**male**	**1.25**	**1.053**	**1.485**		**81.8**
3 vs 0	MCS	female	1.408	1.221	1.625	9.26	
3 vs 0	ALSPAC	female	0.899	0.829	0.975	9.72	
3 vs 0	NS	female	1.322	1.162	1.503	9.39	
3 vs 0	BCS70	female	1.047	0.941	1.165	9.55	
3 vs 0	NCDS	female	1.034	0.914	1.169	9.43	
3 vs 0	NSHD	female	1.395	0.9	2.162	5.83	
3 vs 0	USoc	female	1.435	1.367	1.506	9.87	
3 vs 0	ELSA	female	1.979	1.791	2.186	9.61	
3 vs 0	GS	female	2.165	1.924	2.436	9.47	
3 vs 0	TwinsUK	female	1.188	1.006	1.402	9.04	
3 vs 0	BiB	female	1.476	1.225	1.779	8.82	
**3 vs 0**	**Overall**	**female**	**1.348**	**1.144**	**1.589**		**95.9**
3 vs 0	MCS	male	1.595	1.247	2.039	10.47	
3 vs 0	ALSPAC	male	0.919	0.771	1.096	11.09	
3 vs 0	NS	male	1.308	1.096	1.561	11.08	
3 vs 0	BCS70	male	0.837	0.676	1.036	10.78	
3 vs 0	NCDS	male	1.029	0.846	1.251	10.93	
3 vs 0	NSHD	male	2.215	1.158	4.234	6.2	
3 vs 0	USoc	male	1.395	1.293	1.504	11.69	
3 vs 0	ELSA	male	2.442	2.068	2.882	11.17	
3 vs 0	GS	male	1.933	1.611	2.319	11.04	
3 vs 0	TwinsUK	male	1.786	0.865	3.687	5.54	
**3 vs 0**	**Overall**	**male**	**1.414**	**1.118**	**1.786**		**93.5**

© 2022 Patel K et al. *JAMA Network Open.*

eTable 19. Meta-analyzed Regression Coefficients: Binary; Stratified by Age

Timepoint	Study	Strata	IRR	lower_ci	upper_ci	%Weight	%I2
1 vs 0	MCS	16-24	1.14	1.02	1.28	42.8	
1 vs 0	USoc	16-24	1.38	1.24	1.53	44.1	
1 vs 0	TwinsUK	16-24	0.98	0.38	2.49	4.2	
1 vs 0	BiB	16-24	0.75	0.41	1.37	9.0	
**1 vs 0**	**Overall**	**16-24**	**1.19**	**0.97**	**1.45**		**69.9**
1 vs 0	ALSPAC	25-34	0.81	0.75	0.87	18.9	
1 vs 0	NS	25-34	1.49	1.31	1.69	18.5	
1 vs 0	USoc	25-34	1.52	1.38	1.68	18.8	
1 vs 0	GS	25-34	2.61	1.82	3.76	15.1	
1 vs 0	TwinsUK	25-34	1.22	0.68	2.17	11.4	
1 vs 0	BiB	25-34	1.41	1.13	1.76	17.4	
**1 vs 0**	**Overall**	**25-34**	**1.40**	**1.03**	**1.91**		**96.1**
1 vs 0	USoc	35-44	1.47	1.35	1.61	35.3	
1 vs 0	GS	35-44	2.29	1.76	2.99	23.7	
1 vs 0	TwinsUK	35-44	1.45	0.87	2.40	11.8	
1 vs 0	BiB	35-44	1.83	1.52	2.20	29.2	
**1 vs 0**	**Overall**	**35-44**	**1.74**	**1.41**	**2.15**		**74.7**
1 vs 0	BCS70	45-54	0.86	0.77	0.97	21.8	
1 vs 0	USoc	45-54	1.31	1.22	1.42	22.2	
1 vs 0	ELSA	45-54	1.54	1.26	1.89	20.4	
1 vs 0	GS	45-54	2.10	1.71	2.59	20.3	
1 vs 0	TwinsUK	45-54	1.21	0.69	1.73	16.4	
**1 vs 0**	**Overall**	**45-54**	**1.35**	**1.00**	**1.82**		**94.7**
1 vs 0	NCDS	55-65	0.81	0.73	0.89	21.6	
1 vs 0	USoc	55-65	1.35	1.25	1.46	21.9	
1 vs 0	ELSA	55-65	1.62	1.36	1.92	20.2	
1 vs 0	GS	55-65	1.46	1.23	1.72	20.4	
1 vs 0	TwinsUK	55-65	1.34	0.96	1.87	16.0	
**1 vs 0**	**Overall**	**55-65**	**1.27**	**0.99**	**1.63**		**94.0**
1 vs 0	NSHD	65-74	2.49	1.82	3.39	16.7	
1 vs 0	USoc	65-74	1.67	1.51	1.86	23.6	
1 vs 0	ELSA	65-74	1.86	1.61	2.15	22.5	
1 vs 0	GS	65-74	1.41	1.17	1.70	21.1	
1 vs 0	TwinsUK	65-74	1.13	0.81	1.57	16.1	
**1 vs 0**	**Overall**	**65-74**	**1.66**	**1.33**	**2.07**		**86.4**
1 vs 0	USoc	75+	1.47	1.22	1.76	45.1	
1 vs 0	ELSA	75+	1.38	1.15	1.65	44.6	

© 2022 Patel K et al. *JAMA Network Open.*

1 vs 0	GS	75+	1.81	1.11	2.96	6.2	
1 vs 0	TwinsUK	75+	1.16	0.64	2.10	4.2	
**1 vs 0**	**Overall**	**75+**	**1.43**	**1.27**	**1.62**		**0.0**
2 vs 0	MCS	16-24	1.39	1.23	1.57	43.9	
2 vs 0	USoc	16-24	0.86	0.74	1.00	42.7	
2 vs 0	TwinsUK	16-24	0.84	0.35	2.02	13.4	
**2 vs 0**	**Overall**	**16-24**	**1.06**	**0.72**	**1.55**		**89.8**
2 vs 0	NS	25-34	1.26	1.11	1.43	47.5	
2 vs 0	USoc	25-34	1.16	1.03	1.32	46.4	
2 vs 0	GS	25-34	1.76	1.12	2.75	3.6	
2 vs 0	TwinsUK	25-34	1.10	0.63	1.89	2.4	
**2 vs 0**	**Overall**	**25-34**	**1.23**	**1.13**	**1.34**		**0.0**
2 vs 0	USoc	35-44	1.05	0.94	1.18	40.9	
2 vs 0	GS	35-44	1.81	1.33	2.45	33.3	
2 vs 0	TwinsUK	35-44	1.73	1.09	2.76	25.8	
**2 vs 0**	**Overall**	**35-44**	**1.43**	**0.98**	**2.09**		**92.0**
2 vs 0	BCS70	45-54	1.06	0.96	1.18	28.3	
2 vs 0	USoc	45-54	1.07	0.98	1.17	28.5	
2 vs 0	GS	45-54	1.90	1.52	2.39	24.9	
2 vs 0	TwinsUK	45-54	1.00	0.66	1.53	18.3	
**2 vs 0**	**Overall**	**45-54**	**1.22**	**0.91**	**1.63**		**93.0**
2 vs 0	NCDS	55-65	1.08	1.00	1.18	33.4	
2 vs 0	USoc	55-65	1.08	0.99	1.17	33.1	
2 vs 0	GS	55-65	1.40	1.17	1.69	22.5	
2 vs 0	TwinsUK	55-65	0.91	0.65	1.29	11.0	
**2 vs 0**	**Overall**	**55-65**	**1.13**	**0.98**	**1.29**		**74.6**
2 vs 0	NSHD	65-74	1.68	1.11	2.53	5.0	
2 vs 0	USoc	65-74	1.33	1.19	1.49	66.3	
2 vs 0	GS	65-74	1.42	1.16	1.73	21.2	
2 vs 0	TwinsUK	65-74	1.04	0.74	1.45	7.6	
**2 vs 0**	**Overall**	**65-74**	**1.34**	**1.22**	**1.47**		**0.0**
2 vs 0	USoc	75+	1.40	1.17	1.68	83.1	
2 vs 0	GS	75+	1.58	0.92	2.71	9.2	
2 vs 0	TwinsUK	75+	1.35	0.75	2.44	7.7	
**2 vs 0**	**Overall**	**75+**	**1.42**	**1.20**	**1.67**		**0.0**
3 vs 0	MCS	16-24	1.47	1.30	1.66	48.6	
3 vs 0	USoc	16-24	1.20	1.05	1.38	46.4	
3 vs 0	TwinsUK	16-24	1.13	0.47	2.72	4.1	
3 vs 0	BiB	16-24	0.61	0.09	4.35	0.9	
**3 vs 0**	**Overall**	**16-24**	**1.32**	**1.10**	**1.58**		**52.7**

© 2022 Patel K et al. *JAMA Network Open.*

3 vs 0	ALSPAC	25-34	0.90	0.84	0.97	20.8	
3 vs 0	NS	25-34	1.32	1.19	1.46	20.5	
3 vs 0	USoc	25-34	1.40	1.23	1.59	20.2	
3 vs 0	GS	25-34	2.54	1.69	3.81	14.2	
3 vs 0	TwinsUK	25-34	1.15	0.66	2.02	11.0	
3 vs 0	BiB	25-34	1.13	0.72	1.78	13.2	
**3 vs 0**	**Overall**	**25-34**	**1.31**	**1.00**	**1.71**		**93.9**
3 vs 0	USoc	35-44	1.48	1.34	1.64	35.1	
3 vs 0	GS	35-44	2.47	1.85	3.31	24.0	
3 vs 0	TwinsUK	35-44	1.87	1.17	3.00	15.1	
3 vs 0	BiB	35-44	1.68	1.29	2.18	25.8	
**3 vs 0**	**Overall**	**35-44**	**1.79**	**1.41**	**2.27**		**72.1**
3 vs 0	BCS70	45-54	0.96	0.86	1.06	21.2	
3 vs 0	USoc	45-54	1.28	1.18	1.39	21.3	
3 vs 0	ELSA	45-54	2.40	1.95	2.96	20.2	
3 vs 0	GS	45-54	2.54	2.04	3.17	20.1	
3 vs 0	TwinsUK	45-54	1.36	0.91	2.05	17.3	
**3 vs 0**	**Overall**	**45-54**	**1.59**	**1.08**	**2.32**		**96.8**
3 vs 0	NCDS	55-65	1.03	0.93	1.15	21.3	
3 vs 0	USoc	55-65	1.30	1.20	1.42	21.54	
3 vs 0	ELSA	55-65	2.08	1.76	2.46	20.34	
3 vs 0	GS	55-65	1.74	1.46	2.07	20.21	
3 vs 0	TwinsUK	55-65	0.99	0.71	1.39	16.61	
**3 vs 0**	**Overall**	**55-65**	**1.38**	**1.04**	**1.83**		**96.5**
3 vs 0	NSHD	65-74	1.57	1.08	2.27	15.43	
3 vs 0	USoc	65-74	1.84	1.65	2.04	23.48	
3 vs 0	ELSA	65-74	2.39	2.09	2.73	22.89	
3 vs 0	GS	65-74	1.89	1.57	2.29	21.3	
3 vs 0	TwinsUK	65-74	1.10	0.80	1.53	16.9	
**3 vs 0**	**Overall**	**65-74**	**1.76**	**1.38**	**2.23**		**87.9**
3 vs 0	USoc	75+	1.88	1.59	2.23	42.73	
3 vs 0	ELSA	75+	1.73	1.48	2.03	48.75	
3 vs 0	GS	75+	2.55	1.53	4.24	4.64	
3 vs 0	TwinsUK	75+	1.50	0.86	2.62	3.88	
**3 vs 0**	**Overall**	**75+**	**1.82**	**1.63**	**2.03**		**0.0**

© 2022 Patel K et al. *JAMA Network Open.*

eTable 20. Meta-analyzed Regression Coefficients: Binary; Stratified by Education

Timepoint	Study	Strata	IRR	lower_ci	upper_ci	%Weight	%I2
1 vs 0	MCS	Degree	1.238	1.056	1.451	9.34	
1 vs 0	ALSPAC	Degree	0.82	0.737	0.912	9.53	
1 vs 0	NS	Degree	1.507	1.271	1.787	9.29	
1 vs 0	BCS70	Degree	0.841	0.729	0.969	9.41	
1 vs 0	NCDS	Degree	0.806	0.69	0.942	9.35	
1 vs 0	NSHD	Degree	3.678	2.448	5.526	7.74	
1 vs 0	USoc	Degree	1.546	1.467	1.629	9.66	
1 vs 0	ELSA	Degree	1.836	1.418	2.378	8.8	
1 vs 0	GS	Degree	1.911	1.661	2.198	9.42	
1 vs 0	TwinsUK	Degree	1.276	1.006	1.618	8.93	
1 vs 0	BiB	Degree	1.587	1.177	2.139	8.53	
**1 vs 0**	**Overall**	**Degree**	**1.379**	**1.072**	**1.773**		**97.1**
1 vs 0	MCS	No Degree	1.061	0.886	1.271	9	
1 vs 0	ALSPAC	No Degree	0.754	0.667	0.852	9.26	
1 vs 0	NS	No Degree	1.493	1.227	1.816	8.92	
1 vs 0	BCS70	No Degree	0.9	0.768	1.056	9.11	
1 vs 0	NCDS	No Degree	0.817	0.713	0.936	9.21	
1 vs 0	NSHD	No Degree	3.123	2.542	3.838	8.86	
1 vs 0	USoc	No Degree	1.361	1.295	1.431	9.45	
1 vs 0	ELSA	No Degree	1.558	1.415	1.716	9.35	
1 vs 0	GS	No Degree	1.617	1.42	1.84	9.23	
1 vs 0	TwinsUK	No Degree	1.175	0.907	1.52	8.54	
1 vs 0	BiB	No Degree	1.724	1.458	2.04	9.06	
**1 vs 0**	**Overall**	**No Degree**	**1.304**	**1.028**	**1.656**		**97.4**
2 vs 0	MCS	Degree	1.311	1.09	1.577	13.06	
2 vs 0	NS	Degree	1.343	1.113	1.62	13.03	
2 vs 0	BCS70	Degree	1.127	0.988	1.285	13.59	
2 vs 0	NCDS	Degree	1.064	0.936	1.209	13.62	
2 vs 0	NSHD	Degree	5.46	2.839	10.498	6.85	
2 vs 0	USoc	Degree	1.204	1.134	1.279	14.05	
2 vs 0	GS	Degree	1.711	1.466	1.997	13.38	
2 vs 0	TwinsUK	Degree	1.124	0.886	1.427	12.41	
**2 vs 0**	**Overall**	**Degree**	**1.387**	**1.093**	**1.76**		**95.2**
2 vs 0	MCS	No Degree	1.468	1.267	1.699	12.95	
2 vs 0	NS	No Degree	1.207	1.029	1.417	12.68	
2 vs 0	BCS70	No Degree	1.042	0.906	1.199	13.08	
2 vs 0	NCDS	No Degree	1.104	0.982	1.24	13.52	
2 vs 0	NSHD	No Degree	2.035	1.552	2.668	10.15	
2 vs 0	USoc	No Degree	1.041	0.979	1.106	14.31	
2 vs 0	GS	No Degree	1.497	1.298	1.727	13.03	

© 2022 Patel K et al. *JAMA Network Open.*

2 vs 0	TwinsUK	No Degree	0.997	0.766	1.299	10.3	
**2 vs 0**	**Overall**	**No Degree**	**1.249**	**1.069**	**1.46**		**90.4**
3 vs 0	MCS	Degree	1.53	1.277	1.834	9.39	
3 vs 0	ALSPAC	Degree	0.94	0.842	1.048	9.75	
3 vs 0	NS	Degree	1.43	1.22	1.676	9.52	
3 vs 0	BCS70	Degree	1.094	0.959	1.247	9.66	
3 vs 0	NCDS	Degree	1.086	0.947	1.245	9.63	
3 vs 0	NSHD	Degree	3.648	2.291	5.808	7.06	
3 vs 0	USoc	Degree	1.545	1.458	1.638	9.91	
3 vs 0	ELSA	Degree	2.534	2.043	3.142	9.16	
3 vs 0	GS	Degree	2.349	2.027	2.721	9.58	
3 vs 0	TwinsUK	Degree	1.291	1.02	1.633	9.02	
3 vs 0	BiB	Degree	1.355	0.875	2.097	7.31	
**3 vs 0**	**Overall**	**Degree**	**1.539**	**1.225**	**1.935**		**96.3**
3 vs 0	MCS	No Degree	1.425	1.198	1.696	9.1	
3 vs 0	ALSPAC	No Degree	0.829	0.735	0.936	9.53	
3 vs 0	NS	No Degree	1.241	1.082	1.424	9.41	
3 vs 0	BCS70	No Degree	0.903	0.785	1.038	9.4	
3 vs 0	NCDS	No Degree	1.031	0.891	1.192	9.35	
3 vs 0	NSHD	No Degree	1.197	0.872	1.644	7.57	
3 vs 0	USoc	No Degree	1.343	1.269	1.421	9.87	
3 vs 0	ELSA	No Degree	2.068	1.882	2.273	9.7	
3 vs 0	GS	No Degree	1.928	1.68	2.212	9.41	
3 vs 0	TwinsUK	No Degree	1.089	0.843	1.407	8.27	
3 vs 0	BiB	No Degree	1.576	1.231	2.019	8.36	
**3 vs 0**	**Overall**	**No Degree**	**1.282**	**1.073**	**1.531**		**95.1**

© 2022 Patel K et al. *JAMA Network Open.*

eTable 21. Meta-analyzed Regression Coefficients: Binary; Stratified by Ethnicity

Timepoint	Study	Strata	IRR	lower_ci	upper_ci	%Weight	%I2
1 vs 0	MCS	White	1.124	0.99	1.276	11.35	
1 vs 0	ALSPAC	White	0.808	0.747	0.874	11.67	
1 vs 0	NS	White	1.487	1.276	1.733	11.12	
1 vs 0	NSHD	White	2.486	1.821	3.393	9.26	
1 vs 0	USoc	White	1.435	1.38	1.491	11.83	
1 vs 0	ELSA	White	1.691	1.541	1.854	11.59	
1 vs 0	GS	White	1.736	1.579	1.909	11.58	
1 vs 0	TwinsUK	White	1.209	1.017	1.437	10.93	
1 vs 0	BiB	White	1.703	1.398	2.076	10.66	
**1 vs 0**	**Overall**	**White**	**1.436**	**1.174**	**1.757**		**98.6**
1 vs 0	MCS	Non-White	1.264	0.912	1.752	14.2	
1 vs 0	ALSPAC	Non-White	0.673	0.446	1.016	11.89	
1 vs 0	NS	Non-White	1.51	1.21	1.885	17.25	
1 vs 0	USoc	Non-White	1.397	1.247	1.566	19.95	
1 vs 0	ELSA	Non-White	1.037	0.756	1.422	14.5	
1 vs 0	TwinsUK	Non-White	2.216	0.782	6.275	3.54	
1 vs 0	BiB	Non-White	1.604	1.354	1.901	18.67	
**1 vs 0**	**Overall**	**Non-White**	**1.278**	**1.032**	**1.585**		**79.9**
2 vs 0	MCS	White	1.345	1.192	1.517	18.66	
2 vs 0	NS	White	1.257	1.082	1.461	17.16	
2 vs 0	NSHD	White	1.678	1.111	2.533	6.94	
2 vs 0	USoc	White	1.116	1.065	1.168	21.58	
2 vs 0	GS	White	1.574	1.417	1.749	19.4	
2 vs 0	TwinsUK	White	1.087	0.919	1.286	16.25	
**2 vs 0**	**Overall**	**White**	**1.291**	**1.133**	**1.472**		**85.5**
2 vs 0	MCS	Non-White	1.706	1.15	2.53	22.95	
2 vs 0	NS	Non-White	1.307	1.077	1.587	34.22	
2 vs 0	USoc	Non-White	0.957	0.834	1.098	37.12	
2 vs 0	TwinsUK	Non-White	1.159	0.382	3.519	5.71	
**2 vs 0**	**Overall**	**Non-White**	**1.229**	**0.923**	**1.637**		**75.5**
3 vs 0	MCS	White	1.4	1.247	1.572	11.64	
3 vs 0	ALSPAC	White	0.901	0.835	0.973	11.93	
3 vs 0	NS	White	1.317	1.165	1.489	11.58	
3 vs 0	NSHD	White	1.565	1.08	2.269	8.3	
3 vs 0	USoc	White	1.444	1.383	1.507	12.09	
3 vs 0	ELSA	White	2.242	2.049	2.454	11.84	

© 2022 Patel K et al. *JAMA Network Open.*

3 vs 0	GS	White	2.098	1.897	2.32	11.77	
3 vs 0	TwinsUK	White	1.226	1.038	1.448	11.12	
3 vs 0	BiB	White	1.497	1.141	1.965	9.73	
**3 vs 0**	**Overall**	**White**	**1.47**	**1.216**	**1.776**		**96.8**
3 vs 0	MCS	Non-White	1.964	1.251	3.086	9.8	
3 vs 0	ALSPAC	Non-White	0.805	0.57	1.137	13.13	
3 vs 0	NS	Non-White	1.323	1.098	1.593	19.85	
3 vs 0	USoc	Non-White	1.135	0.985	1.307	21.77	
3 vs 0	ELSA	Non-White	1.506	1.166	1.944	16.74	
3 vs 0	TwinsUK	Non-White	1.621	0.569	4.617	2.68	
3 vs 0	BiB	Non-White	1.362	1.037	1.787	16.03	
**3 vs 0**	**Overall**	**Non-White**	**1.286**	**1.073**	**1.542**		**66.3**

© 2022 Patel K et al. *JAMA Network Open.*

eTable 22. Meta-analyzed Regression Coefficients: Binary; Stratified by Country

Timepoint	Study	Strata	IRR	lower_ci	upper_ci	%Weight	%I2
1 vs 0	MCS	england	1.16	1.00	1.34	9.9	
1 vs 0	ALSPAC	england	0.81	0.75	0.87	10.1	
1 vs 0	NS	england	1.48	1.33	1.65	10.1	
1 vs 0	BCS70	england	0.81	0.73	0.89	10.1	
1 vs 0	NCDS	england	0.79	0.70	0.88	10.0	
1 vs 0	NSHD	england	3.25	2.60	4.05	9.5	
1 vs 0	USoc	england	1.42	1.36	1.47	10.2	
1 vs 0	ELSA	england	1.60	1.46	1.75	10.1	
1 vs 0	TwinsUK	england	1.19	1.01	1.41	9.8	
1 vs 0	BiB	england	1.24	1.19	1.30	10.2	
**1 vs 0**	**Overall**	**england**	**1.25**	**0.97**	**1.61**		**99.0**
1 vs 0	MCS	wales	1.41	0.94	2.11	20.0	
1 vs 0	NS	wales	1.87	0.27	13.03	3.3	
1 vs 0	BCS70	wales	0.90	0.61	1.31	20.6	
1 vs 0	NCDS	wales	0.79	0.54	1.16	20.6	
1 vs 0	NSHD	wales	3.36	1.37	8.22	10.6	
1 vs 0	USoc	wales	1.63	1.40	1.89	24.9	
**1 vs 0**	**Overall**	**wales**	**1.31**	**0.90**	**1.91**		**78.9**
1 vs 0	MCS	scotland	1.22	0.92	1.61	16.0	
1 vs 0	NS	scotland	0.86	0.20	3.72	6.7	
1 vs 0	BCS70	scotland	0.64	0.44	0.93	15.3	
1 vs 0	NCDS	scotland	0.80	0.57	1.11	15.6	
1 vs 0	NSHD	scotland	5.08	2.66	9.70	13.0	
1 vs 0	USoc	scotland	1.38	1.21	1.57	16.7	
1 vs 0	GS	scotland	1.74	1.59	1.91	16.8	
**1 vs 0**	**Overall**	**scotland**	**1.32**	**0.81**	**2.14**		**96.6**
1 vs 0	MCS	northern ireland	1.26	0.85	1.89	38.5	
1 vs 0	USoc	northern ireland	1.77	1.38	2.27	61.5	
**1 vs 0**	**Overall**	**northern ireland**	**1.56**	**1.13**	**2.14**		**49.2**
2 vs 0	MCS	england	1.42	1.23	1.63	14.5	
2 vs 0	NS	england	1.28	1.12	1.45	15.1	
2 vs 0	BCS70	england	1.03	0.91	1.16	15.5	
2 vs 0	NCDS	england	1.09	0.99	1.19	16.7	
2 vs 0	NSHD	england	1.78	1.26	2.50	6.7	
2 vs 0	USoc	england	1.10	1.05	1.15	18.3	
2 vs 0	TwinsUK	england	1.06	0.90	1.24	13.3	
**2 vs 0**	**Overall**	**england**	**1.18**	**1.06**	**1.32**		**84.1**
2 vs 0	MCS	wales	1.16	0.83	1.60	17.1	
2 vs 0	NS	wales	1.09	0.11	10.65	0.4	

© 2022 Patel K et al. *JAMA Network Open.*

2 vs 0	BCS70	wales	1.27	1.00	1.62	26.6	
2 vs 0	NCDS	wales	0.89	0.65	1.22	18.4	
2 vs 0	NSHD	wales	2.09	0.97	4.49	3.7	
2 vs 0	USoc	wales	1.30	1.07	1.59	33.8	
**2 vs 0**	**Overall**	**wales**	**1.20**	**1.03**	**1.40**		**20.1**
2 vs 0	MCS	scotland	1.39	1.01	1.91	15.5	
2 vs 0	NS	scotland	2.35	0.66	8.37	3.5	
2 vs 0	BCS70	scotland	1.05	0.85	1.30	17.6	
2 vs 0	NCDS	scotland	1.11	0.84	1.47	16.2	
2 vs 0	NSHD	scotland	3.42	1.82	6.45	9.3	
2 vs 0	USoc	scotland	1.00	0.85	1.16	18.6	
2 vs 0	GS	scotland	1.58	1.43	1.75	19.3	
**2 vs 0**	**Overall**	**scotland**	**1.36**	**1.05**	**1.77**		**87.5**
2 vs 0	MCS	northern ireland	1.74	1.02	2.98	29.2	
2 vs 0	USoc	northern ireland	1.24	0.90	1.70	70.8	
**2 vs 0**	**Overall**	**northern ireland**	**1.37**	**1.01**	**1.86**		**14.1**
3 vs 0	MCS	england	1.52	1.31	1.76	9.8	
3 vs 0	ALSPAC	england	0.90	0.84	0.97	10.5	
3 vs 0	NS	england	1.33	1.20	1.48	10.3	
3 vs 0	BCS70	england	0.91	0.81	1.03	10.1	
3 vs 0	NCDS	england	1.02	0.90	1.15	10.1	
3 vs 0	NSHD	england	1.11	0.82	1.50	7.9	
3 vs 0	USoc	england	1.42	1.36	1.49	10.6	
3 vs 0	ELSA	england	2.13	1.96	2.32	10.4	
3 vs 0	TwinsUK	england	1.20	1.02	1.41	9.7	
3 vs 0	BiB	england	1.25	1.18	1.34	10.5	
**3 vs 0**	**Overall**	**england**	**1.24**	**1.05**	**1.47**		**96.9**
3 vs 0	MCS	wales	1.53	1.16	2.02	23.1	
3 vs 0	NS	wales	2.88	0.44	18.74	2.7	
3 vs 0	BCS70	wales	1.18	0.86	1.61	22.0	
3 vs 0	NCDS	wales	0.89	0.64	1.25	21.4	
3 vs 0	NSHD	wales	8.94	2.35	34.09	4.9	
3 vs 0	USoc	wales	1.59	1.36	1.85	26.0	
**3 vs 0**	**Overall**	**wales**	**1.44**	**1.04**	**1.99**		**78.0**
3 vs 0	MCS	scotland	1.24	0.98	1.57	17.6	
3 vs 0	NS	scotland	1.00	0.22	4.53	2.1	
3 vs 0	BCS70	scotland	1.14	0.96	1.35	19.5	
3 vs 0	NCDS	scotland	1.16	0.87	1.54	16.2	
3 vs 0	NSHD	scotland	1.66	0.55	5.06	3.5	
3 vs 0	USoc	scotland	1.21	1.06	1.39	20.2	
3 vs 0	GS	scotland	2.09	1.90	2.31	21.0	
**3 vs 0**	**Overall**	**scotland**	**1.35**	**1.07**	**1.69**		**85.8**

© 2022 Patel K et al. *JAMA Network Open.*

3 vs 0	MCS	northern ireland	1.56	1.20	2.02	51.5	
3 vs 0	USoc	northern ireland	1.71	1.31	2.24	48.5	
**3 vs 0**	**Overall**	**northern ireland**	**1.63**	**1.36**	**1.97**		**0.0**

© 2022 Patel K et al. *JAMA Network Open.*

eTable 23. Meta-analyzed Regression Coefficients: Interactions; Sex

Time	Study	SMD	Lower CI	Upper CI	Weight	I2
1	MCS	0.086	-0.022	0.194	8.45	
1	ALSPAC	-0.011	-0.078	0.056	11.02	
1	NS	0.196	0.001	0.39	4.58	
1	BCS70	0.113	0.035	0.19	10.33	
1	NCDS	-0.03	-0.094	0.034	11.2	
1	NSHD	0.089	-0.008	0.186	9.15	
1	USOC	0.03	0.018	0.042	13.48	
1	ELSA	0.045	-0.024	0.114	10.87	
1	GS	0.22	0.159	0.281	11.39	
1	TwinsUK	0.11	0.02	0.2	9.54	
**1**	**Overall**	0.076	0.025	0.127		**84.93**
2	MCS	0.051	-0.066	0.168	10.26	
2	NS	0.008	-0.117	0.133	9.57	
2	BCS70	0.162	0.072	0.253	12.79	
2	NCDS	0.066	-0.001	0.133	15.38	
2	NSHD	-0.019	-0.29	0.251	3.23	
2	USOC	0.004	-0.008	0.016	20.19	
2	GS	0.172	0.104	0.24	15.28	
2	TwinsUK	0.037	-0.049	0.123	13.3	
**2**	**Overall**	**0.068**	**0.015**	**0.121**		**75.70**
3	MCS	0.144	0.024	0.263	8.99	
3	ALSPAC	-0.057	-0.13	0.015	11.77	
3	NS	0.034	-0.096	0.165	8.37	
3	BCS70	0.078	-0.02	0.176	10.24	
3	NCDS	0.037	-0.049	0.124	10.93	
3	NSHD	0.19	-0.445	0.825	0.8	
3	USOC	0.025	0.012	0.038	14.28	
3	ELSA	0.094	0.021	0.168	11.71	
3	GS	0.248	0.177	0.318	11.91	
3	TwinsUK	0.08	-0.005	0.166	11	
**3**	**Overall**	0.076	0.017	0.134		**83.88**

© 2022 Patel K et al. *JAMA Network Open.*

eTable 24. Meta-analyzed Regression Coefficients: Interactions; Ethnicity

Time	Study	SMD	Lower CI	Upper CI	Weight	I2
1	MCS	0.234	0.108	0.359	16.31	
1	ALSPAC	-0.033	-0.265	0.199	11.45	
1	NS	0.021	-0.157	0.2	13.82	
1	USOC	0.023	-0.001	0.048	19.62	
1	ELSA	-0.223	-0.39	-0.056	14.36	
1	GS	0.083	-0.293	0.459	6.8	
1	BiB	-0.149	-0.244	-0.055	17.65	
**1**	**Overall**	-0.011	-0.132	0.11		**85.8**
2	MCS	0.276	0.033	0.518	12.98	
2	NS	0.072	-0.06	0.204	28.13	
2	USOC	0.009	-0.014	0.032	53.86	
2	GS	0.012	-0.413	0.436	5.03	
**2**	**Overall**	**0.061**	**-0.039**	**0.161**		**48.11**
3	MCS	0.183	-0.005	0.371	13.72	
3	ALSPAC	-0.115	-0.331	0.101	12.26	
3	NS	0.043	-0.102	0.188	16.21	
3	USOC	0.002	-0.03	0.035	21.67	
3	ELSA	-0.177	-0.365	0.012	13.7	
3	GS	0.086	-0.338	0.51	5.41	
3	BiB	-0.238	-0.369	-0.107	17.04	
**3**	**Overall**	-0.042	-0.155	0.072		**76.78**

© 2022 Patel K et al. *JAMA Network Open.*

eTable 25. Meta-analyzed Regression Coefficients: Interactions; Education

Time	Study	SMD	Lower CI	Upper CI	Weight	I2
1	MCS	-0.008	-0.099	0.084	6.82	
1	ALSPAC	-0.129	-0.207	-0.051	8.28	
1	NS	-0.016	-0.194	0.161	2.44	
1	BCS70	-0.014	-0.088	0.06	8.83	
1	NCDS	0.002	-0.059	0.062	10.73	
1	NSHD	0.02	-0.065	0.106	7.45	
1	USOC	-0.02	-0.031	-0.008	19	
1	ELSA	-0.058	-0.136	0.02	8.27	
1	GS	-0.094	-0.154	-0.035	10.89	
1	TwinsUK	-0.095	-0.15	-0.04	11.71	
1	BiB	0	-0.106	0.106	5.57	
**1**	**Overall**	-0.041	-0.071	-0.012		**58.67**
2	MCS	0.134	0.028	0.241	11.06	
2	NS	-0.105	-0.223	0.013	10.34	
2	BCS70	-0.049	-0.13	0.031	12.84	
2	NCDS	-0.001	-0.061	0.059	14.13	
2	NSHD	0.248	0.076	0.42	7.33	
2	USOC	-0.014	-0.025	-0.002	16.11	
2	GS	-0.04	-0.106	0.027	13.73	
2	TwinsUK	-0.091	-0.145	-0.036	14.46	
**2**	**Overall**	**-0.005**	**-0.068**	**0.058**		**88.47**
3	MCS	0.06	-0.057	0.177	6.82	
3	ALSPAC	-0.138	-0.224	-0.053	9.59	
3	NS	-0.117	-0.243	0.009	6.21	
3	BCS70	-0.116	-0.203	-0.029	9.41	
3	NCDS	-0.014	-0.092	0.064	10.34	
3	NSHD	1.183	0.607	1.758	0.45	
3	USOC	-0.02	-0.033	-0.007	17.33	
3	ELSA	-0.011	-0.09	0.067	10.28	
3	GS	-0.089	-0.157	-0.02	11.45	
3	TwinsUK	-0.101	-0.155	-0.047	13.2	
3	BiB	0.033	-0.116	0.182	4.93	
**3**	**Overall**	**-0.05**	**-0.089**	**-0.011**		**67.41**

© 2022 Patel K et al. *JAMA Network Open.*

eTable 26. Inclusion and Exclusion Criteria by Cohort for Metaregressions and Sensitivity Analyses

Cohort	All Time Periods	Depression	Anxiety	Representative	Pre/1st Gap	Measure
**NSHD**	1	0	0	1	3	1
**BCS70**	1	0	0	1	2	2
**NCDS**	1	0	0	1	4	2
**NS**	1	0	0	1	3	1
**MCS**	1	0	0	1	1	3
**TwinsUK**	1	1	1	0	2	3
**BIB**	0	0	0	0	2	3
**ALSPAC**	0	1	1	0	2	3
**GS**	1	0	1	0	4	3
**USoc**	1	0	0	1	1	1
**ELSA**	0	1	0	1	1	3

Key:

All Time Periods	0 = Cohort has responses for time periods 1 and 3<br>1 = Cohort has responses for all time periods
Depression	0 = Cohort does not include specific measures for depression<br>1 = Cohort includes specific measures for depression
Anxiety	0 = Cohort does not include specific measures for depression<br>1 = Cohort includes specific measures for depression
Representative	0 = Cohort is representative of one UK country<br>1 = Cohort is representative of more than one UK country
Pre/1st Gap	1 = Pre-pandemic response was less than two years before the first pandemic response<br>2 = Pre-pandemic response was between two and five years years before the first pandemic response<br>3 = Pre-pandemic response was between five and seven years before the first pandemic response<br>4 = Pre-pandemic response was more than seven years before the first pandemic response
Measure	1 = GHQ measure of psychological distress<br>2 = Malaise measure of psychological distress<br>3 = Other measure of psychological distress

© 2022 Patel K et al. *JAMA Network Open.*

eTable 27. Meta-analyzed Interaction Coefficients

Modifier	Time Period	Number of Studies	Interaction Coefficient	Lower CI	Upper CI	I2
Sex (Female)	1	10	0.08	0.03	0.13	84.93
	2	8	0.07	0.02	0.12	75.70
	3	10	0.08	0.02	0.13	83.88
Ethnicity (Ethnic Minority)	1	7	-0.01	-0.13	0.11	85.80
	2	4	0.06	-0.04	0.16	48.11
	3	7	-0.04	-0.16	0.07	76.78
Education (No Degree)	1	11	-0.04	-0.07	-0.01	58.67
	2	10	-0.01	-0.07	0.06	88.47
	3	11	-0.05	-0.09	-0.01	67.41

© 2022 Patel K et al. *JAMA Network Open.*

eTable 28. Heterogeneity Explained by Measures Explored by Metaregression Analyses

Measure	Timepoint	Heterogeneity (I^2)	Heterogeneity Explained (%)
Time between pre-pandemic and first pandemic measure	1	98.22	1.00
	2	97.78	0.77
	3	99.17	1.05
General population representativeness	1	99.10	0.10
	2	95.17	3.38
	3	97.05	2.7
Type of measure for mental health used	1	98.17	1.05
	2	95.84	2.19
	3	98.41	0.83

© 2022 Patel K et al. *JAMA Network Open.*

eAppendix 4. Results of Binary High Psychological Distress Outcomes by Sociodemographic Factor

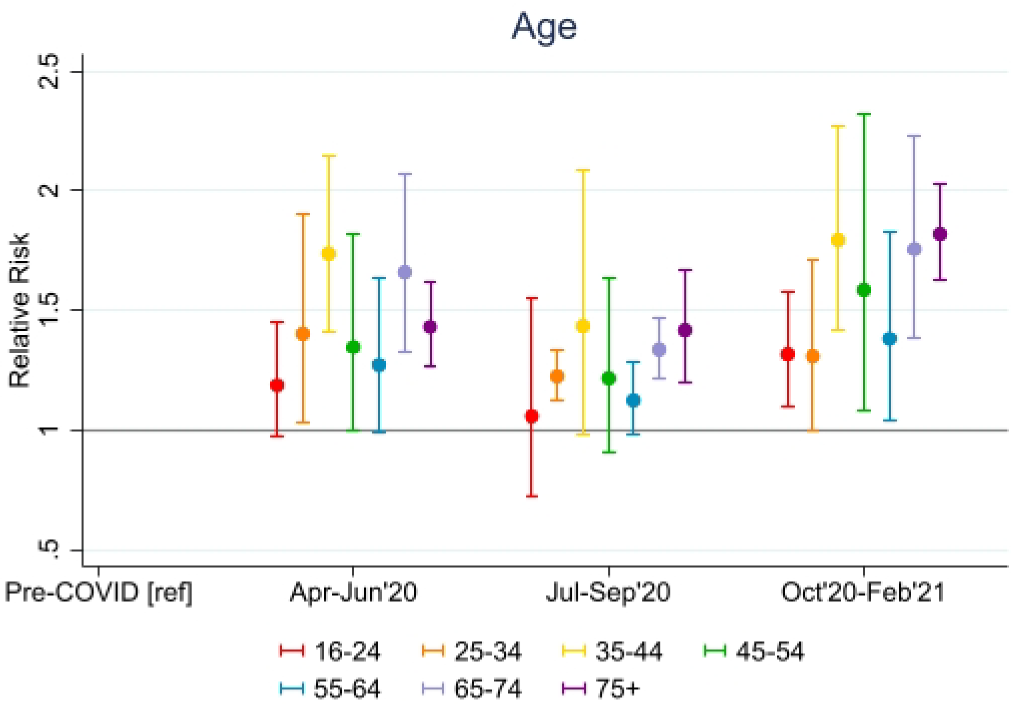

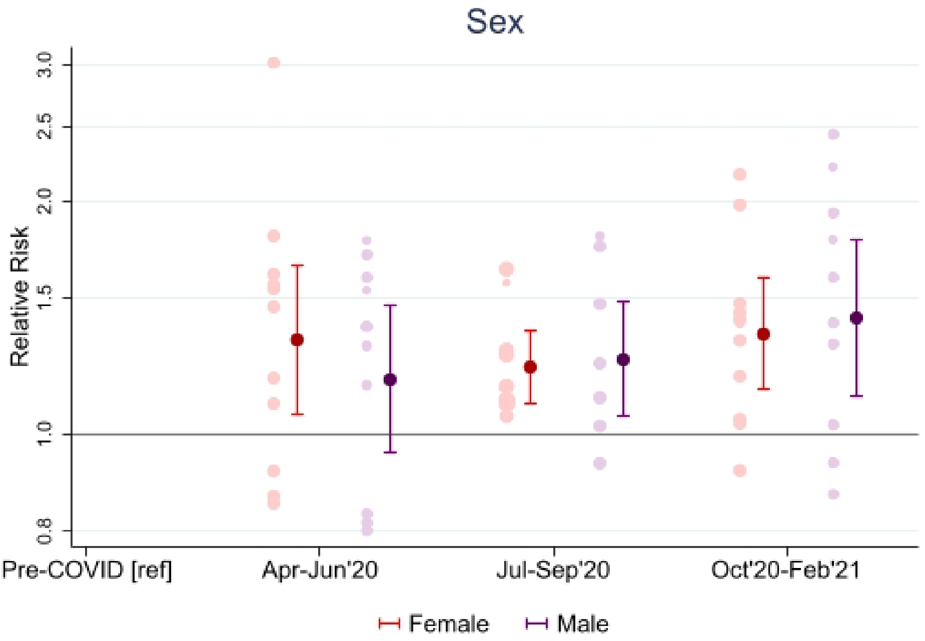

© 2022 Patel K et al. *JAMA Network Open.*

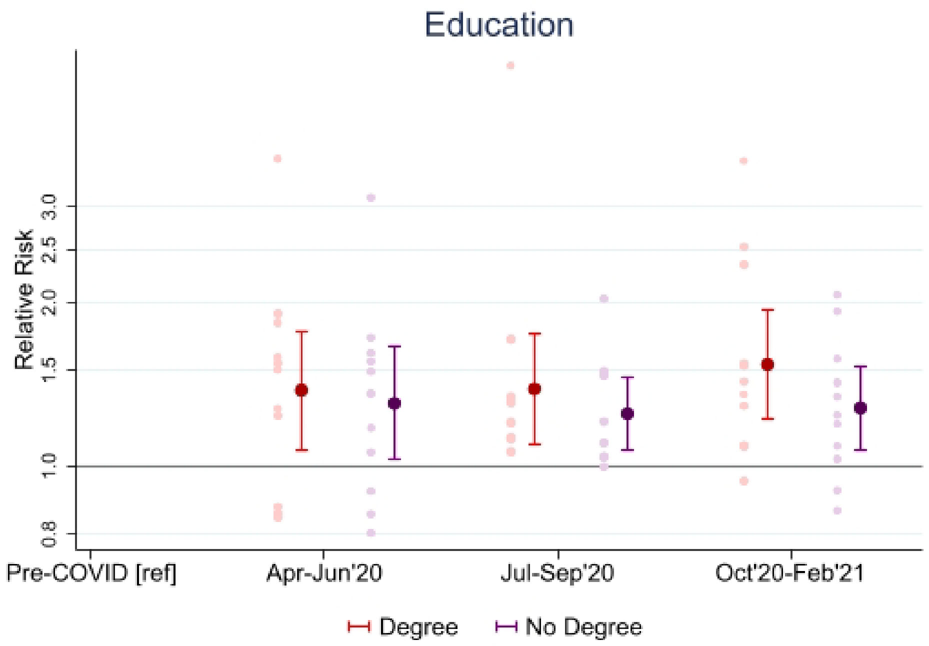

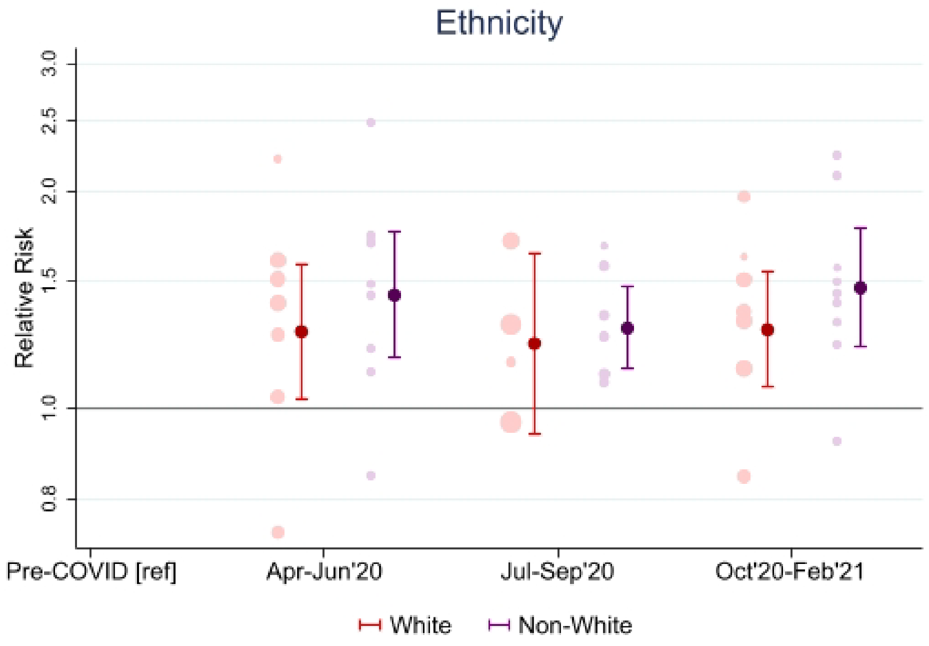

© 2022 Patel K et al. *JAMA Network Open.*

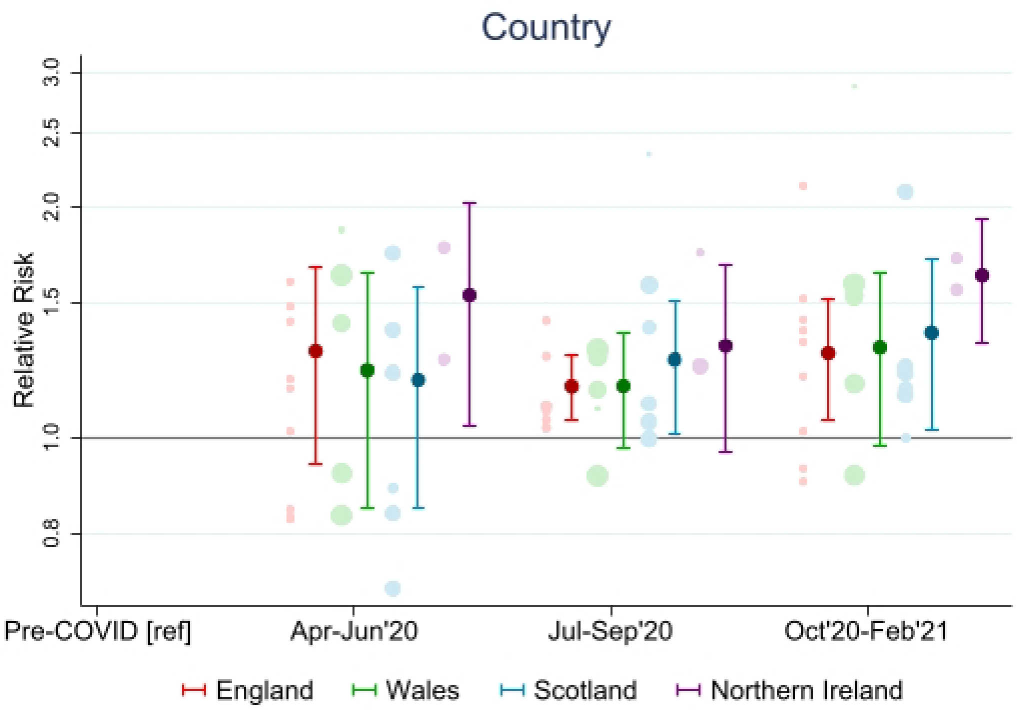

© 2022 Patel K et al. *JAMA Network Open.*

eAppendix 5. Meta-analyzed High Psychological Distress When One Cohort Is Removed

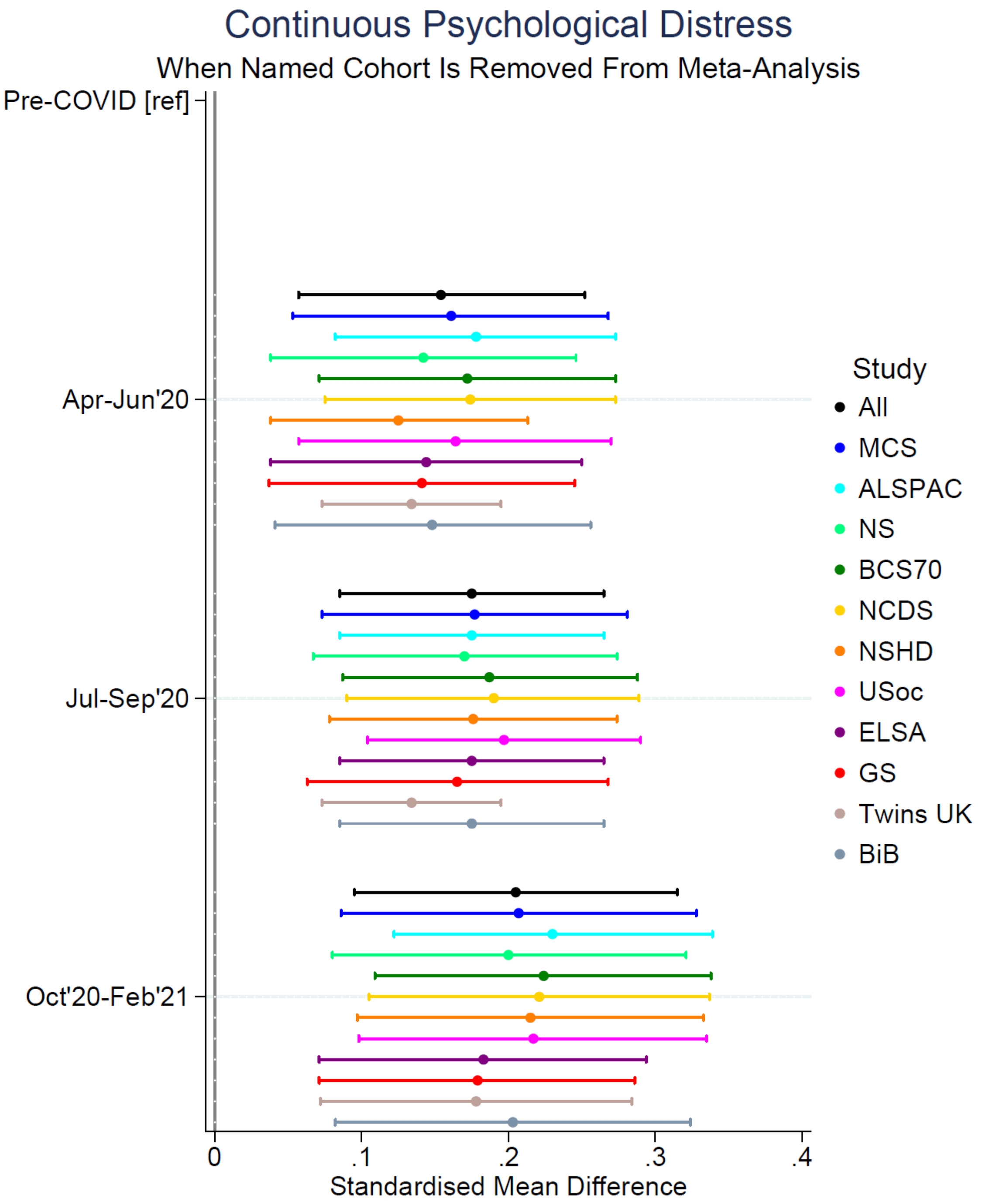

© 2022 Patel K et al. *JAMA Network Open.*

eAppendix 6. Funding Statements for Each Study

ALSPAC: The UK Medical Research Council and Wellcome (Grant Ref: 217065/Z/19/Z) and the University of Bristol provide core support for ALSPAC. A comprehensive list of grants funding is available on the ALSPAC website (http://www.bristol.ac.uk/alspac/external/documents/grant-acknowledgements.pdf). We are extremely grateful to all the families who took part in this study, the midwives for their help in recruiting them, and the whole ALSPAC team, which includes interviewers, computer and laboratory technicians, clerical workers, research scientists, volunteers, managers, receptionists and nurses. Part of this data was collected using REDCap, see the REDCap website for details: https://projectredcap.org/resources/citations/). This work was supported by Wellcome through the Wellcome Longitudinal Population Studies COVID-19 Secretariat and Steering Group (UK LPS COVID co-ordination, Grant Ref: 221574/Z/20/Z) and supported by the Elizabeth Blackwell Institute, University of Bristol, Wellcome Trust Institutional Strategic Support Fund and Rosetrees Trust (Grant Ref: 204813/Z/16/Z; R105121). ASFK is funded by an Economics and Social Research Council (ESRC) Postdoctoral Fellowship (ES/V011650/1).

USOC: Understanding Society is an initiative funded by the Economic and Social Research Council and various Government Departments, with scientific leadership by the Institute for Social and Economic Research, University of Essex, and survey delivery by NatCen Social Research and Kantar Public. The Understanding Society COVID-19 study is funded by the Economic and Social Research Council (ES/K005146/1) and the Health Foundation (2076161). The research data are distributed by the UK Data Service.

MCS, NS, BCS, NCDS, NSHD: The Millennium Cohort Study, Next Steps, 1970 British Cohort Study and 1958 National Child Development Study are supported by the Centre for Longitudinal Studies, Resource Centre 2015-20 grant (ES/M001660/1) and a host of other co-funders. The 1946 NSHD cohort is hosted by the the MRC Unit for Lifelong Health and Ageing funded by the Medical Research Council (MC_UU_00019/1Theme 1: Cohorts and Data Collection). The COVID-19 data collections in these five cohorts were funded by the UKRI grant Understanding the economic, social and health impacts of COVID-19 using lifetime data: evidence from 5 nationally representative UK cohorts (ES/V012789/1)

ELSA: The English Longitudinal Study of Ageing was developed by a team of researchers based at University College London, NatCen Social Research, the Institute for Fiscal Studies, the University of Manchester and the University of East Anglia. The data were collected by NatCen Social Research. The funding is currently provided by the National Institute on Aging in the US, and a consortium of UK government departments coordinated by the National Institute for Health Research. Funding has also been received by the Economic and Social Research Council. The English Longitudinal Study of Ageing Covid-19 Substudy was supported by the UK Economic and Social Research Grant (ESRC) ES/V003941/1.

GS: Generation Scotland received core support from the Chief Scientist Office of the Scottish Government Health Directorates [CZD/16/6] and the Scottish Funding Council [HR03006]. Genotyping of the GS:SFHS samples was carried out by the Genetics Core Laboratory at the Wellcome Trust Clinical Research Facility, Edinburgh, Scotland and was funded by the Medical Research Council UK and the Wellcome Trust (Wellcome Trust Strategic Award "STratifying Resilience and Depression Longitudinally" (STRADL)

© 2022 Patel K et al. *JAMA Network Open.*

Reference 104036/Z/14/Z). Generation Scotland is funded by the Wellcome Trust (216767/Z/19/Z).

TwinsUK: TwinsUK receives funding from the Wellcome Trust (WT212904/Z/18/Z), the National Institute for Health Research (NIHR) Biomedical Research Centre based at Guy's and St Thomas' NHS Foundation Trust and King's College London. The TwinsUK COVID-19 personal experience study was funded by the King's Together Rapid COVID-19 Call award, under the projects original title 'Keeping together through coronavirus: The physical and mental health implications of self-isolation due to the Covid-19 TwinsUK is also supported by the Chronic Disease Research Foundation and Zoe Global Ltd. The funders had no role in study design, data collection and analysis, decision to publish, or preparation of the manuscript.

BiB: Born in Bradford has received funding from the Wellcome Trust [101597] a joint grant from the UK Medical Research Council (MRC) and UK Economic and Social Science Research Council (ESRC) [MR/N024391/1] the National Institute for Health Research under its Applied Research Collaboration Yorkshire and Humber [NIHR200166]. This study has been funded through The Health foundation COVID-19 Award [2301201].

© 2022 Patel K et al. *JAMA Network Open.*

eAppendix 7. Further Study Information

ALSPAC: Pregnant women resident in Avon, UK with expected dates of delivery 1st April 1991 to 31st December 1992 were invited to take part in the study. The initial number of pregnancies enrolled is 14,541 (for these at least one questionnaire has been returned or a "Children in Focus" clinic had been attended by 19/07/99). Of these initial pregnancies, there was a total of 14,676 foetuses, resulting in 14,062 live births and 13,988 children who were alive at 1 year of age. When the oldest children were approximately 7 years of age, an attempt was made to bolster the initial sample with eligible cases who had failed to join the study originally. As a result, when considering variables collected from the age of seven onwards (and potentially abstracted from obstetric notes) there are data available for more than the 14,541 pregnancies mentioned above. The number of new pregnancies not in the initial sample (known as Phase I enrolment) that are currently represented on the built files and reflecting enrolment status at the age of 24 is 913 (456, 262 and 195 recruited during Phases II, III and IV respectively), resulting in an additional 913 children being enrolled. The phases of enrolment are described in more detail in the cohort profile paper and its update (see footnote 4 below). The total sample size for analyses using any data collected after the age of seven is therefore 15,454 pregnancies, resulting in 15,589 foetuses. Of these 14,901 were alive at 1 year of age. A 10% sample of the ALSPAC cohort, known as the Children in Focus (CiF) group, attended clinics at the University of Bristol at various time intervals between 4 to 61 months of age. The CiF group were chosen at random from the last 6 months of ALSPAC births (1432 families attended at least one clinic). Excluded were those mothers who had moved out of the area or were lost to follow-up, and those partaking in another study of infant development in Avon12.

eReferences

1. Kessler RC, Andrews G, Colpe LJ, et al. Short screening scales to monitor population prevalences and trends in non-specific psychological distress. *Psychological Medicine* 2002;32(6):959-76.

2. Rutter M, Tizard J, Whitmore K. Education, health and behaviour: Longman Publishing Group 1970.

3. Goldberg D. Manual of the general health questionnaire: Nfer Nelson 1978.

4. Kroenke K, Spitzer RL. The PHQ-9: a new depression diagnostic and severity measure. *Psychiatric Annals* 2002;32(9):509-15.

5. Angold A, Costello E, Messer S, et al. Development of a short questionnaire for use in epidemiological studies of depression in children and adolescents. *International Journal of Methods in Psychiatric Research* 1995;5:237-49.

6. Cox JL, Holden JM, Sagovsky R. Detection of postnatal depression: development of the 10-item Edinburgh Postnatal Depression Scale. *The British Journal of Psychiatry* 1987;150(6):782-86.

7. Matthey S, Barnett B, Kavanagh DJ, et al. Validation of the Edinburgh Postnatal Depression Scale for men, and comparison of item endorsement with their partners. *Journal of Affective Disorders* 2001;64(2-3):175-84.

8. Radloff LS. The CES-D scale: A self-report depression scale for research in the general population. *Applied Psychological Measurement* 1977;1(3):385-401.

9. Zigmond AS, Snaith RP. The hospital anxiety and depression scale. *Acta Psychiatrica Scandinavica* 1983;67(6):361-70.

10. Kroenke K, Strine TW, Spitzer RL, Williams JBW, Berry JT, Mokdad AH: The PHQ-8 as a measure of current depression in the general population. *Journal of Affective Disorders* 2009, 114(1):163-173.

11. Löwe B, Decker O, Müller S, Brähler E, Schellberg D, Herzog W, Herzberg PY: Validation and Standardization of the Generalized Anxiety Disorder Screener (GAD-7) in the General Population. *Medical Care* 2008, 46(3):266-274.

12. Northstone K, Lewcock M, Groom A, Boyd A, Macleod J, Timpson NJ, Wells N. The Avon Longitudinal Study of Parents and Children (ALSPAC): an updated on the enrolled sample of index children in 2019. *Wellcome Open research* 2019; 4:51.

© 2022 Patel K et al. *JAMA Network Open.*
